# Effects of Nutritional Supplements on Explosive Lower Limb Performance in Volleyball Players: A Systematic Review and Network Meta-Analysis

**DOI:** 10.3390/nu17233702

**Published:** 2025-11-26

**Authors:** Haoyu Du, Shuning Liu, Mu Li, Kai Zhao, Wei Jiang, Ting You, Zheng Wang, Dixin Zou, Jingdan Shu, Chang Liu

**Affiliations:** 1 Department of Physical Education, China Agricultural University, Beijing 100193, China; duhaoyu0802@163.com; 2 School of Sport Science, Beijing Sport University, Beijing 100084, China; 2023013553@bsu.edu.cn (S.L.); t.you@bsu.edu.cn (T.Y.); 3 Volleyball Administrative Center, Beijing Muxiyuan Sports Technical School, Beijing 100075, China; meganwong61@gmail.com; 4 China Volleyball College, Beijing Sport University, Beijing 100084, China; 2430@bsu.edu.cn; 5 Career and Entrepreneurship Services, Beijing 100084, China; wangzheng@bsu.edu.cn; 6 Institute of Chinese Materia Medica, China Academy of Chinese Medical Sciences, Beijing 100700, China; zoudixin@163.com

**Keywords:** volleyball, nutritional supplements, explosive lower limb power, network meta-analysis

## Abstract

**Background:** Explosive lower limb power, a critical determinant of success in volleyball, is a prime candidate for focused research. Consequently, nutritional supplements are commonly employed to gain an ergogenic edge. Despite this widespread practice, a comprehensive and evidence-based ranking of supplements for improving this key attribute is lacking, leaving athletes and practitioners without targeted evidence to guide decisions. **Methods:** PubMed, Web of Science, CENTRAL, and Embase were searched from inception to 1 August 2025. We included RCTs in volleyball athletes (≥14 years) with ≥1-week interventions and relevant explosive lower limb performance outcomes. A Bayesian NMA estimated effects with 95% credible intervals (CrIs) and SUCRA; certainty was appraised with CINeMA. **Results:** A total of 35 RCTs (*n* = 838 volleyball athletes) examining 13 different supplements were included. The results indicated that β-alanine was associated with the greatest improvement in vertical jump (MD 4.6; 95% CrI 1.2–7.8), followed by creatine (MD 3.7; 0.57–6.9) and caffeine (MD 2.1; 0.06–4.1). It also appeared to be the most promising method for substantially increasing lower limb peak power (SMD 1.1; 0.21–2.0). No statistically significant improvement was found in lower limb mean power, and no serious adverse events were reported. **Conclusions:** Among volleyball athletes, β-alanine appeared most promising effective supplement for enhancing key components of explosive lower limb performance, specifically vertical jump height and peak power. Creatine and caffeine are also effective for improving vertical jump height. However, these findings are based on low-to-moderate certainty of evidence; they should be interpreted with caution and regarded as preliminary. Supplement strategies should be individualized by age and competitive level; further, high-quality, standardized randomized controlled trials are warranted to validate these initial observations.

## 1. Introduction

Volleyball is one of the most popular team sports worldwide, characterized by high-intensity, intermittent, and explosive movements interspersed with brief recovery periods [1]. Athletes typically perform bouts of intense activity lasting 3–9 s, followed by 10–20 s of recovery, with rallies rarely exceeding 12 s [2]. To sustain performance during repeated high-intensity rallies, players rely predominantly on anaerobic energy systems [3,4].

Lower-limb performance—especially vertical jump height and power—is an important determinant of scoring ability and, by extension, match outcomes in volleyball [5]. Teams win most points via attacks, and attack effectiveness is a key predictor of victory [6]; jump height (serve/block/spike) positively relates to play efficiency and final match status [7], and higher-level or starting players typically show superior vertical-jump ability [8].

Dietary supplements are defined as foods, food components, nutrients, or non-food compounds consumed in addition to the habitual diet with the goal of achieving specific health or performance benefits [9]. By supplying energy substrates that are difficult to produce endogenously, supplements can play a critical role in recovery and performance enhancement [10,11]. According to the International Society of Sports Nutrition (ISSN), strong evidence supports the efficacy of several supplements, including creatine, caffeine, β-alanine, β-hydroxy-β-methylbutyrate (HMB), and branched-chain amino acids (BCAAs) [10]. For example, creatine supplementation has consistently been shown to improve short-duration, high-intensity exercise and resistance training outcomes by enhancing both absolute and relative anaerobic capacity [12,13]. Caffeine has been reported to improve jumping, sprinting, agility, cognitive function, and competitive performance in team-sport athletes [14,15]. β-alanine can increase buffering capacity, thereby improving endurance and resistance to fatigue [16]; amino acids are believed to support immunity, muscle growth, and angiogenesis [17], whereas protein supplementation can attenuate muscle damage, promote recovery, and improve resistance-training adaptations [18]. Surveys indicate that nearly 89% of volleyball players routinely use sports supplements [19]. Importantly, when used appropriately, supplements can offer performance benefits without compromising health or increasing the risk of doping violations [9].

Given the growing global popularity of volleyball and its distinct energetic demands, it is essential to examine factors that influence performance in this sport [20]. Such work has direct implications for improving the quality of training and competition. However, evidence specific to volleyball players is limited and scattered across studies focusing on different supplements and outcomes. Although the review by Hernández-Landa et al. covered a wide range of supplements, it only provided a qualitative summary and did not identify significant differences in the effects of individual supplements [21]. This gap constrains the development of evidence-based guidance for supplement use in practice [10,22]. In contrast to previous narrative reviews, this work is the first to apply a network meta-analysis to volleyball supplements, providing relative effect estimates and probabilistic rankings (SUCRA) that can guide evidence-based decision-making [23]. Due to volleyball athletes rely heavily on jumping performance [24], this review aims to compare the effects of commonly used nutritional supplements on explosive lower limb performance in volleyball players using a network meta-analysis. We hypothesized that certain supplements (e.g., β-alanine and creatine) would show greater improvements in vertical jump performance compared to others. We further assessed relative effects, SUCRA, and the certainty of evidence (CINeMA). Our goal was to provide athletes, coaches, and practitioners with evidence-based, sport-specific recommendations for supplement use.

## 2. Materials and Methods

### 2.1. Reporting Guideline and Protocol Registration

This review was conducted and reported in accordance with PRISMA 2020 and the PRISMA extension for Network Meta-Analyses (PRISMA-NMA). The protocol was prospectively registered in PROSPERO (CRD420251084241).

### 2.2. Search Strategy

A systematic search was conducted in PubMed, Web of Science Core Collection, the Cochrane Central Register of Controlled Trials (CENTRAL), and Embase from inception to 1 August 2025, with no language restrictions. The full search syntax for PubMed was as follows: (“volleyball”[MeSH Terms] OR “volleyball”[All Fields]) AND (“caffeine”[All Fields] OR “beta-alanine”[All Fields] OR “magnesium”[All Fields] OR “grape juice”[All Fields] OR “iron”[All Fields] OR “creatine”[All Fields] OR “nutritional supplementation”[All Fields]) AND (“performance”[All Fields] OR “jump”[All Fields] OR “power”[All Fields] OR “strength”[All Fields] OR “skill”[All Fields]) AND (“randomized controlled trial”[Publication Type] OR “Randomized Controlled Trial”[MeSH Terms] OR “randomized”[All Fields]). Complete search strategies for all databases are provided in Supplementary Material S1 Tables S1–S4. Two reviewers (H.D. and S.L.) independently executed the search and screened records; disagreements were resolved by a third reviewer (M.L.), with consultation of a senior author (C.L.) when necessary. The full electronic search strategies are provided in Supplementary Material S1.

### 2.3. Eligibility Criteria

Inclusion criteria. We included peer-reviewed, full-text randomized controlled trials enrolling volleyball athletes aged ≥14 years; participants of both sexes were included. Eligible interventions were pre-specified, commonly used dietary supplements intended to enhance lower limb performance; a total of 13 supplements met the inclusion criteria through a comprehensive literature search: β-alanine, creatine, caffeine, branched-chain amino acids, β-hydroxy-β-methylbutyrate, magnesium, beetroot juice, protein, carbohydrate, energy drinks, probiotics, L-citrulline, and grape juice. Comparators were placebo or usual practice and, where applicable, active comparators (other eligible supplements). Primary outcomes were objective, volleyball-relevant performance measures assessed with validated tests—vertical jump, lower limb peak power, lower limb mean power—plus adverse events. For interpretability, units and effect directions were harmonized a priori.

Exclusion criteria. We excluded non-randomized designs; non-volleyball samples; trials involving prohibited substances per the contemporaneous WADA Prohibited List; studies reporting only biochemical or purely subjective outcomes without any objective performance endpoint; secondary research (systematic reviews, narrative reviews, and meta-analyses), conference abstracts without full data, and studies with insufficient quantitative information for synthesis. For cross-over RCT, an adequate washout was required; multi-arm trials were eligible if data permitted appropriate analysis.

### 2.4. Study Selection

Records retrieved from all databases were imported into EndNote 20 for automated and manual de-duplication. Screening proceeded in three stages: (1) Title or abstract screening was performed by two independent reviewers (H.D. and S.L.), with uncertain records deliberately retained to minimize erroneous exclusions, and (2) any disagreements between the two independent reviewers were resolved through discussion with a third reviewer (M.L.). We did not formally calculate inter-rater agreement (e.g., kappa), but consensus was reached on all included studies after discussion, and (3) the final inclusion was made according to the pre-specified criteria.

### 2.5. Data Extraction

Two reviewers (H.D. and S.L.) independently extracted data using a pilot-tested electronic form; disagreements were resolved by a third reviewer (C.L.). Extracted domains included the following: (1) study characteristics (publication year, country/region, design, and study period, where applicable); (2) participant characteristics (sample size, age, sex, competitive level, height, and body mass); (3) intervention details (supplement identity, dose and timing, and duration); (4) performance outcomes (vertical jump, lower limb peak power, and lower limb mean power); and (5) adverse events. When data were available only in graphical form, values were digitized using WebPlotDigitizer v5.2. Prior to synthesis, units and effect directions were harmonized (e.g., vertical jump in cm, agility time in s).

The primary outcome was vertical jump (cm), chosen for its direct relevance to spiking or blocking performance [1] and its widespread use with good test–retest reliability; secondary outcomes included lower limb peak power and lower limb mean power. When multiple jump tests were reported, we prioritized the countermovement jump (CMJ) as the primary vertical jump measure due to its widespread use and excellent reliability in volleyball performance testing, followed by the squat jump (SJ) if CMJ was not reported, and other jump tests thereafter. Effect sizes used MD for the same units or SMD otherwise, with directions harmonized so that higher values indicate better performance.

### 2.6. Risk of Bias and Certainty Assessment

Risk of bias for RCT was appraised using RoB 2.0, covering five domains: randomization process, deviations from intended interventions, missing outcome data, measurement of the outcome, and selection of the reported result. Two reviewers performed independent domain-level judgments, with overall risk generated via the RoB 2.0 algorithm; disagreements were adjudicated by a third reviewer. Publication bias was evaluated using funnel plots and Egger’s regression test for outcomes with ≥10 studies. Confidence of evidence for network estimates was evaluated using CINeMA, considering within-study bias, reporting bias, indirectness, imprecision, heterogeneity, and incoherence, with an overall certainty rating reported for each outcome. Transitivity was assessed by comparing the distribution of potential effect modifiers (e.g., age, competitive level, dosing regimens, and testing protocols) across studies contributing direct and indirect evidence.

### 2.7. Data Synthesis and Analysis

We conducted a random-effects network meta-analysis (NMA) under a Bayesian framework, implemented in R (v3.6.1) using gemtc (v0.8-2), which interfaces with JAGS (v4.3.0) for Markov chain Monte Carlo (MCMC) estimation. A Bayesian approach enables flexible modeling, coherent borrowing of strength across the evidence network, and probabilistic interpretation of treatment effects and ranking, which can be more informative for clinicians and coaches than traditional *p*-values.

Continuous outcomes were analyzed as mean differences (MD) when scales were common; otherwise, standardized mean differences (SMD; Hedges’ g) were used. Units were harmonized a priori: vertical jump (cm), lower limb peak power, and lower limb mean power (W). Outcome directions were aligned so that higher values indicated improvement. When trials reported multiple post-intervention assessments, we used the protocol-defined primary time window (earliest post-intervention), and we examined later time points in sensitivity analyses. Where only *p*-values, t-statistics, or CI were reported, we reconstructed standard errors and 95% confidence intervals (CIs) using established transformations; network estimates from the Bayesian model are reported with 95% credible intervals (CrIs). We interpreted I^2^ < 40% as low heterogeneity, 40–75% as moderate, and >75% as substantial heterogeneity, in line with Cochrane guidelines.

Crossover trials were included only if an adequate washout period was reported; data from the end of each intervention period (after washout) were treated as if from a parallel design. For multi-arm trials, we either merged equivalent intervention arms (using established formulas for means and SDs to avoid double-counting controls) or, if arms were distinct, we included them as separate nodes with the Bayesian model accounting for the shared control group correlation.

$$SD=\sqrt{\frac{\left(N_{1}-1\right)SD_{1}^{2}+\left(N_{2}-1\right)SD_{2}^{2}+\frac{N_{1}N_{2}}{N_{1}+N_{2}}\left(M_{1}^{2}+M_{2}^{2}-2M_{1}M_{2}\right)}{N_{1}+N_{2}-1}}$$

Model specification and selection. We fitted both fixed-effect and random-effects Bayesian NMA models. Model adequacy was compared using the deviance information criterion (DIC) together with summaries of residual deviance and leverage; a DIC difference <3–5 was considered negligible, otherwise the model with the lower DIC was preferred. Given between-study heterogeneity in populations, protocols, and dosing schedules, the random-effects model was retained for all primary analyses.

Priors and MCMC. Unless stated otherwise, treatment effects used weakly informative Normal (0, 10^4^) priors and the heterogeneity SD used a Uniform prior; sensitivity analyses with alternative heterogeneity priors (e.g., half-Normal) yielded similar inferences. We ran 4 parallel chains with 5000 adaptation/burn-in iterations followed by 25,000 sampling iterations per chain. Convergence was assessed by trace/density plots and Gelman–Rubin R-hat (all ≤1.05), and by ensuring Monte Carlo SE was small relative to the posterior SD (target MCSE < 10%). Effective sample sizes are reported in the Supplement. Sensitivity analyses using alternative priors for heterogeneity (e.g., half-Normal distributions) were conducted to assess robustness, and the results remained consistent across specifications.

Transitivity, consistency, and ranking. The transitivity assumption was evaluated through both qualitative and quantitative approaches. First, we systematically compared the distributions of potential effect modifiers (including age, sex, training status, and baseline performance) across treatment comparisons. Second, we employed statistical tests for inconsistency (design-by-treatment interaction and node-splitting analyses) as indirect assessments of transitivity. The absence of significant inconsistency (all *p* > 0.05) in these analyses supports the validity of the transitivity assumption in our network. Global inconsistency was assessed with the design-by-treatment interaction test, and local inconsistency was checked via node-splitting (with a threshold *p*-value for significance). Treatments were ranked using SUCRA (Surface Under the Cumulative Ranking) alongside mean ranks and a 95% CrI; SUCRA values range from 0% to 100%, where 100% indicates a treatment that is certain to be the top performer and 0% indicates it is certain to be the worst performance, and rankings were interpreted as probabilistic summaries, not as evidence of statistical significance per se.

### 2.8. Sensitivity Analyses, Subgroups, and Network Meta-Regression

Sensitivity analyses. We (i) excluded trials judged overall high risk of bias by RoB 2.0 (two independent reviewers; with any disagreements resolved by a third independent reviewer); and (ii) explored an expanded network that incorporated trials from sports with matched testing protocols and effort durations (primary: badminton, and secondary: basketball/soccer). For (ii), we required identical test definitions/units/directions and examined transitivity by comparing age, competitive level, test protocol, and dosing regimen across direct/indirect contrasts. We interpreted this expansion exploratorily, focusing on whether treatment rankings and posterior means were materially altered.

Subgroups. We compared elite vs. non-elite athletes (a priori definitions based on competition level) and ≤21 vs. >21 years (aligned with FIVB adult–youth categorization) [25]. We report, for each split, the number of trials/participants and caution that subgroup inferences are underpowered where counts are small.

Network meta-regression. We fitted a Bayesian NMA with treatment–covariate interactions to test for effect modification by age group and competitive level. Covariates were mean-centered. We modeled interactions as exchangeable across treatments and used weakly informative priors on the interaction terms. Correlations from multi-arm trials were retained in the model. Given the limited number of trials, we prioritized parsimonious models and assessed the sensitivity of our conclusions to the choice of prior distributions.

### 2.9. Patient and Public Involvement

Patients or members of the public were not involved in the design, conduct, reporting, or dissemination of this research because it synthesizes previously published trials.

## 3. Results

### 3.1. Study Selection and Characteristics

**Figure 1:**
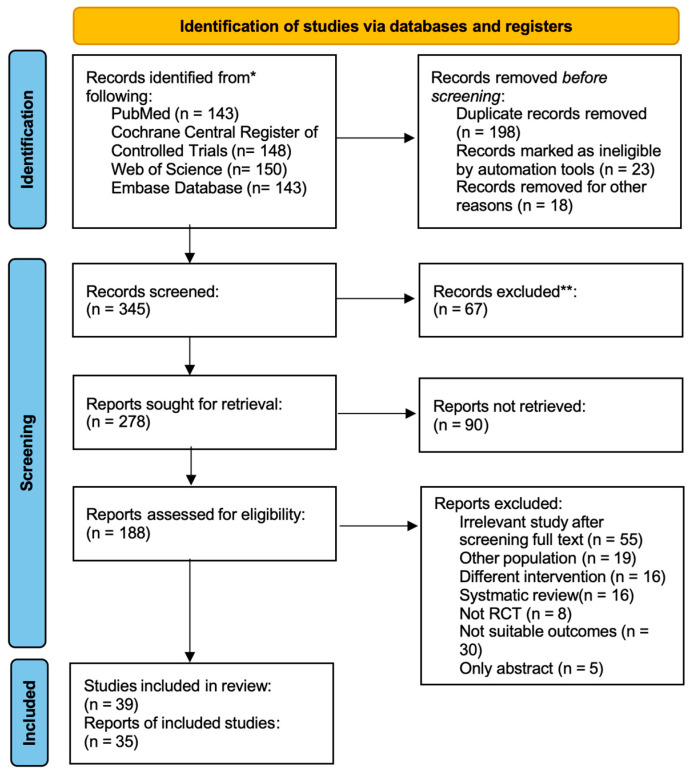
PRISMA flow diagram of study selection for supplements and physical performance in volleyball players.* Records were identified from multiple databases; numbers for each database are reported separately. ** Records excluded after screening titles and abstracts.

The database search yielded 584 records (Figure 1). After de-duplication and title or abstract screening, 186 full-text articles were assessed for eligibility (Figure S1). The 35 included RCTs enrolled a total of 838 volleyball athletes, with sample sizes ranging from 8 to 48 participants per study (approximately 65% male). The interventions spanned durations of 1 to 10 weeks, and 13 distinct supplements were evaluated. The most frequently investigated supplements were caffeine (12 trials; 3–6 mg/kg). A detailed breakdown of all supplement doses and regimens is provided in Table 1. Participants had a mean age of 20.6 years (SD = 2.09), a mean body mass of 75.4 kg (SD = 7.7), and a mean stature of 180.9 cm (SD = 7.9); approximately 65% were male, and the mean sport-specific training experience exceeded 3 years (Table 1).

Sixteen supplements were initially identified; coenzyme Q10, iron, and zinc were excluded owing to the absence of eligible performance outcomes or the lack of a placebo-controlled comparator [61,62,63], and the study by Kartashev et al. was also excluded because of the unsuitable outcomes provided [64]. The final evidence network encompassed 13 interventions: branched-chain amino acids, β-alanine, caffeine, carbohydrates, β-Hydroxy-β-Methylbutyrate, creatine, magnesium, L-citrulline, beetroot, protein, energy drinks, and the placebo.

### 3.2. Risk of Bias, Confidence of Evidence, and Consistency

**Figure 2:**
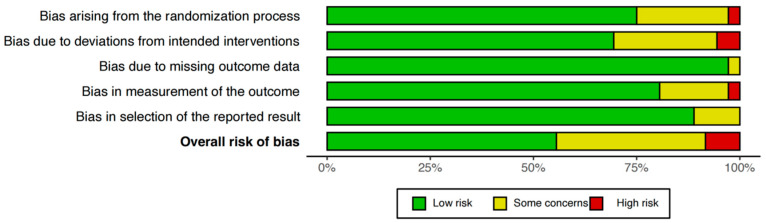
Assessment of the risk of bias of studies included in the network meta-analysis.

Domain-level risk of bias (RoB 2.0) is summarized in Figure 2. The most frequent limitations were incomplete reporting of allocation concealment and blinding (participants, personnel, and outcome assessors), and, in some cases, insufficient description of random sequence generation. Of the 35 RCTs assessed, 26 (74.3%) were deemed low risk in the randomization process domain; 25 (71.4%) were deemed low risk for deviations from intended interventions; 34 (97.1%) had no missing outcome data; and 30 (82.9%) were judged low risk for measurement of outcomes. Selective reporting was suspected in 4 (11.4%) studies, and 3 (8.6%) were rated overall high risk of bias (Figure 2, Supplementary Material S2 Figure S2).

These analyses revealed no evident asymmetry in the funnel plots, and the Egger’s test results (*p* > 0.05 for both outcomes) indicated no significant publication bias. For lower limb mean power, the limited number of available studies (<10) precluded a meaningful Egger’s test, though the corresponding funnel plot also showed no obvious asymmetry. All funnel plots and detailed statistical results have been provided in Supplementary Material S13.

Using CINeMA, confidence of evidence for network estimates was predominantly moderate or low (Supplementary Material S9), with most downgrades attributable to imprecision (limited sample sizes and wide credible intervals), and, in selected comparisons, within-study bias and incoherence. The transitivity assumption appeared reasonable: distributions of key effect modifiers (e.g., age, sex, and competitive level) were broadly comparable across treatment contrasts, supporting the validity of indirect estimates (Supplementary Material S9 Tables S9.1–S9.3).

### 3.3. Synthesis of Results

All Bayesian models demonstrated good convergence (Gelman–Rubin R-hat ≤ 1.05, with adequate effective sample sizes). Convergence diagnostics such as trace plots and R-hat values for all parameters are provided in Supplementary Materials S3 and S4. Node-splitting analyses detected no statistically significant local inconsistency for vertical jump, lower limb peak power (*p* > 0.05; Supplementary Material S5 Figures S5.1 and S5.2). For lower limb mean power, formal inconsistency testing was not feasible because the corresponding subnetwork lacked closed loops; results for this outcome should therefore be interpreted with caution.

#### 3.3.1. Vertical Jump

**Figure 3:**
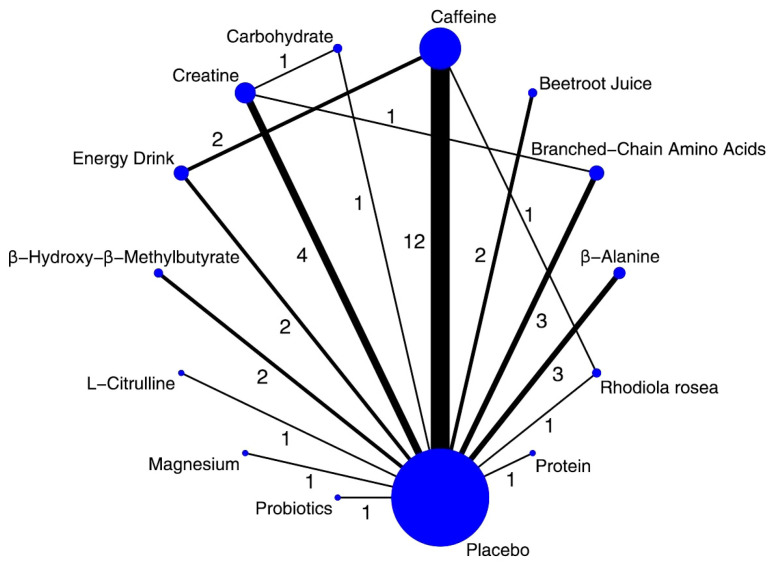
Network of available comparisons of nutritional supplements and placebo for vertical jump height. The size of the nodes is proportional to the number of trial participants, and the thickness of the line connecting the nodes is proportional to the randomized number of trial participants, directly comparing the two treatments. Numbers on lines indicate the number of trials in that head-to-head comparison.

**Figure 4:**
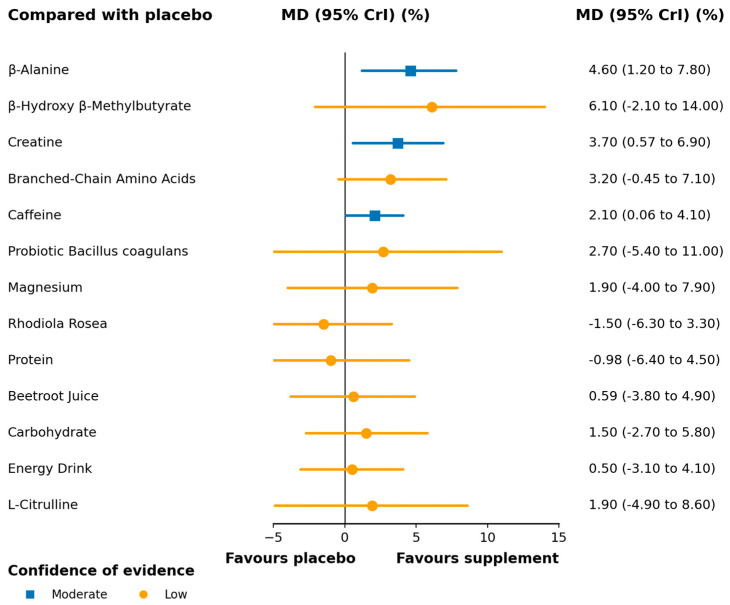
Forest plot of network effect sizes between nutritional supplements and placebo for vertical jump height. According to the network confidence meta-analysis (CINeMA) framework, the certainty of evidence is visually represented in the forest map, with varying colors indicating different confidence levels. The complete CINeMA assessments are shown in Supplementary Material S9. MD, mean difference; CrI, credible interval.

A total of 32 trials, including 593 participants, contributed data to this outcome, which was synthesized as mean differences (MDs). The network geometry is depicted in Figure 3. In the relative ranking of interventions, β-alanine was associated with the largest improvement (MD 4.60; 95% CrI 1.20–7.80; SUCRA 79.8%; moderate confidence of evidence), followed by creatine (MD 3.70; 0.57–6.90; SUCRA 71.1%; moderate) and caffeine (MD 2.10; 0.06–4.10; SUCRA 50.9%; moderate). Effects for other interventions were imprecise, with credible intervals including the null value (Figure 4). Full pairwise estimates and descriptive SUCRA are provided in Supplementary Tables S7.1 and S8.1; CINeMA rated overall certainty for this outcome as low to moderate, primarily downgraded for imprecision (Supplementary Table S9.1).

#### 3.3.2. Lower Limb Peak Power

**Figure 5:**
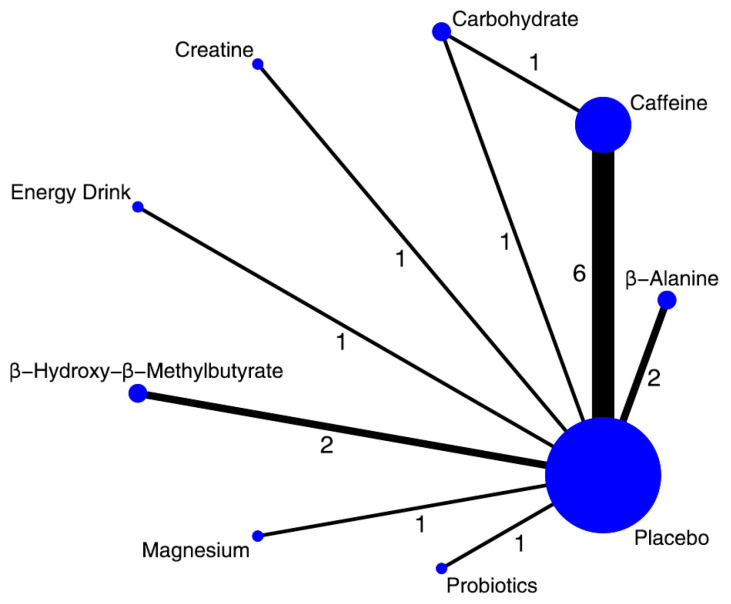
Network of available comparisons between nutritional supplements and placebo for lower limb peak power. The size of the nodes is proportional to the number of trial participants, and the thickness of the line connecting the nodes is proportional to the randomized number of trial participants, directly comparing the two treatments. Numbers represent the number of trials contributing to each treatment comparison.

**Figure 6:**
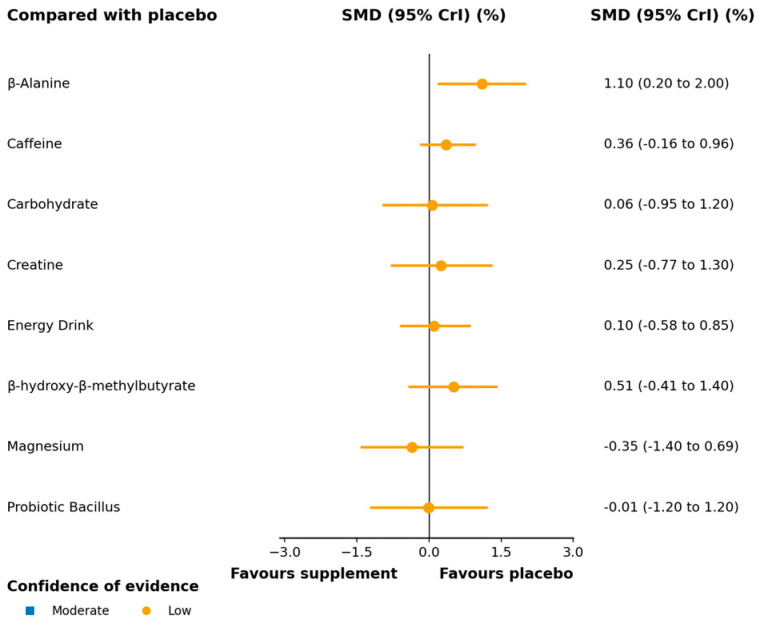
Forest plot of network effect sizes between nutritional supplements and placebo for lower limb peak power. Certainty of evidence is visually represented in the forest map, with the color indicating confidence level. The complete CINeMA assessments are shown in Supplementary Material S9. SMD, standardized mean difference; CrI, credible interval.

Fourteen trials (*n* = 394) were pooled using standardized mean differences (SMDs). Network geometry for this outcome is provided in Figure 5. β-alanine was superior to placebo (SMD 1.10; 95% CrI 0.21–2.00; moderate); other interventions did not demonstrate effects supported by the data, with credible intervals spanning the null (Figure 6). The SUCRA ranking, which provides a descriptive hierarchy of efficacy, ordered the supplements as follows: β-alanine, HMB, caffeine, and creatine (Supplementary Table S8.2). Pairwise estimates and SUCRA ranks are reported in Supplementary Tables S7.2 and S8.2;. CINeMA certainty was low, mainly due to imprecision (Supplementary Table S9.2).

#### 3.3.3. Lower Limb Mean Power

**Figure 7:**
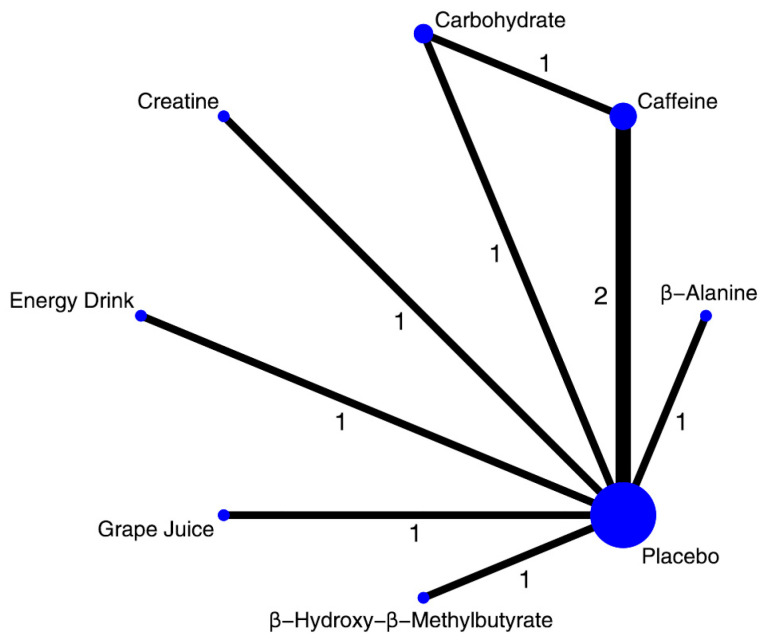
Network of available comparisons between nutritional supplements and placebo for lower limb mean power. The size of the nodes is proportional to the number of trial participants, and the thickness of the line connecting the nodes is proportional to the randomized number of trial participants, directly comparing the two treatments. Numbers represent the number of trials contributing to each treatment comparison.

**Figure 8:**
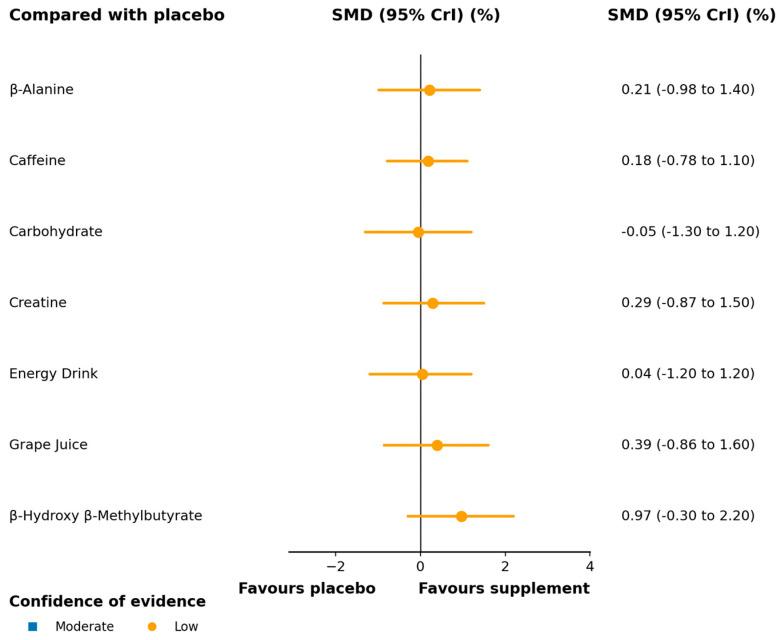
Forest plot of network effect sizes between nutritional supplements and placebo for lower limb mean power. Certainty of evidence is visually represented in the forest map, with the color indicating confidence level. The complete CINeMA assessments are shown in Supplementary Material S9. SMD, standardized mean difference; CrI, credible interval.

Seven trials (*n* = 201) informed the mean lower limb power (analyzed as SMD). Network geometry is presented in Figure 7. No intervention differed from placebo with credible intervals excluding the null (Figure 8). Based on the descriptive SUCRA values, the interventions with the highest relative rankings were HMB, grape juice, and creatine. Because the corresponding subnetwork lacked closed loops, formal local inconsistency testing was not feasible; findings should be interpreted cautiously. Pairwise estimates and SUCRA are provided in Supplementary Tables S7.3 and S8.3; CINeMA certainty was low (imprecision; Supplementary Table S9.3).

### 3.4. Sensitivity Analyses

These subgroup analyses are considered exploratory and hypothesis-generating, given the limited number of trials in each subset.

In the sensitivity analysis excluding high-risk-of-bias trials, the effect estimate for β-alanine on vertical jump was slightly attenuated (mean difference 4.1 cm, 95% CrI 0.8–7.5) compared to the primary analysis (4.6 cm), while β-alanine remained the top-ranked supplement (SUCRA 0.90). Similarly, the expanded network incorporating comparable testing protocols (primary: badminton; secondary: basketball and football) studies yielded comparable results (β-alanine MD 4.4 cm), confirming the robustness of our primary findings. (Supplementary Material S10)

### 3.5. Meta-Regression

Network meta-regression did not identify competitive level or age group as significant effect modifiers for the primary outcomes (Supplementary Material S11), suggesting no systematic modification of supplement effects by these characteristics.

### 3.6. Subgroup Analyses

Competitive level. Among elite athletes, creatine significantly improved vertical jump height (MD 3.7; 95% CrI 0.14–7.45) and β-alanine increased lower limb peak power (SMD 1.33; 0.42–2.24). Among non-elite athletes, β-alanine significantly improved both vertical jump (MD 5.46; 0.75–10.02) and lower limb peak power (SMD 1.79; 0.56–3.01) (Supplementary Material S12).

Age. Using the FIVB age stratification, youth athletes exhibited a significant improvement in vertical jump with creatine (MD 3.53; 95% CrI 0.69 to 6.57); note that negative values denote improvement under the subgroup’s coding convention. Among adults, caffeine significantly increased vertical jump (MD 2.30; 0.27 to 4.32), and BCAA was associated with greater handgrip strength (MD 10.24; 7.22 to 13.28) (Supplementary Material S13).

## 4. Discussion

### 4.1. Principal Findings

Our study represents the first NMA specifically focused on volleyball athletes synthesizing the effects of 35 randomized controlled trials across different supplements for explosive lower limb performance and safety. Overall, β-alanine achieved the most favorable ranking and effect magnitude for vertical jump and lower limb peak power, followed by creatine and caffeine. On CINeMA, certainty ranged from low to moderate, with downgrades primarily for imprecision. While SUCRA values provide useful probabilistic rankings, it is crucial to emphasize that a higher rank does not necessarily indicate statistically significant or clinically meaningful differences between interventions.

From an applied standpoint, β-alanine and creatine could be utilized in training phases (e.g., 3.2–6.4 g/day for a 4–10 week loading protocol) to build explosive lower limb capacity, while caffeine (3–6 mg/kg) can be reserved for acute pre-match intake to yield immediate performance benefits.

### 4.2. General Interpretation of the Results

#### 4.2.1. Vertical Jump

Volleyball is characterized by high-intensity, intermittent, and explosive movements, among which vertical jumping is particularly critical. Greater vertical jump height provides a direct competitive advantage by allowing athletes to spike the ball from a higher point of contact and block a greater vertical area, thereby enhancing both offensive efficiency and defensive effectiveness [65]. These actions place considerable demands on the recruitment of type II muscle fibers in the lower limbs and on intramuscular buffering capacity.

Carnosine, which is highly concentrated in fast-twitch fibers, serve as a key intracellular pH buffer [66,67]. Supplementation with its precursor, β-alanine (BA) [68], increases intramuscular carnosine content and thereby can enhance explosive performance [69,70]. In our Bayesian NMA, BA outperformed other interventions versus placebo for vertical jump height (MD 4.6; 95% CrI 1.2–7.8; SUCRA 80.2%). These results accord with two recent network meta-analyses that reported meaningful improvements in vertical jump following β-alanine (BA) supplementation in footballers and in systematically trained athletes [71,72]. The pattern is physiologically coherent: repeated sprints and jumps in sport accelerate intramuscular H^+^ accumulation, which impairs phosphocreatine (PCr) resynthesis and glycolytic flux, leading to metabolic acidosis. By increasing intramuscular carnosine, BA enhances H^+^ buffering capacity, attenuates the decline in pH, delays the onset of fatigue, and thereby sustains jump performance [16].

We additionally observed significant benefits for creatine (MD 3.7; SUCRA 71.2%) and caffeine (MD 2.1; SUCRA 51.0%) with moderate certainty. The ergogenic effect of creatine on jump performance is well-established [73] and is plausibly mediated by enhanced ATP-PCr availability, improved rate of force development (RFD), and greater neural drive [73,74]. Caffeine’s benefits likely reflect A1/A2A adenosine receptor antagonism (reducing perceived fatigue), Na^+^–K^+^ pump activation and may enhance anaerobic capacity [75,76,77]. The network meta-analysis by Deng et al. reported that β-alanine outperformed creatine and caffeine for jump performance [72], in line with the present findings. By contrast, a prior narrative review in volleyball deemed caffeine the most effective supplement [21]; notably, that synthesis did not include trials of β-alanine. Our quantitative NMA, incorporating both direct and indirect comparisons across a broader experimental corpus, indicates that β-alanine may confer the largest gains in jump height, with creatine and caffeine remaining reliable alternatives—thereby refining and extending the earlier narrative conclusions.

By contrast, HMB (MD 5.96; 95% CrI −2.20 to 13.96) and BCAAs (MD 3.21; −0.43 to 7.07) exhibited SUCRA values >50%, implying potential; CrI is 0—the effect is uncertain and was supported by low confidence of evidence. Mechanistically, both may be more effective in less-trained or catabolic states—facilitating recovery and attenuating performance decrements—rather than producing large gains in maximal explosive performance in already trained athletes [78,79]. For HMB, benefits are frequently restricted to untrained individuals or those under high catabolic stress, with mixed findings in elite cohorts [80]; the training status of our included samples is all elite athletes, which likely limits its marginal utility. For BCAAs, benefits often require longer-term supplementation in conjunction with muscle-damaging exercise to manifest [81,82]; several included trials used short durations and/or suboptimal dosing. Short-term supplementation cannot affect the effect of vertical jump; one study has found that one-week high-dose protocols (20 g·day^−1^) have failed to improve subsequent jump performance [83].

Within the CINeMA framework, certainty for the vertical jump outcome was moderate overall, with downgrades primarily for imprecision (limited sample sizes and wide credible intervals) and heterogeneity across different doses, intervention duration, and testing protocols (e.g., regarding countermovement jump vs. squat jump, CMJ is more dependent on the extensor-tendon complex; SJ is reliant on the maximum strength and power of the femoral extensor group and hip extensor muscles [84], and these will cause different effect sizes). Taken together, β-alanine showed the most consistent improvement in vertical jump performance in our analysis, whereas creatine and caffeine represent evidence-based alternatives that may be deployed according to training phase or acute pre-competition needs. Current rankings for HMB and BCAA should be regarded as hypothesis-generating; rigorously designed head-to-head RCTs with unified dosing, duration, and testing protocols are warranted to delineate their efficacy more definitively.

#### 4.2.2. Lower Limb Peak Power

The explosive strength of the lower limbs is crucial for volleyball performance. High levels of lower limb peak power allow athletes to generate substantial force in a short time, resulting in faster jump initiation, improved on-court agility, and quicker transitions between attack and defense [85].

Neuromuscular coordination plays a fundamental role in the development of lower limb peak power [86]. In our network meta-analysis, only β-alanine showed a statistically significant and credible improvement in this outcome (SMD 1.10; 95% CrI 0.21–2.00; SUCRA 91.8%). The point estimate suggests a moderate-to-large effect size, though the wide credible intervals reflect some imprecision. This finding is physiologically plausible: by increasing intramuscular carnosine, β-alanine enhances H^+^ buffering and may improve sarcoplasmic reticulum Ca^2+^ release and myofilament Ca^2+^ sensitivity, thereby optimizing contraction kinetics during high-intensity efforts [87]; Similar mechanisms have been observed in other explosive disciplines such as elite boxing [88]. Although point estimates for HMB (SMD 0.67), caffeine (SMD 0.39), and creatine (SMD 0.24) also favored supplementation—and all ranked above the 50th percentile by SUCRA—their 95% CrIs included zero, indicating uncertain evidence. This uncertainty may stem from the limited number of available studies, heterogeneity in power assessment methods (e.g., Wingate vs. RAST), and variations in dosing and intervention duration. Using the CINeMA framework, the certainty of evidence for this outcome was rated as low to moderate, primarily downgraded due to imprecision.

Based on current evidence, β-alanine may be considered a first-choice supplement for enhancing lower-limb peak power in volleyball athletes. In contrast, HMB, creatine, and caffeine, while promising, require further validation through adequately powered, head-to-head RCTs using standardized protocols.

#### 4.2.3. Lower Limb Mean Power

Lower limb mean power, defined as the average mechanical work output per unit time, reflects the integrated capacity of the lower extremities to generate force across repeated movements [89]. In volleyball, where players perform successive jumps during rallies and blocks, mean power not only affects single-jump height but also underpins the ability to maintain explosive performance over multiple efforts. The capacity to sustain power output with limited decline is particularly vital in prolonged matches, potentially lasting five sets. Thus, improvements in lower limb mean power may translate into meaningful competitive advantages during match play [90].

Mechanistically, β-alanine has been proposed to support sustained power production via enhanced intramuscular buffering and delayed fatigue [16]. Although this suggests a potential role in maintaining mean power, our analysis identified no studies directly examining β-alanine in this context, and evidence for other interventions was similarly limited. None of the supplements evaluated showed a statistically significant effect, as all 95% credible intervals included the null value.

Nevertheless, SUCRA—which should be interpreted as relative rather than absolute measures of efficacy—indicated that HMB and grape juice were comparatively favorable among the interventions studied. HMB may help maintain mean power by attenuating exercise-induced muscle damage, stabilizing sarcolemmal integrity, reducing inflammatory responses, and promoting recovery [91], thereby supporting performance over repeated high-intensity sequences. Grape juice, ranked second, may improve muscle perfusion and oxygenation via enhanced endothelial nitric oxide synthase (eNOS) activity and nitric oxide bioavailability [92], potentially slowing the decline in power over time.

However, these apparent benefits were modest and inconsistent across studies, aligning more closely with indirect support for power maintenance than with direct ergogenic enhancement. Similarly, although creatine is known to accelerate phosphocreatine resynthesis and may help stabilize power output during intermittent efforts, its effect is likely modulated by test protocol characteristics and the dominant energy systems involved [74]. While some prior reviews have reported creatine-related improvements in mean power [93,94], and caffeine has also been linked to trivial but consistent gains [95], the present analysis did not confirm these effects—a discrepancy that may be attributable to limited study availability and inadequate statistical power.

In summary, based on current low evidence, no dietary supplement can be confidently recommended for the specific purpose of enhancing lower limb mean power in volleyball players. Putative benefits of HMB, grape juice, or creatine remain speculative and warrant further investigation. Future studies should be prospectively designed with sufficient power, align with the energetic demands of volleyball-specific efforts, and evaluate supplementation within ecologically valid training contexts.

### 4.3. Subgroup Analysis

Supplement efficacy appears to be moderated by training background, age, sport-specific demands, and nutritional baseline [96]. We explored whether supplement effects varied by competitive level and age. These analyses are exploratory and should be interpreted with caution, given limited study numbers and potential confounding with dosing, exposure duration, and test modality.

Competitive level. Signals suggested that creatine may yield larger gains in brief explosive tasks (e.g., vertical jump) among elite athletes—consistent with greater ATP-PCr turnover and the ability of well-trained competitors to translate small physiological advantages into performance-relevant outputs during short efforts [97]; operating closer to physiological ceilings, elite athletes can translate subtle physiological advantages into performance-relevant improvements during brief explosive efforts [98]. By contrast, the effects of β-alanine appeared more pronounced in non-elite athletes in our data, which is plausible if lower baseline carnosine allows a larger absolute increase with supplementation [16,99]. However, credible intervals were wide, and subgroup differences were not supported by robust interaction evidence; thus, these patterns are hypothesis-generating rather than confirmatory.

Age. Some evidence suggests that responsiveness to supplements may differ across youth vs. adult athletes [100]. In youth athletes, muscle creatine stores tend to be lower and neuromuscular plasticity higher; consequently, creatine may more readily augment muscle accretion, neural excitability, and vertical jump in adolescents [101]. By contrast, adults—having a higher baseline creatine—often exhibit smaller marginal returns to creatine, while demonstrating greater acute responsiveness to caffeine (potentially via more influential adenosine-receptor signaling) and thus relying more on immediate-effect agents for short-term gains [102].

Collectively, while mechanistic reasoning offers a coherent narrative (ATP-PCr for creatine in brief explosive tasks; H^+^ buffering for β-alanine with larger absolute changes when baseline carnosine is lower; central arousal for caffeine), our subgroup findings should be viewed as exploratory, because our analyses by age and competitive level included few trials per subgroup; thus, these findings should be viewed with caution. Larger, stratified trials are needed to confirm whether supplement effects truly differ by age or experience. Future studies should prespecify subgroup hypotheses, use interaction-focused models (e.g., Bayesian meta-regression), standardize test modality and dosing, and report posterior probabilities of interaction to distinguish true effect modification from confounding.

### 4.4. Practical Implications

Based on our Bayesian synthesis, β-alanine shows the most credible improvement in vertical jump and lower limb peak power (overall low-to-moderate certainty), with creatine and caffeine as evidence-based alternatives.

Given the repeated, high-intensity, intermittent demands of volleyball [2], a 4–6-week β-alanine regimen improved vertical jump by ~4 cm on average in our analysis, which could meaningfully raise spike and block height and thus confer a competitive advantage. Caffeine may acutely increase vertical jump by ~2 cm; while the magnitude is modest, it can still be contextually valuable and is operationally flexible for pre-event use. These observations can inform cost–benefit decisions for practitioners: for example, periodizing 3.2–6.4 g/day β-alanine for 4–10 weeks or 5 g (or 0.33 g/kg)/day creatine for more than 1 week during training phases to maximize adaptations in lower limb peak power and vertical jump performance, as well as deploying caffeine from 3 to 6 mg/kg, taken pre-match to optimize competition day output and deploying the pre-match caffeine with tailored timing; however, using lower doses or different timing could yield smaller gains. It should be emphasized that statistical significance does not equate to practical significance; effect sizes must be interpreted against sport-specific thresholds and competitive context.

Safety and compliance. At recommended doses, most supplements are well tolerated. β-Alanine may provoke transient paresthesia [26], and caffeine warrants attention to individual susceptibility to neurostimulatory adverse effects (e.g., anxiety and tremors) [44,48,52]. A small rise in serum creatinine and hepatic enzymes (AST and ALT) was observed during creatine use [33]. Beetroot juice intake caused mild gastrointestinal discomfort [34]. Athletes should preferentially select third-party-certified products (contamination-free), follow evidence-based dosing, and consult sports-medicine professionals—particularly in anti-doping-tested environments [9].

### 4.5. Limitations and Future Directions

This review is, to our knowledge, the first NMA of nutritional supplements with certainty appraised via CINeMA. Several limitations, however, warrant caution. First, the included trials differed substantially in competitive level, baseline fitness, dosage, and intervention duration, introducing clinical and methodological heterogeneity that may bias effect estimates. Observed heterogeneity may partly reflect differences in dose and supplementation duration. Incorporating age and competitive level in meta-regression reduced between-study heterogeneity (I^2^), suggesting partial explanatory power; however, because the number of trials was insufficient, our prespecified subgroup or meta-regression analyses on dose and duration could not be undertaken. Consequently, residual (unexplained) heterogeneity remains, and we interpreted the findings cautiously, downgrading certainty for inconsistency and imprecision in CINeMA. Although formal testing did not detect significant publication bias for the primary outcomes, the possibility of unpublished null results remains a concern that could potentially inflate the apparent efficacy of interventions. Additionally, incomplete outcome reporting in some included trials may have limited our ability to fully assess treatment effects across all relevant domains.

Second, the treatment network contained few direct head-to-head comparisons; consequently, many estimates relied on indirect evidence, which may reduce inferential stability and increase susceptibility to violation of transitivity assumptions [103]. Although some supplements (e.g., BCAA and HMB) showed favorable rankings, SUCRA reflects relative ordering rather than magnitude or certainty; the corresponding effects were small and/or imprecise, and certainty ranged from low to very low.

Third, most trials in our study lasted only a few weeks, so our findings primarily reflect short-term use; the long-term (multi-month) effects and safety of continuous supplementation in volleyball players remain unknown and warrant investigation.

Finally, our synthesis focused on single-agent effects and did not test potential complementarity among agents; for instance, creatine + β-alanine could theoretically couple phosphagen support with intracellular buffering [104]. We therefore recommend multi-arm or factorial RCT that directly compares combined versus single supplementation strategies, incorporate acute and where feasible, future research should consider pooling individual participant data (IPD) from trials to allow more nuanced subgroup and moderator analyses (age, sex, competitive level, habitual caffeine intake, baseline dietary creatine/β-alanine/nitrate/polyphenols), and incorporate mechanistic endpoints (e.g., ^31^P-MRS PCr recovery, muscle carnosine via HR-MRS together with ecologically valid volleyball outcomes (spike/block height, serve velocity, and match-play metrics) and systematic safety monitoring (including blinding integrity tests for caffeine)). Establishing standardized outcome reporting protocols and fostering international collaboration in volleyball supplement research would enhance comparability and impact. These steps will strengthen transitivity, reduce residual heterogeneity, and improve the external validity and interpretability of future evidence.

## 5. Conclusions

Among volleyball athletes, β-alanine appears to be the most promising supplement for enhancing explosive lower limb performance, specifically vertical jump height and lower limb peak power. Creatine and caffeine may also be effective for improving jump height. Although these interventions possess a good safety profile, the current conclusions are limited by low-to-moderate certainty evidence and must therefore be interpreted with caution. Consequently, larger, more rigorous randomized controlled trials with standardized measures are needed to verify and build upon these results. Furthermore, future studies should prioritize direct comparisons of the leading supplements and investigate their efficacy across diverse athletic populations.

## Figures and Tables

**Table 1:** Study characteristics.

Study	Study Design	Participants Level	Simple Size(N)	Age (Mean ± Sd), Years	Height (Mean ± Sd), Years	Weight (Mean ± Sd), Years	Sex	Study Period	Ingestion Time	Intervention	Comparator(s)	Performance Test	Side Effects
Silvestre et al., 2019 [26]/Brazil	RCT	Elite (professional)	BA:6 PL:5	19 ± 0.9 20 ± 0.6	190 ± 10.0 180 ± 10.0	76.7 ± 6.1 79 ± 3.6	Male	8 weeks	30 min before test	Beta-alanine supplement capsules 6.4 g/day	Placebo (maltodextrin supplement capsules)	VJ	small occurrences of paresthesia
Qanbar et al., 2024 [27]/Iran	RCT	Elite	BA:11 PL:11	24.45 ± 1.36 24.81 ± 1.32	1.85 ± 0.09 1.84 ± 0.07	66.97 ± 9.03 79.63 ± 9.77	Male	4 weeks	daily	Beta-alanine supplement capsules 6.4 g/day	Placebo	VJ, PP	NA
Guo and Wang., 2024 [28]/Korea	RCT	Non-elite (college)	BA:11 PL:11	24.6 ± 2.5 23.8 ± 2.7	182.6 ± 5.5 181.2 ± 6.7	81.5 ± 4.1 79.8 ± 6.9	Male	10 weeks	daily	Beta-alanine supplement capsules 4.8 g/day	Placebo (polydextrose capsules)	VJ, PP, MP	NA
Faiq et al., 2023 [29]/Iraq	RCT	Non-elite (club)	BCAA:8 PL:7	18.5 ± 0.29	NA	NA	NA	8 weeks	daily	BCAA supplementation with strength exercises	Placebo (control group using regular exercises)	VJ	NA
Vega-Sanchez et al., 2020 [30]/Spain	RCT	Elite (professional)	BCAA:6 PL:6	23.8 ± 2.2 25.3 ± 5.1	190.8 ± 13.0 185.7 ± 14.0	84.5 ± 15.1 84.9 ± 13.9	Male	1 week	before breakfast	BCAA supplement 7 g mixed in 500 mL of water	Placebo (500 mL of a watermelon-flavored beverage)	VJ	NA
Santi et al., 2020 [31]/Brazil	RCT	Elite (well-trained)	CRT:7 CHO:7	18 ± 0.3 19 ± 0.4	1.83 ± 0.04 1.80 ± 0.06	72.2 ± 10.7 81 ± 5.7	NA	11 days	daily	0.3 g/kg/day of creatine associated with 1.2 g/kg/day of carbohydrate	Placebo (1.5 g/kg/day maltodextrin)	VJ, MP	NA
Lamontagne-Lacasse et al., 2011 [32]/Canada	RCT	Elite	12	22 ± 1.5	84 ± 8	190 ± 7	Male	28 days	daily	20 g of dextrose, 10 g of sucrose, 300 mL of water, and artificial flavor with 5 g of creatine	Placebo (20 g of dextrose, 10 g of sucrose, 300 mL of water, and artificial flavor)	VJ	NA
Kubota et al., 2003 [33]/Japan	RCT	Non-elite (collegiate)	CRT:11 PL:10	23.4 ± 4.7 22.8 ± 4.3	185.6 ± 5.7 184.4 ± 62	78.2 ± 2.7 78.5 ± 8.6	Male	6 days	4 times a day	5 g CRT and 5 g sports drink dissolved in 100 mL of water (20 g/day)	Placebo (5 g sports drink dissolved in 100 mL of water 20 g/day)	VJ, MP	Serum creatine, AST, and ALT were slightly elevated
Hemmatinafar et al., 2023 [34]/Iran	RCT	Non-elite (semi-professional)	14	26.00 ± 3.00	174.08 ± 3.94	67.75 ± 5.14	Female	32 days	daily	50 mL beetroot juice supplementation (total 400 mL over 2 days)	Placebo (matched for calories and appearance, but with negligible nitrate)	VJ	2 digestive disorders
Toohey et al., 2020 [35]/USA	RCT	elite	23	19.6 ± 1.0	170.6 ± 6.8	67.5 ± 7.4	Female	10 weeks	daily, post-workout	Probiotic Bacillus subtilis DE111 (5 billion CFU/day)	Placebo	VJ, PP	NA
Setaro et al., 2014 [36]/Brazil	RCT	Elite (professional)	MG:12 PL:13	17.42 ± 1.56 17.85 ± 0.99	191.4 ± 9.0 195.7 ± 8.9	83 ± 9.5 82.9 ± 7.8	Male	4 weeks	daily	Magnesium oxide capsules, 350 mg/day	Placebo (maltodextrin capsules, 500 mg/day)	VJ, PP	NA
Portal et al., 2011 [37]/Israel	RCT	Elite	HMB:14 PL:14	16.1 ± 1.3 16.2 ± 1.3	185.0 ± 9.6 181.9 ± 9.0	72.3 ± 10.3 69.9 ± 11.3	Male and Female	7 weeks	morning before training	HMB pills 3 g/day	Placebo pills 3 g/day	VJ, PP, MP	NA
Sánchez-Gómez et al., 2022 [38]/Spain	RCT	Elite (national)	8	33.5 ± 8.95	NA	HMB:80.3 ± 10.7 PL:78.8 ± 8.4	Male and Female	4 weeks	60 min before test	HMB capsules 3 g/day	Placebo (sucrose capsules)	VJ, PP, MP	NA
Nemati et al., 2023 [39]/Iran + Japna	RCT	Non-elite (collegiate)	15	20.80 ± 1.00	181.40 ± 4.30	70.22 ± 6.92	Male	3 weeks	60 min before test	Caffeine capsules 3 mg/kg and 6 mg/kg	Placebo (starch capsule)	VJ	NA
Pfeifer et al., 2017 [40]/USA	RCT	Non-elite (collegiate)	8	20 ± 1.15	174.8 ± 4.69	72.1 ± 9.94	Female	3 weeks	immediately before test	CHO (1.34 g/kg) and CAF (1.39 mg/kg)	Placebo (non-nutritive gel)	VJ,	blood glucose rise
Siquier-Coll et al., 2024 [41]/Spain	RCT	Non-elite (semi-professional)	8	21 ± 2.31	1.63 ± 8	66.67 ± 4.74	Female	2 weeks	60 min before test	Caffeine anhydrous powder mixed with maltodextrin-based beverage 5 mg/kg	Placebo (maltodextrin-based beverage)	VJ	NA
Zbinden-Foncea et al., 2018 [42]/Chile	RCT	Elite	10	18.80 ± 2.00	1.93 ± 0.04	85.22 ± 10.11	Male	3 weeks	60 min before test	Caffeine capsule 5 mg/kg	Placebo (dextrose capsule)	VJ, PP	NA
Fernández-Campos et al., 2015 [43]/USA + Costa Rica	RCT	Elite (professional)	19	22.3 ± 4.9	171.8 ± 9.4	65.2 ± 10.1	Female	3 weeks	30 min before test	Energy drink 6 mL/kg	Placebo (carbonated water)	VJ, PP, MP	NA
Del Coso et al., 2014 [44]/Spain	RCT	Non-elite (college)	15	21.8 ± 6.9	180 ± 8	79.6 ± 11.0	Male	2 weeks	60 min before test	Caffeine 3 mg/kg	Energy drink	VJ, MP	Insomnia
Filip-Stachnik et al., 2022 [45]/Spian + Poland	RCT	Elite (high-performance)	12	20 ± 2	178 ± 6	69.1 ± 2.3	Female	1 week	15 min before test	Caffeinated chewing gum (6.4 mg/kg)	Placebo (non-caffeinated gum)	VJ	NA
Filip-Stachnik et al., 2022 [46]/Poland	RCT	Non-elite (semi-professional)	14	26 ± 3	171 ± 5	62.6 ± 5.6	Female	2 weeks	60 min before test	Caffeinated capsule 6 mg/kg	Placebo (starch capsule)	VJ	NA
Kaszuba et al., 2022 [47]/Poland	RCT	Elite (high-performance)	12	23 ± 3	188 ± 8	85.9 ± 11.2	Male and Female	2 weeks	15 min before test	Caffeinated chewing gum (3.2 mg/kg)	Placebo (non-caffeinated gum)	VJ	NA
Pérez-López et al., 2015 [48]/Spain	RCT	Elite	13	25.2 ± 4.8	174 ± 9	64.4 ± 7.6	Female	2 weeks	60 min before test	Caffeine 3 mg/kg	Energy drink	VJ, PP	Nervousness, activeness
Wang et al., 2025 [49]/China	RCT	Non-elite (college)	CAF: 12 RHO: 12 CAF + RHO: 12 PL: 12	21 ± 1 20 ± 2 20 ± 1 20 ± 1	185 ± 5 183 ± 4 183 ± 4 183 ± 5	78 ± 5 79 ± 5 79 ± 5 78 ± 5	Male	4 weeks	30 min before test	CAF: caffeine capsule 3 mg/kg RHO: RHO extract (2.4 g per day)	Placebo (capsules)	VJ	NA
Martins et al., 2020 [50]/Brazil	RCT	Elite (high-performance)	12	16.5± 0.6	186.6 ± 8.4	77.5 ± 8.4	Male	2 weeks	daily	Grape juice (purple, Vitis labrusca Bordeaux, 400 mL/day)	Placebo beverage (maltodextrin, matched calories and carbohydrates, no polyphenols)	MP	NA
Telyari and Ebrahimi, 2022 [51]/Iran	RCT	Non-elite (semi-professional)	12	21.5 ± 2.02	184.00 ± 3.25	79.33 ± 4.71	Male	1 week	20 s before test	Caffeinated mouthwash (200 mg caffeine dissolved in 25 mL water)	Placebo (water with flour)	VJ,	NA
Lee et al., 2014 [52]/China	RCT	Non-elite (collegiate)	11	21.3 ± 1.2	164.2 ± 5.7	58.6 ± 7.3	Female	1 week	CAF: 60 min before test; CHO: immediately before test	CAF: Caffeine capsules (6 mg/kg) CHO: Carbohydrate solution (0.8 g/kg dextrose)	Placebo (cellulose capsules); Placebo (low-calorie artificial sweetener drink)	PP, MP	anxiety, tremor, diarrhea, headache
Elbattawy et al., 2015 [53]/Egypt	RCT	Non-elite (club)	BCAA: 6; CRT: 6; PLA: 6	20.5 ± 1.5	187.31 ± 5.40	79.47 ± 9.21	Male	3 weeks	60 min before test	BCAA group: 4 g, taken 3× per day (with 250 mL apple juice); Creatine group: Creatine 0.33 g/kg + Panax ginseng 1.5 g taken 3× per day (with 250 mL apple juice)	Placebo (250 mL apple juice, 3× per day)	VJ	NA
Burke et al., 2021 [54]/USA	RCT	Non-elite (college)	11	19.7 ± 0.9	166.4 ± 10.2	67.7 ± 9.4	Female	1 week	60 min before test	Caffeine anhydrous at 6 mg/kg	Placebo capsule	VJ, PP	NA
Hashem et al., 2024 [55]/Iraq	RCT	Non-elite (college)	24	16.5 ± 0.29	NA	NA	Male	8 weeks	30 min before test	Creatine 5 g	Placebo	VJ	NA
Zhu et al., 2025 [56]/China	RCT	Elite (high-performance)	CAF-3: 8; CAF-6: 8; CAF-3→6: 8; Placebo: 8	20.5 ± 1.1; 20.6 ± 1.3; 20.7 ± 1.2; 20.4 ± 1.2	184.4 ± 3.9 cm; 183.7 ± 4.6 cm; 185.2 ± 3.3 cm; 184.8 ± 4.1 cm	84.2 ± 4.1 kg; 82.7 ± 5.6 kg; 83.3 ± 4.5 kg; 84.7 ± 3.9 kg	Male	4 weeks	45 min before test	Caffeine capsules (3 mg/kg; 6 mg/kg; 3–6 mg/kg)	Placebo capsules	VJ, PP, MP	NA
López-León et al., 2025 [57]/Spain + Brazil	RCT	Elite (high-performance)	12	22.9 ± 3.6	NA	NA	Female	2 weeks	2.5 h before testing	Beetroot juice supplementation 12.8 mmol NO_3_^−^ per dose	Placebo beverage (nitrate-depleted beetroot juice, identical in flavor/appearance)	VJ	NA
Vinu., 2018 [58]/India	RCT	Non-elite (college)	PLA: 15; PRT: 15; PL: 15	20 ± 1.15	168.5 ± 3.8	67.5 ± 4.3	Male	12 weeks	daily	Plyometric jump training program combined with protein supplementation	Placebo (Plyometric training without any supplement) Placebo (No plyometric training, no supplement)	VJ	NA
Campbell et al., 2016 [59]/USA	RCT	Non-elite (college)	ED: 10; PLA: 9	22.4 ± 3.2	168.7	69.0 ± 12.7	Male and Female	1 week	30 min before test	Energy drink (containing caffeine 2.4 mg/kg)	Placebo beverage (37 mL, non-caloric, similar taste and volume)	VJ	NA
Norozi et al., 2025 [60]/Iran	RCT	Non-elite (semi-professional)	LC: 10; PLA: 9	18.1 ± 2.8; 17.7 ± 2.9	167.4 ± 3.89; 166.3 ± 3.74	61.85 ± 14.21; 58.80 ± 10.93	Female	8 weeks	60 min before training	2 g/day L-citrulline malate (powder, dissolved in 200 mL water, Ktowa Hakko Bio Co., Japan)	Placebo (2 g/day cellulose)	VJ	NA

Abbreviations: PLA, Placebo; BA, β-Alanine; BCAAs, Branched-chain amino acids; CHO, Carbohydrate; CRT, Creatine; BRT, Beetroot juice; PBC, Probiotics; MG, Magnesium; HMB, β-Hydroxy-β-Methylbutyrate; CAF, Caffeine; ED, Energy drink; PRT, Protein; GPJ, Grape juice; LC, L-Citrulline; VJ, Vertical jump; PP, Lower limb peak power; MP, Lower limb mean power; AST: Aspartate aminotransferase; ALT: Alanine aminotransferase; NA, not available.

## Data Availability

All aggregate data generated or analyzed during this study are included in this published article (and its Supplementary Materials Information File). Data are available to qualified investigators on reasonable request.

## Supplementary Materials

The following supporting information can be downloaded at: https://www.mdpi.com/article/10.3390/nu17233702/s1, Table S1: Search strategy of PubMed; Table S2: Search strategy of Embase; Table S3: Search strategy of Cochrane Central Register of Controlled Trials; Table S4: Search strategy of Web of Science; Figure S2: Overall risk of bias presented as percentage of each risk of bias item across all included studies; Figure S3.1: Gelman-Rubin Plots of Vertical Jump; Figure S3.2: Gelman-Rubin Plots of Lower limb Peak Power; Figure S3.3: Gelman-Rubin Plots of Lower limb Mean Power; Figure S4.1: Convergence Diagnostics: Trace and Density Plots of Vertical Jump; Figure S4.2: Convergence Diagnostics: Trace and Density Plots of Lower limb Peak Power; Figure S4.3: Convergence Diagnostics: Trace and Density Plots of Lower limb Mean Power; Figure S5.1: Vertical Jump consistency; Figure S5.2: Lower limb Peak Power consistency; Table S6.1: Network heterogeneity assessment results of Vertical Jump; Table S6.2: Network heterogeneity assessment results of Lower limb Peak Power; Table S6.3: Network heterogeneity assessment results of Lower limb Mean Power; Table S7.1: SUCRA of the effects of different dietary supplements on Vertical Jump; Table S7.2: SUCRA of the effects of different dietary supplements on Lower limb Peak Power; Table S7.3: SUCRA of the effects of different dietary supplements on Lower limb Mean Power; Table S8.1: Vertical Jump; Table S8.2: Lower limb Peak Power; Table S8.3: Lower limb Mean Power; Figure S9.1: Risk of bias contribution by intervention group in Vertical Jump; Figure S9.2: Overall risk of bias by treatment comparison in Vertical Jump; Figure S9.3: Risk of bias contribution by intervention group in Lower limb Peak Power; Figure S9.4: Overall risk of bias by treatment comparison in Lower limb Peak Power; Figure S9.5: Risk of bias contribution by intervention group in Lower limb Mean Power; Figure S9.6: Overall risk of bias by treatment comparison in Lower limb Mean Power; Table S9.1: CINeMA results of Vertical Jump; Table S9.2: CINeMA results of Lower limb Peak Power; Table S9.3: CINeMA results of Lower limb Mean Power; Table S10.1: Baseline characteristics of included studies supplemented in sensitivity analyses [105,106,107,108,109]; Table S10.2: Sensitivity analyses of Vertical Jump outcome; Table S10.3: Sensitivity analyses of Lower limb Peak Power outcome; Table S10.4: Sensitivity analyses of Lower limb Mean Power outcome; Table S11.1: Meta-regression of Vertical Jump; Table S11.2: Meta-regression of Lower limb Peak Power; Table S11.3: Meta-regression of Lower limb Mean Power; Table S12.1: League table of youth volleyball athletes; Table S12.2: League table of adult volleyball athletes; Table S13.1: League table of elite volleyball athletes; Table S13.2: League table of non-elite volleyball athletes; Figure S14.1: Vertical Jump funnel plot and Egger test; Figure S14.2: Lower limb Peak Power funnel plot and Egger test; Figure S14.3: Lower limb Mean Power funnel plot.

## Abbreviations

The following abbreviations are used in this manuscript:

A1/A2A	Adenosine Receptor Subtypes A1 and A2A
AST	Aspartate Aminotransferase
ALT	Alanine Aminotransferase
ATP	Adenosine Triphosphate
BA	Beta-Alanine
BCAAs	Branched-Chain Amino Acids
CI	Confidence Interval
CINeMA	Confidence in Network Meta-Analysis
CMJ	Countermovement Jump
CrI	Credible Interval
FIVB	Fédération Internationale de Volleyball
HR-MRS	High-Resolution Magnetic Resonance Spectroscopy
HMB	Beta-Hydroxy-Beta-Methylbutyrate
IPD-NMA	Individual Participant Data Network Meta-Analysis
ISSN	International Society of Sports Nutrition
MD	Mean Difference
MCMC	Markov Chain Monte Carlo
mTOR	Mechanistic Target of Rapamycin
NA	Not Available
NMA	Network Meta-Analysis
NO	Nitric Oxide
PCr	Phosphocreatine
PH	Potential of Hydrogen
PRISMA	Preferred Reporting Items for Systematic Reviews and Meta-Analyses
PROSPERO	International Prospective Register of Systematic Reviews
RCT	Randomized Controlled Trial
RFD	Rate of Force Development
RAST	Running-based Anaerobic Sprint Test
RoB 2.0	Risk of Bias 2.0 Tool
SD	Standard Deviation
SMD	Standardized Mean Difference
SJ	Squat Jump
SUCRA	Surface Under the Cumulative Ranking Curve
WADA	World Anti-Doping Agency
^31^P-MRS PCr recovery	Phosphorus-31 Magnetic Resonance Spectroscopy—Phosphocreatine Recovery

## References

[1] Garcia-de-Alcaraz A., Ramirez-Campillo R., Rivera-Rodriguez M., Romero-Moraleda B. (2020). Analysis of jump load during a volleyball season in terms of player role. J. Sci. Med. Sport.

[2] Sheppard J.M., Gabbett T., Taylor K.L., Dorman J., Lebedew A.J., Borgeaud R. (2007). Development of a repeated-effort test for elite men’s volleyball. Int. J. Sports Physiol. Perform..

[3] VanHeest J.L. (2003). Energy demands in the sport of volleyball. Handbook of Sports Medicine and Science: Volleyball.

[4] Andreoli A., Melchiorri G., Brozzi M., Di Marco A., Volpe S.L., Garofano P., Di Daniele N., De Lorenzo A. (2003). Effect of different sports on body cell mass in highly trained athletes. Acta Diabetol..

[5] Fuchs P.X., Mitteregger J., Hoelbling D., Menzel H.-J.K., Bell J.W., von Duvillard S.P., Wagner H. (2021). Relationship between General Jump Types and Spike Jump Performance in Elite Female and Male Volleyball Players. Appl. Sci..

[6] Giatsis G., Drikos S., Lola A. (2022). Analysis of match report indicators in men’s volleyball Olympics and world championships (2014–2021) depending on the type of final score. Int. J. Sports Sci. Coach..

[7] Pawlik D., Mroczek D. (2023). Influence of jump height on the game efficiency in elite volleyball players. Sci. Rep..

[8] Lidor R., Ziv G. (2010). Physical and physiological attributes of female volleyball players—A review. J. Strength Cond. Res..

[9] Maughan R.J., Burke L.M., Dvorak J., Larson-Meyer D.E., Peeling P., Phillips S.M., Rawson E.S., Walsh N.P., Garthe I., Geyer H. (2018). IOC Consensus Statement: Dietary Supplements and the High-Performance Athlete. Int. J. Sport Nutr. Exerc. Metab..

[10] Kerksick C.M., Wilborn C.D., Roberts M.D., Smith-Ryan A., Kleiner S.M., Jager R., Collins R., Cooke M., Davis J.N., Galvan E. (2018). ISSN exercise & sports nutrition review update: Research & recommendations. J. Int. Soc. Sports Nutr..

[11] Rawson E.S., Miles M.P., Larson-Meyer D.E. (2018). Dietary Supplements for Health, Adaptation, and Recovery in Athletes. Int. J. Sport Nutr. Exerc. Metab..

[12] Sobiński A., Czerwonka M., Kościuszko Z., Kurza K., Ciraolo S., Podolec J., Agnieszka K.-R., Lesiczka-Fedoryj K., Wojda J., Walczak A. (2025). The Impact of Creatine Supplementation on Physical Performance, Cognitive Functions, and Safety—A Literature Review. Qual. Sport.

[13] Branch J.D. (2003). Effect of creatine supplementation on body composition and performance: A meta-analysis. Int. J. Sport Nutr. Exerc. Metab..

[14] Salinero J.J., Lara B., Del Coso J. (2019). Effects of acute ingestion of caffeine on team sports performance: A systematic review and meta-analysis. Res. Sports Med..

[15] Guest N.S., VanDusseldorp T.A., Nelson M.T., Grgic J., Schoenfeld B.J., Jenkins N.D.M., Arent S.M., Antonio J., Stout J.R., Trexler E.T. (2021). International society of sports nutrition position stand: Caffeine and exercise performance. J. Int. Soc. Sports Nutr..

[16] Saunders B., Elliott-Sale K., Artioli G.G., Swinton P.A., Dolan E., Roschel H., Sale C., Gualano B. (2017). beta-alanine supplementation to improve exercise capacity and performance: A systematic review and meta-analysis. Br. J. Sports Med..

[17] Shalaby M.N., Sakoury M.M., Kholif M.A., Albadaly N.I.A. (2020). The role of Amino Acids in improving immunity and growth factors of Volleyball players. J. Adv. Pharm. Educ. Res..

[18] Jager R., Kerksick C.M., Campbell B.I., Cribb P.J., Wells S.D., Skwiat T.M., Purpura M., Ziegenfuss T.N., Ferrando A.A., Arent S.M. (2017). International Society of Sports Nutrition Position Stand: Protein and exercise. J. Int. Soc. Sports Nutr..

[19] Zapolska J., Witczak K., Manczuk A., Ostrowska L. (2014). Assessment of nutrition, supplementation and body composition parameters on the example of professional volleyball players. Rocz. Panstw. Zakl. Hig..

[20] Silva M., Marcelino R., Lacerda D., Vicente João P. (2016). Match Analysis in Volleyball: A systematic review. Montenegrin J. Sports Sci. Med..

[21] Hernández-Landa R.E., Lazo M., Salado D.D., Sánchez-Almanzar E., Cepeda-Marte J.L., Zare R., Ali Redha A., Clifford T. (2024). Dietary Supplementation Strategies for Improving Training Adaptations, Antioxidant Status and Performance of Volleyball Players: A Systematic Review. J. Sci. Sport Exerc..

[22] Burke L.M. (2017). Practical Issues in Evidence-Based Use of Performance Supplements: Supplement Interactions, Repeated Use and Individual Responses. Sports Med..

[23] Dias S., Caldwell D.M. (2019). Network meta-analysis explained. Archives of Disease in Childhood. Fetal and Neonatal Edition.

[24] Challoumas D., Artemiou A. (2018). Predictors of Attack Performance in High-Level Male Volleyball Players. Int. J. Sports Physiol. Perform..

[25] Fédération Internationale de Volleyball (FIVB) (2024). Sports Regulations 2024.

[26] Silvestre J., Gianoni R., Esteves G., Lambertucci R., Zagatto A., Azevedo P. (2019). Beta-Alanine Supplementation Neither Reduce the Oxidative Stress Nor Improve Physical Performance of Volleyball Athletes. https://www.researchgate.net/publication/335563784_Beta-alanine_Supplementation_Neither_Reduce_the_Oxidative_Stress_nor_Improve_Physical_Performance_of_Volleyball_Athletes.

[27] Qanbar Y.R., Talebi-Garakani E., Safarzade A. (2024). Interleukin-15 Contributes to the Benefits of Βeta-Alanine Supplementation on the Performance of Elite Volleyball Players. Ann. Mil. Health Sci. Res..

[28] Guo W., Wang S. (2024). Physiological and performance adaptations to beta alanine supplementation and short sprint interval training in volleyball players. Sci. Rep..

[29] Faiq D.S., Essa S.N.A., Muhialdeen S.S. (2023). The Effect of Assisted Training Using Supplementation (BCAA) in Developing the Special Strength and Accuracy of Offensive Skills in Volleyball for Youth. Egypt. J. Hosp. Med..

[30] Vega-Sanchez R., Tolentino-Dolores M.C., Cerezo-Rodriguez B., Chehaibar-Besil G., Flores-Quijano M.E. (2020). Erythropoiesis and Red Cell Indices Undergo Adjustments during Pregnancy in Response to Maternal Body Size but not Inflammation. Nutrients.

[31] Santi C.S., Júnior J.F.A., dos Santos A.N., da Silva D.P., Leite R.D. (2020). Effect of creatine supplementation on muscle damage markers and physical performance in volleyball athletes. J. Exerc. Physiol. Online.

[32] Lamontagne-Lacasse M., Nadon R., Goulet E.D. (2011). Effect of creatine supplementation on jumping performance in elite volleyball players. Int. J. Sports Physiol. Perform..

[33] Kubota M., Hiruma E., Sasaki H. (2003). The Effects of creatine supplementation on jumping power and endurance of volleyball players. Adv. Exerc. Sports Physiol..

[34] Hemmatinafar M., Zaremoayedi L., Koushkie Jahromi M., Alvarez-Alvarado S., Wong A., Niknam A., Suzuki K., Imanian B., Bagheri R. (2023). Effect of Beetroot Juice Supplementation on Muscle Soreness and Performance Recovery after Exercise-Induced Muscle Damage in Female Volleyball Players. Nutrients.

[35] Toohey J.C., Townsend J.R., Johnson S.B., Toy A.M., Vantrease W.C., Bender D., Crimi C.C., Stowers K.L., Ruiz M.D., VanDusseldorp T.A. (2020). Effects of Probiotic (*Bacillus subtilis*) Supplementation During Offseason Resistance Training in Female Division I Athletes. J. Strength Cond. Res..

[36] Setaro L., Santos-Silva P.R., Nakano E.Y., Sales M.M., Pereira G.B., Miranda R.A., Colli C. (2014). Magnesium status and its relationship with muscle damage in volleyball players. Magnes. Res..

[37] Portal S., Zadik Z., Rabinowitz J., Pilz-Burstein R., Adler-Portal D., Meckel Y., Cooper D.M., Eliakim A., Nemet D. (2011). The effect of HMB supplementation on body composition, fitness, hormonal and inflammatory mediators in elite adolescent volleyball players: A prospective randomized, double-blind, placebo-controlled study. Eur. J. Appl. Physiol..

[38] Sánchez-Gómez A., Martínez-Aranda L.M., Vicente-Salar N., Martínez-Noguera F.J., Alcaraz P.E. (2022). β-hydroxy-β-methylbutyrate free acid supplementation and exercise-induced muscle damage in elite male volleyball players: A randomized, double-blind, placebo-controlled trial. Nutrients.

[39] Nemati J., Hemmatinafar M., Niknam A., Nikahd M., Zeighami N., Imanian B., Safari K., Jahaniboushehri N., Suzuki K. (2023). Effects of Different Doses of Caffeine Supplementation on Collegiate Male Volleyball Players’ Specific Performance and Skills: A Randomized, Double-Blind, Placebo-Controlled, Crossover Study. Nutrients.

[40] Pfeifer D.R., Arvin K.M., Herschberger C.N., Haynes N.J., Renfrow M.S. (2017). A Low Dose Caffeine and Carbohydrate Supplement does not Improve Athletic Performance during Volleyball Competition. Int. J. Exerc. Sci..

[41] Siquier-Coll J., Delgado-Garcia G., Soto-Mendez F., Linan-Gonzalez A., Garcia R., Gonzalez-Fernandez F.T. (2023). The Effect of Caffeine Supplementation on Female Volleyball Players’ Performance and Wellness during a Regular Training Week. Nutrients.

[42] Zbinden-Foncea H., Rada I., Gomez J., Kokaly M., Stellingwerff T., Deldicque L., Penailillo L. (2018). Effects of Caffeine on Countermovement-Jump Performance Variables in Elite Male Volleyball Players. Int. J. Sports Physiol. Perform..

[43] Fernandez-Campos C., Dengo A.L., Moncada-Jimenez J. (2015). Acute Consumption of an Energy Drink Does Not Improve Physical Performance of Female Volleyball Players. Int. J. Sport Nutr. Exerc. Metab..

[44] Del Coso J., Perez-Lopez A., Abian-Vicen J., Salinero J.J., Lara B., Valades D. (2014). Enhancing physical performance in male volleyball players with a caffeine-containing energy drink. Int. J. Sports Physiol. Perform..

[45] Filip-Stachnik A., Kaszuba M., Dorozynski B., Komarek Z., Gawel D., Del Coso J., Klocek T., Spieszny M., Krzysztofik M. (2022). Acute Effects of Caffeinated Chewing Gum on Volleyball Performance in High-Performance Female Players. J. Human Kinet..

[46] Filip-Stachnik A., Spieszny M., Stanisz L., Krzysztofik M. (2022). Does caffeine ingestion affect the lower-body post-activation performance enhancement in female volleyball players?. BMC Sports Sci. Med. Rehabil..

[47] Kaszuba M., Klocek O., Spieszny M., Filip-Stachnik A. (2022). The Effect of Caffeinated Chewing Gum on Volleyball-Specific Skills and Physical Performance in Volleyball Players. Nutrients.

[48] Perez-Lopez A., Salinero J.J., Abian-Vicen J., Valades D., Lara B., Hernandez C., Areces F., Gonzalez C., Del Coso J. (2015). Caffeinated energy drinks improve volleyball performance in elite female players. Med. Sci. Sports Exerc..

[49] Wang Z., Du H., Li H., Zhao K., Zhao B., Ma Y., Zhang J., Wu K., Jiang W., Liu C. (2025). Effects of the Combined Supplementation of Caffeine and Rhodiola Rosea with Resistance Training on Lower Limb Explosive Power in Male Volleyball Players. Nutrients.

[50] Martins N.C., Dorneles G.P., Blembeel A.S., Marinho J.P., Proenca I.C.T., da Cunha Goulart M.J.V., Moller G.B., Marques E.P., Pochmann D., Salvador M. (2020). Effects of grape juice consumption on oxidative stress and inflammation in male volleyball players: A randomized, double-blind, placebo-controlled clinical trial. Complement. Ther. Med..

[51] Telyari M., Ebrahimi M. (2022). The effect of caffeine mouth rinsing on agility, jump height and service and spike accuracy in male volleyball players. Res. Exerc. Nutr..

[52] Lee C.L., Cheng C.F., Astorino T.A., Lee C.J., Huang H.W., Chang W.D. (2014). Effects of carbohydrate combined with caffeine on repeated sprint cycling and agility performance in female athletes. J. Int. Soc. Sports Nutr..

[53] Ismail A., Elbattawy K. (2015). Effect of Branched-Chain Amino Acids and Ginseng-Creatine supplementation on delayed onset muscle soreness and muscle damage in volleyball players. Assiut J. Sport Sci. Arts.

[54] Burke B.I., Travis S.K., Gentles J.A., Sato K., Lang H.M., Bazyler C.D. (2021). The Effects of Caffeine on Jumping Performance and Maximal Strength in Female Collegiate Athletes. Nutrients.

[55] Hashem E.J., Majeed M.H., Al-Isawi M. (2024). Effect of Nutritional Supplement Accompanying Functional Strength Exercises on the Special Physical Abilities and the Spiking Skill of Volleyball Players. Int. J. Disabil. Sports Health Sci..

[56] Zhu G., Fu K., Xie Y. (2025). Effects of progressive versus consistent dose of caffeine ingestion on volleyball players’ exercise performance adaptations following plyometric jump training. Front. Nutr..

[57] López-León I., Moreno-Lara J., Sánchez-Oliver A.J., Rico-Saborido E., Muñoz-López A., Llerena A.M., Domínguez R. (2025). Does beetroot juice supplementation affect to sport performance in high performance female volleyball players?. Cult. Cienc. Deporte.

[58] Vinu W. (2018). Effect of plyometric training and plyometric training with protein suplementation on explosive power. Int. J. Adv. Educ. Res..

[59] Campbell B.I., Richmond J.L., Dawes J.J. (2016). The Effects of a Commercial, Pre-exercise Energy Drink Supplement on Power, Muscular Endurance, and Repeated Sprint Speed. Int. J. Exerc. Sci..

[60] Norozi M., Hejazi K., Marefati H. (2025). The effect of eight weeks of high-intensity interval training combined with L-citrulline malate supplementation on muscle damage, inflammation and physical fitness factors of volleyball girls. Comp. Exerc. Physiol..

[61] Eskici G., Gunay M., Baltaci A.K., Mogulkoc R. (2017). The effect of different doses of zinc supplementation on serum element and lactate levels in elite volleyball athletes. J. Appl. Biomed..

[62] García Verazaluce J.J., Vargas Corzo M.D.C., Aguilar Cordero M.J., Ocaña Peinado F., Sarmiento Ramírez Á., Guisado Barrilao R. (2015). Effect of phlebodium decumanum and coenzyme Q10 on sports performance in professional volleyball players. Nutr. Hosp..

[63] Mielgo-Ayuso J., Zourdos M.C., Calleja-González J., Urdampilleta A., Ostojic S. (2015). Iron supplementation prevents a decline in iron stores and enhances strength performance in elite female volleyball players during the competitive season. Appl. Physiol. Nutr. Metab..

[64] Kartashev V., Medvedev I., Kachenkova E. (2023). The use of the sports nutrition complex BCAA + PEPTIDECOMPLEXIPH-AGAA in representatives of team sports. Theory Pract. Phys. Cult..

[65] Silva A.F., Clemente F.M., Lima R., Nikolaidis P.T., Rosemann T., Knechtle B. (2019). The Effect of Plyometric Training in Volleyball Players: A Systematic Review. Int. J. Environ. Res. Public Health.

[66] De Brandt J., Burtin C., Pomies P., Vandenabeele F., Verboven K., Aumann J., Blancquaert L., Everaert I., Van Ryckeghem L., Cops J. (2021). Carnosine, oxidative and carbonyl stress, antioxidants, and muscle fiber characteristics of quadriceps muscle of patients with COPD. J. Appl. Physiol. (1985).

[67] Baguet A., Everaert I., Hespel P., Petrovic M., Achten E., Derave W. (2011). A new method for non-invasive estimation of human muscle fiber type composition. PLoS ONE.

[68] Matthews J.J., Artioli G.G., Turner M.D., Sale C. (2019). The Physiological Roles of Carnosine and beta-Alanine in Exercising Human Skeletal Muscle. Med. Sci. Sports Exerc..

[69] Blancquaert L., Everaert I., Missinne M., Baguet A., Stegen S., Volkaert A., Petrovic M., Vervaet C., Achten E., De Maeyer M. (2017). Effects of Histidine and beta-alanine Supplementation on Human Muscle Carnosine Storage. Med. Sci. Sports Exerc..

[70] Harris R.C., Tallon M.J., Dunnett M., Boobis L., Coakley J., Kim H.J., Fallowfield J.L., Hill C.A., Sale C., Wise J.A. (2006). The absorption of orally supplied beta-alanine and its effect on muscle carnosine synthesis in human vastus lateralis. Amino Acids.

[71] Luo H., Tengku Kamalden T.F., Zhu X., Xiang C., Nasharuddin N.A. (2025). Effects of different dietary supplements on athletic performance in soccer players: A systematic review and network meta-analysis. J. Int. Soc. Sports Nutr..

[72] Deng B., Yan R., He T., Lin G., Liu T., Chen W., He J., Li D. (2025). Effects of different dietary supplements combined with conditioning training on muscle strength, jump performance, sprint speed, and muscle mass in athletes: A systematic review and network meta-analysis. Front. Nutr..

[73] Kreider R.B., Kalman D.S., Antonio J., Ziegenfuss T.N., Wildman R., Collins R., Candow D.G., Kleiner S.M., Almada A.L., Lopez H.L. (2017). International Society of Sports Nutrition position stand: Safety and efficacy of creatine supplementation in exercise, sport, and medicine. J. Int. Soc. Sports Nutr..

[74] Butts J., Jacobs B., Silvis M. (2018). Creatine Use in Sports. Sports Health.

[75] Tamilio R.A., Clarke N.D., Duncan M.J., Morris R.O., Tallis J. (2022). How Repeatable Is the Ergogenic Effect of Caffeine? Limited Reproducibility of Acute Caffeine (3 mg.kg^−1^) Ingestion on Muscular Strength, Power, and Muscular Endurance. Nutrients.

[76] Dodd S.L., Herb R.A., Powers S.K. (1993). Caffeine and exercise performance. An update. Sports Med..

[77] Echeverri D., Montes F.R., Cabrera M., Galan A., Prieto A. (2010). Caffeine’s Vascular Mechanisms of Action. Int. J. Vasc. Med..

[78] Abad-Colil F., Ramirez-Campillo R., Alvarez C., Castro M., Silva S., Izquierdo M. (2017). Effects of beta-hydroxy-beta-methylbutyrate supplementation on physical performance of young players during an intensified soccer-training period: A short report. Human Mov. Spec. Issues.

[79] Shimomura Y., Inaguma A., Watanabe S., Yamamoto Y., Muramatsu Y., Bajotto G., Sato J., Shimomura N., Kobayashi H., Mawatari K. (2010). Branched-chain amino acid supplementation before squat exercise and delayed-onset muscle soreness. Int. J. Sport Nutr. Exerc. Metab..

[80] Wilson J.M., Fitschen P.J., Campbell B., Wilson G.J., Zanchi N., Taylor L., Wilborn C., Kalman D.S., Stout J.R., Hoffman J.R. (2013). International Society of Sports Nutrition Position Stand: Beta-hydroxy-beta-methylbutyrate (HMB). J. Int. Soc. Sports Nutr..

[81] De Lorenzo A., Petroni M.L., Masala S., Melchiorri G., Pietrantuono M., Perriello G., Andreoli A. (2003). Effect of acute and chronic branched-chain amino acids on energy metabolism and muscle performance. Diabetes Nutr. Metab..

[82] Kephart W.C., Wachs T.D., Thompson R.M., Brooks Mobley C., Fox C.D., McDonald J.R., Ferguson B.S., Young K.C., Nie B., Martin J.S. (2016). Ten weeks of branched-chain amino acid supplementation improves select performance and immunological variables in trained cyclists. Amino Acids.

[83] Howatson G., Hoad M., Goodall S., Tallent J., Bell P.G., French D.N. (2012). Exercise-induced muscle damage is reduced in resistance-trained males by branched chain amino acids: A randomized, double-blind, placebo controlled study. J. Int. Soc. Sports Nutr..

[84] Donahue P.T., Wilson S.J., Williams C.C., Hill C.M., Garner J.C. (2021). Comparison of Countermovement and Squat Jumps Performance in Recreationally Trained Males. Int. J. Exerc. Sci..

[85] Nejić K., Stanković M., Rančić D., Jelaska I., Pezelj L., Katanić B., Badau A., Badau D., Masanovic B. (2025). Associations Between Jump Performance, Speed, and COD Abilities in Young Elite Volleyball Players. Appl. Sci..

[86] Maughan R.J., Shirreffs S.M. (2017). Energy demands of volleyball. Handbook of Sports Medicine and Science.

[87] Dutka T.L., Lamboley C.R., McKenna M.J., Murphy R.M., Lamb G.D. (2012). Effects of carnosine on contractile apparatus Ca^2+^ sensitivity and sarcoplasmic reticulum Ca^2+^ release in human skeletal muscle fibers. J. Appl. Physiol. (1985).

[88] Kim K.J., Song H.S., Yoon D.H., Fukuda D.H., Kim S.H., Park D.H. (2018). The effects of 10 weeks of beta-alanine supplementation on peak power, power drop, and lactate response in Korean national team boxers. J. Exerc. Rehabil..

[89] Rebelo A., Valente-dos-Santos J., Pires I.G., Arrais I., Pereira J.R., Turner A.N. (2025). Strength and Conditioning for Volleyball: A Review. Strength Cond. J..

[90] Martinez D.B. (2017). Consideration for Power and Capacity in Volleyball Vertical Jump Performance. Strength Cond. J..

[91] Smith H.J., Mukerji P., Tisdale M.J. (2005). Attenuation of proteasome-induced proteolysis in skeletal muscle by beta-hydroxy-beta-methylbutyrate in cancer-induced muscle loss. Cancer Res..

[92] Madeira S.V., Auger C., Anselm E., Chataigneau M., Chataigneau T., Soares de Moura R., Schini-Kerth V.B. (2009). eNOS activation induced by a polyphenol-rich grape skin extract in porcine coronary arteries. J. Vasc. Res..

[93] Glaister M., Rhodes L. (2022). Short-Term Creatine Supplementation and Repeated Sprint Ability—A Systematic Review and Meta-Analysis. Int. J. Sport Nutr. Exerc. Metab..

[94] Mielgo-Ayuso J., Calleja-Gonzalez J., Marques-Jimenez D., Caballero-Garcia A., Cordova A., Fernandez-Lazaro D. (2019). Effects of Creatine Supplementation on Athletic Performance in Soccer Players: A Systematic Review and Meta-Analysis. Nutrients.

[95] Grgic J. (2018). Caffeine ingestion enhances Wingate performance: A meta-analysis. Eur. J. Sport Sci..

[96] Peeling P., Binnie M.J., Goods P.S.R., Sim M., Burke L.M. (2018). Evidence-Based Supplements for the Enhancement of Athletic Performance. Int. J. Sport Nutr. Exerc. Metab..

[97] Kaufman M.W., Roche M., Fredericson M. (2022). The Impact of Supplements on Sports Performance for the Trained Athlete: A Critical Analysis. Curr. Sports Med. Rep..

[98] Jakovljević D.K., Erić M., Jovanović G.B., Dimitrić G., Čupić M.B., Ponorac N. (2018). Explosive muscle power assessment in elite athletes using wingate anaerobic test. Rev. Bras. Med. Esporte.

[99] Berti Zanella P., Donner Alves F., Guerini de Souza C. (2017). Effects of beta-alanine supplementation on performance and muscle fatigue in athletes and non-athletes of different sports: A systematic review. J. Sports Med. Phys. Fit..

[100] Desbrow B., Burd N.A., Tarnopolsky M., Moore D.R., Elliott-Sale K.J. (2019). Nutrition for Special Populations: Young, Female, and Masters Athletes. Int. J. Sport Nutr. Exerc. Metab..

[101] Metzger G.A., Minneci P.M., Gehred A., Day A., Klingele K.E. (2023). Creatine supplementation in the pediatric and adolescent athlete—A literature review. J. Orthop..

[102] Pakulak A., Candow D.G., Totosy de Zepetnek J., Forbes S.C., Basta D. (2022). Effects of Creatine and Caffeine Supplementation During Resistance Training on Body Composition, Strength, Endurance, Rating of Perceived Exertion and Fatigue in Trained Young Adults. J. Diet. Suppl..

[103] Noma H., Tanaka S., Matsui S., Cipriani A., Furukawa T.A. (2017). Quantifying indirect evidence in network meta-analysis. Stat. Med..

[104] Culbertson J.Y., Kreider R.B., Greenwood M., Cooke M. (2010). Effects of beta-alanine on muscle carnosine and exercise performance: A review of the current literature. Nutrients.

[105] Turcu I., Oancea B., Chicomban M., Simion G., Simon S., Negriu Tiuca C.I., Ordean M.N., Petrovici A.G., Nicolescu Șeușan N.A., Hăisan P.L. (2022). Effect of 8-week β-alanine supplementation on CRP, IL-6, body composition, and bio-motor abilities in elite male basketball players. Int. J. Environ. Res. Public Health.

[106] Zajac A., Waskiewicz Z., Poprzecki S., Cholewa J. (2003). Effects of creatine and HMB supplementation on anaerobic power and body composition in basketball players. J. Hum. Kinet..

[107] Salleh R.M., Kuan G., Aziz M.N.A., Rahim M.R.A., Rahayu T., Sulaiman S., Kusuma D.W.Y., Adikari A.M.G.C.P., Razam M.S.M., Radhakrishnan A.K. (2021). Effects of probiotics on anxiety, stress, mood and fitness of badminton players. Nutrients.

[108] Abian P., Del Coso J., Salinero J.J., Gallo-Salazar C., Areces F., Ruiz-Vicente D., Lara B., Soriano L., Muñoz V., Abian-Vicen J. (2015). The ingestion of a caffeinated energy drink improves jump performance and activity patterns in elite badminton players. J. Sports Sci..

[109] Rosas F., Ramírez-Campillo R., Martínez C., Caniuqueo A., Cañas-Jamet R., McCrudden E., Meylan C., Moran J., Nakamura F.Y., Pereira L.A. (2017). Effects of plyometric training and beta-alanine supplementation on maximal-intensity exercise and endurance in female soccer players. J. Hum. Kinet..

