# Supplementary material for: Effects of Nutritional Supplements on Explosive Lower Limb Performance in Volleyball Players: A Systematic Review and Network Meta-Analysis
Source: Nutrients. 2025 Nov 26;17(23):3702. doi: 10.3390/nu17233702 (PMC12694113; doi:10.3390/nu17233702)
Supplement: Supplementary file 1 [file nutrients-17-03702-s001.zip › nutrients-3966039-supplementary materials.pdf]

Supplementary Appendix 1

Effects of Nutritional Supplements on Explosive Lower Limb Performance in Volleyball Players: A Systematic Review and Network Meta-Analysis

Haoyu Du [1], Shuning Liu [2], Mu Li [3], Kai Zhao [4,*], Wei Jiang [4], Ting You [2], Zheng Wang [5], Dixin Zou [6], Jingdan Shu [1,*] and Chang Liu [2,*]

Table of Contents

Appendix 1: Search strategy 2
Appendix 2: Risk of bias of randomized clinical trials 9
Appendix 3 - Gelman-Rubin Plots 9
Appendix 4 –Convergence Diagnostics: Trace and Density Plots 12
Appendix 5 – Visualization of Node-Splitting Analysis Results. 17
Appendix S6: Evaluation of heterogeneity 18
Appendix 7: SUCRA ranking table 19
Appendix 8: League Table of Summary Estimates for Supplements on Physical Performance of Volleyball Players from Network Meta-analysis of 36 Trials 21
Appendix 9: CINeMA Assessment 25
Appendix 10: Sensitivity analyses 36
Appendix 11 – Meta-regression analyses results 41
Appendix 12: Subgroup analysis of different supplement of physical performance on Age ... 42
Appendix 13: Subgroup analysis of different supplement of physical performance on competitive level 49
Appendix 14: Funnel Plots and Egger Tests 56

Appendix 1: Search strategy

Table S1. Search strategy of PubMed

#	Searches
1	Search: Volleyball[mh]
2	Search: Volleyball[All Fields]
3	Search: (Volleyball) OR (volleyballs)
4	Search: (#1) OR (#2) OR (#3)
5	Search: Dietary Supplements [mh]
6	Search: Dietary Supplements [All Fields]
7	Search: (Dietary Supplements) OR (Dietary Supplement) OR (Supplements, Dietary) OR (Dietary Supplementations) OR (Supplementations, Dietary) OR (Food Supplementations) OR (Food Supplements) OR (Food Supplement) OR (Supplement, Food) OR (Supplements, Food) OR (Nutraceuticals) OR (Nutraceutical) OR (Nutriceuticals) OR (Nutriceutical) OR (Neutraceuticals) OR (Neutraceutical) OR (Herbal Supplements) OR (Herbal Supplement) OR (Supplement, Herbal) OR (Supplements, Herbal)
8	Search: (#5) OR (#6) OR (#7)
9	Search: Alanine[mh]
10	Search: Alanine[All Fields]
11	Search: (Alanine)
12	Search: (#9) OR (#10) OR (#11)
13	Search: Amino Acids, Branched-Chain[mh]
14	Search: Amino Acids, Branched-Chain[All Fields]
15	Search: (Amino Acids, Branched-Chain) OR (Acids, Branched-Chain Amino) OR (Branched-Chain Amino Acids) OR

(Branched-Chain Amino Acid) OR (Acid, Branched-Chain Amino) OR (Amino Acid, Branched-Chain) OR (Branched Chain Amino Acid) OR (Amino Acids, Branched Chain)

16 Search: (#13) OR (#14) OR (#15)

17 Search: Creatine[mh]

18 Search: Creatine[All Fields]

19 Search: (Creatine)

20 Search: (#17) OR (#18) OR (#19)

21 Search: Caffeine[mh]

22 Search: Caffeine[All Fields]

23 Search: (Caffeine) OR (caffeinated)

24 Search: (#21) OR (#22) OR (#23)

25 Search: Magnesium[mh]

26 Search: Magnesium[All Fields]

27 Search: (Magnesium)

28 Search: (#25) OR (#26) OR (#27)

29 Search: beta-hydroxyisovaleric acid[mh]

30 Search: beta-hydroxyisovaleric acid[All Fields]

31 Search: (beta-hydroxyisovaleric acid) OR (beta hydroxy beta methylbutyrate) OR (beta-hydroxy beta-methylbutyrate) OR (3-hydroxyisovaleric acid) OR (beta-hydroxy-beta-methylbutyrate) OR (HMB)

32 Search: (#29) OR (#30) OR (#31)

33 Search: beetroot[All Fields]

34 Search: (beetroot) OR (beet) OR (sugar beet)

35 Search: (#33) OR (#34)

36 Search: grape[mh]

37 Search: grape[All Fields]

38 Search: (grape) OR (grape juice)

39 Search: (#36) OR (#37) OR (#38)

40 Search Energy Drinks [mh]

41 Search: Energy Drinks[All Fields]

42 Search: (Energy Drinks) OR (Drink, Energy) OR (Drinks, Energy) OR (Energy Drink) OR (Sports Drink)

43 Search: (#40) OR (#41) OR (#42)

44 Search: Arginine [mh]

45 Search: Arginine[All Fields]

46 Search: (Arginine) OR (Arginine, L-Isomer) OR (Arginine, L Isomer) OR (L-Isomer Arginine) OR (L-Arginine) OR (L Arginine) OR (DL-Arginine Acetate, Monohydrate) OR (DL Arginine Acetate, Monohydrate) OR (Monohydrate

	DL-Arginine Acetate) OR (Arginine Hydrochloride) OR (Hydrochloride, Arginine)
47	Search: (#44) OR (#45) OR (#46)
48	Search: vitamins [mh]
49	Search: vitamins[All Fields]
50	Search: (vitamins) OR (vitamin)
51	Search: (#48) OR (#49) OR (#50)
52	Search: coenzyme Q10 [mh]
53	Search: coenzyme Q10[All Fields]
54	Search: (coenzyme Q10) OR (Bio-Quinone Q10) OR (ubiquinone 10) OR (CoQ10) OR (ubidecarenone) OR (ubiquinone 50) OR (ubiquinone Q10) OR (CoQ 10) OR (co-enzyme Q10) OR (ubisemiquinone) OR (ubisemiquinone radical)
55	Search: (#52) OR (#53) OR (#54)
56	Search: Minerals[mh]
57	Search: Minerals[All Fields]
58	Search: (Minerals) OR (Mineral)
59	Search: (#56) OR (#57) OR (#58)
60	Search: (#8) OR (#12) OR (#16) OR (#20) OR (#24) OR (#28) OR (#32) OR (#35) OR (#39) OR (#43) OR (#47) OR (#51) OR (#55) OR (#59)
61	Search: (#4) AND (#60)
62	Search: Randomized Controlled Trial [mh]
63	Search: Randomized Controlled Trial[All Fields]
64	Search: (Randomized Controlled Trial) OR (Random) OR (Placebo)
65	Search: (#62) OR (#63) OR (#64)
66	Search: (#61) AND (#65)

Table S2. Search strategy of Embase

Embase

#	Searches
1	'volleyball'/exp OR volleyball
2	'volleyball':ab,ti OR 'volleyballs':ab,ti
3	#1 OR #2
4	dietary AND supplements
5	'Dietary Supplements':ab,ti OR 'Dietary Supplement':ab,ti OR 'Supplements, Dietary':ab,ti OR 'Dietary Supplementations':ab,ti OR 'Supplementations, Dietary':ab,ti OR 'Food Supplementations':ab,ti OR 'Food Supplements':ab,ti OR 'Food Supplement':ab,ti OR 'Supplement, Food':ab,ti OR 'Supplements, Food':ab,ti OR 'Nutraceuticals':ab,ti OR 'Nutraceutical':ab,ti OR

	'Nutriceuticals':ab,ti OR 'Nutriceutical':ab,ti OR 'Neutraceuticals':ab,ti OR 'Neutraceutical':ab,ti OR 'Herbal Supplements':ab,ti OR 'Herbal Supplement':ab,ti OR 'Supplement, Herbal':ab,ti OR 'Supplements, Herbal':ab,ti
6	#4 OR #5
7	'alanine'/exp OR alanine
8	'Alanine':ab,ti
9	#7 OR #8
10	amino AND acids, AND 'branched chain'
11	'Amino Acids, Branched-Chain':ab,ti OR 'Acids, Branched-Chain Amino':ab,ti OR 'Branched-Chain Amino Acids':ab,ti OR 'Branched-Chain Amino Acid':ab,ti OR 'Acid, Branched-Chain Amino':ab,ti OR 'Amino Acid, Branched-Chain':ab,ti OR 'Branched Chain Amino Acid':ab,ti OR 'Amino Acids, Branched Chain':ab,ti
12	#10 OR #11
13	creatine
14	'creatine':ab,ti
15	#13 OR #14
16	Caffeine
17	'caffeine':ab,ti OR 'caffeinated':ab,ti
18	'cohort'/expMagnesium 'Randomized Controlled Trial':ab,ti OR 'Random':ab,ti OR 'Placebo':ab,ti
19	'controlled study'/exp
20	'drug comparison'/exp
21	'clinical study'/exp
22	'observational study'/exp
23	'prospective study'/exp
24	13 OR 14 OR 15 OR 16 OR 17 OR 18 OR 19 OR 20 OR 21 OR 22 OR 23
25	'animals'/exp NOT ('humans'/exp AND 'animals'/exp)
26	24 NOT 25
27	12 AND 26

Table S3. Search strategy of Cochrane Central Register of Controlled Trials (CENTRAL)

#	Searches
1	Volleyball

2	(volleyball):ab,ti,kw OR (volleyballs):ab,ti,kw
3	#1 or #2
4	Dietary Supplements
5	(Dietary Supplements):ab,ti,kw OR (Dietary Supplement):ab,ti,kw OR (Supplements, Dietary):ab,ti,kw OR (Dietary Supplementations):ab,ti,kw OR (Supplementations, Dietary):ab,ti,kw OR (Food Supplementations):ab,ti,kw OR (Food Supplements):ab,ti,kw OR (Food Supplement):ab,ti,kw OR (Supplement, Food):ab,ti,kw OR (Supplements, Food):ab,ti,kw OR (Nutraceuticals):ab,ti,kw OR (Nutraceutical):ab,ti,kw OR (Nutriceuticals):ab,ti,kw OR (Nutriceutical):ab,ti,kw OR (Neutraceuticals):ab,ti,kw OR (Neutraceutical):ab,ti,kw OR (Herbal Supplements):ab,ti,kw OR (Herbal Supplement):ab,ti,kw OR (Supplement, Herbal):ab,ti,kw OR (Supplements, Herbal):ab,ti,kw
6	#4 or #5
7	Alanine
8	(Alanine):ab,ti,kw
9	#7 or #8
10	Amino Acids, Branched-Chain
11	(Amino Acids, Branched-Chain):ab,ti,kw OR (Acids, Branched-Chain Amino):ab,ti,kw OR (Branched-Chain Amino Acids):ab,ti,kw OR (Branched-Chain Amino Acid):ab,ti,kw OR (Acid, Branched-Chain Amino):ab,ti,kw OR (Amino Acid, Branched-Chain):ab,ti,kw OR (Branched Chain Amino Acid):ab,ti,kw OR (Amino Acids, Branched Chain):ab,ti,kw
12	#10 or #11
13	Creatine
14	(Creatine):ab,ti,kw
15	#13 or #14
16	Caffeine
17	(Caffeine):ab,ti,kw OR (Caffeinated):ab,ti,kw
18	#16 or #17
19	Magnesium
20	(Magnesium):ab,ti,kw
21	#19 or #20
22	beetroot
23	(beetroot):ab,ti,kw OR (beet):ab,ti,kw OR (sugar beet):ab,ti,kw
24	#22 or #23
25	grape
26	(grape):ab,ti,kw OR (grape juice):ab,ti,kw
27	#25 or #26

28	beta-hydroxyisovaleric acid
29	(beta hydroxyisovaleric acid):ab,ti,kw OR (beta hydroxy beta methylbutyrate):ab,ti,kw OR (beta-hydroxy beta-methylbutyrate):ab,ti,kw OR (3hydroxyisovaleric acid):ab,ti,kw OR (beta hydroxy beta methylbutyrate):ab,ti,kw OR (HMB):ab,ti,kw
30	#28 or #29
31	Energy Drinks
32	(Energy Drinks):ab,ti,kw OR (Drink, Energy):ab,ti,kw OR (Drinks, Energy):ab,ti,kw OR (Energy Drink):ab,ti,kw OR (Sports Drink):ab,ti,kw
33	#31 or #32
34	Arginine
35	(Arginine):ab,ti,kw OR (Arginine, L-Isomer):ab,ti,kw OR (Arginine, L Isomer):ab,ti,kw OR (L-Isomer Arginine):ab,ti,kw OR (L-Arginine):ab,ti,kw OR (L Arginine):ab,ti,kw OR (DL-Arginine Acetate, Monohydrate):ab,ti,kw OR (DL Arginine Acetate, Monohydrate):ab,ti,kw OR (Monohydrate DL-Arginine Acetate):ab,ti,kw OR (Arginine Hydrochloride):ab,ti,kw OR (Hydrochloride, Arginine):ab,ti,kw
36	#34 or #35
37	vitamins
38	(vitamins):ab,ti,kw OR (vitamin):ab,ti,kw
39	#37 or #38
40	coenzyme Q10
41	(coenzyme Q10):ab,ti,kw OR (Bio-Quinone Q10):ab,ti,kw OR (ubiquinone 10):ab,ti,kw OR (CoQ10):ab,ti,kw OR (ubidecarenone):ab,ti,kw OR (ubiquinone 50):ab,ti,kw OR (ubiquinone Q10):ab,ti,kw OR (CoQ 10):ab,ti,kw OR (co-enzyme Q10):ab,ti,kw OR (ubisemiquinone):ab,ti,kw OR (ubisemiquinone radical):ab,ti,kw
42	#40 or #41
43	Minerals
44	(Minerals):ab,ti,kw OR (Mineral):ab,ti,kw
45	#43 or #44
46	#6 or #9 or #12 or #15 or #18 or #21 or #24 or #27 or #30 or #33 or #36 or #39 or #42 or #45
47	Randomized Controlled Trial
48	(Randomized Controlled Trial):ab,ti,kw OR (Random):ab,ti,kw OR (Placebo):ab,ti,kw
49	#47 or #48
50	#3 and #46 and #49

Table S4. Search strategy of Web of Science: Science Citation Index Expanded

#	Searches
1	TS=(volleyball)
2	(TS=(volleyball)) OR TS=(volleyballs)
3	((((((((((((((((((TS=(Dietary Supplements)) OR TS=(Dietary Supplement)) OR TS=(Supplements, Dietary)) OR TS=(Dietary Supplementations)) OR TS=(Supplementations, Dietary)) OR TS=(Food Supplementations)) OR TS=(Food Supplements)) OR TS=(Food Supplement)) OR TS=(Supplement, Food)) OR TS=(Supplements, Food)) OR TS=(Nutraceuticals)) OR TS=(Nutraceutical)) OR TS=(Nutriceuticals)) OR TS=(Nutriceutical)) OR TS=(Neutraceuticals)) OR TS=(Neutraceutical)) OR TS=(Herbal Supplements)) OR TS=(Herbal Supplement)) OR TS=(Supplement, Herbal)) OR TS=(Supplements, Herbal)
4	TS=(Alanine)
5	(((((((TS=(Amino Acids, Branched-Chain)) OR TS=(Acids, Branched-Chain Amino)) OR TS=(Branched-Chain Amino Acids)) OR TS=(Branched-Chain Amino Acid)) OR TS=(Acid, Branched-Chain Amino)) OR TS=(Amino Acid, Branched-Chain)) OR TS=(Branched Chain Amino Acid)) OR TS=(Amino Acids, Branched Chain)
6	TS=(Creatine)
7	(((((((TS=(Caffeine)) OR TS=(Caffeinated)) OR TS=(Magnesium)) OR TS=(beetroot)) OR TS=(beet)) OR TS=(sugar beet)) OR TS=(grape)) OR TS=(grape juice)
8	((((((((((((((((((((TS=(beta-hydroxyisovaleric acid)) OR TS=(beta hydroxy beta methylbutyrate)) OR TS=(beta-hydroxy beta-methylbutyrate)) OR TS=(3-hydroxyisovaleric acid)) OR TS=(beta-hydroxy-beta-methylbutyrate)) OR TS=(HMB)) OR TS=(Energy Drinks)) OR TS=(Drink, Energy)) OR TS=(Drinks, Energy)) OR TS=(Energy Drink)) OR TS=(Sports Drink)) OR TS=(Arginine)) OR TS=(Arginine, L-Isomer)) OR TS=(Arginine, L Isomer)) OR TS=(L-Isomer Arginine)) OR TS=(L-Arginine)) OR TS=(DL-Arginine Acetate, Monohydrate)) OR TS=(Monohydrate DL-Arginine Acetate)) OR TS=(Arginine Hydrochloride)) OR TS=(Hydrochloride, Arginine)
9	(((((((((((((TS=(vitamins)) OR TS=(vitamin)) OR TS=(coenzyme Q10)) OR TS=(Bio-Quinone Q10)) OR TS=(ubiquinone 10)) OR TS=(CoQ10)) OR TS=(ubidecarenone)) OR TS=(ubiquinone 50)) OR

	TS=(ubiquinone Q10)) OR TS=(CoQ 10)) OR TS=(co-enzyme Q10)) OR TS=(ubisemiquinone)) OR TS=(ubisemiquinone radical)) OR TS=(Minerals)) OR TS=(Mineral)
10	((TS=(Randomized Controlled Trial)) OR TS=(Random)) OR TS=(Placebo)
11	#3 OR #4 OR #5 OR #6 OR #7 OR #8 OR #9
12	#11 AND #10 AND #2

Appendix 2: Risk of bias of randomized clinical trials

Figure S2: Overall risk of bias presented as percentage of each risk of bias item across all included studies.

Green = Low risk, Red = High risk, Yellow = Some concerns.

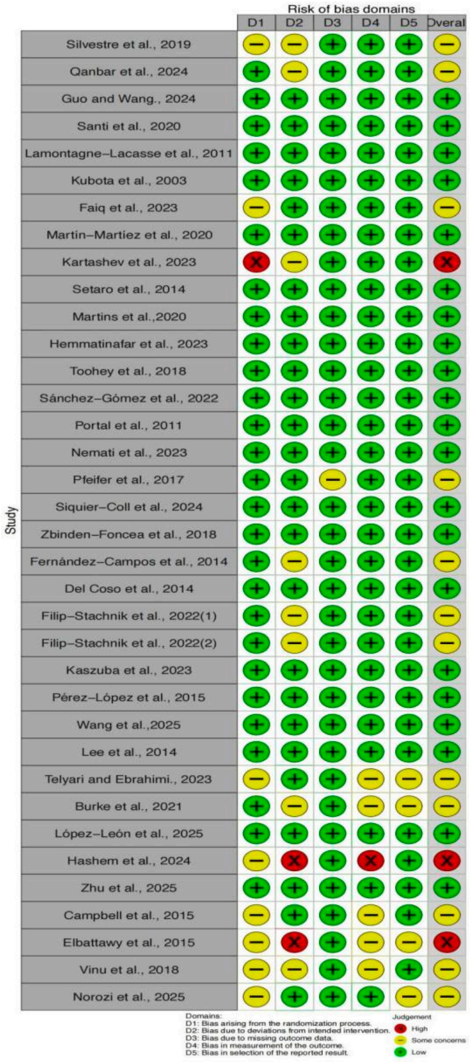

Appendix 3 - Gelman-Rubin Plots

Convergence was evaluated using the Gelman–Rubin statistic (R-hat). Values approaching 1.0 indicate satisfactory convergence, whereas values substantially greater than 1 suggest potential problems with chain mixing. In line with common

practice, R-hat values below 1.05 were considered acceptable. All monitored parameters demonstrated convergence within 25,000 iterations, as shown in the Gelman–Rubin plots.Gelman-Rubin-Brooks . The reduction factor begins to converge at 10,000-20,000 iterations, and after 20,000 iterations, the reduction factor tends to 1, reaching stability.

Figure S3.1: Vertical Jump

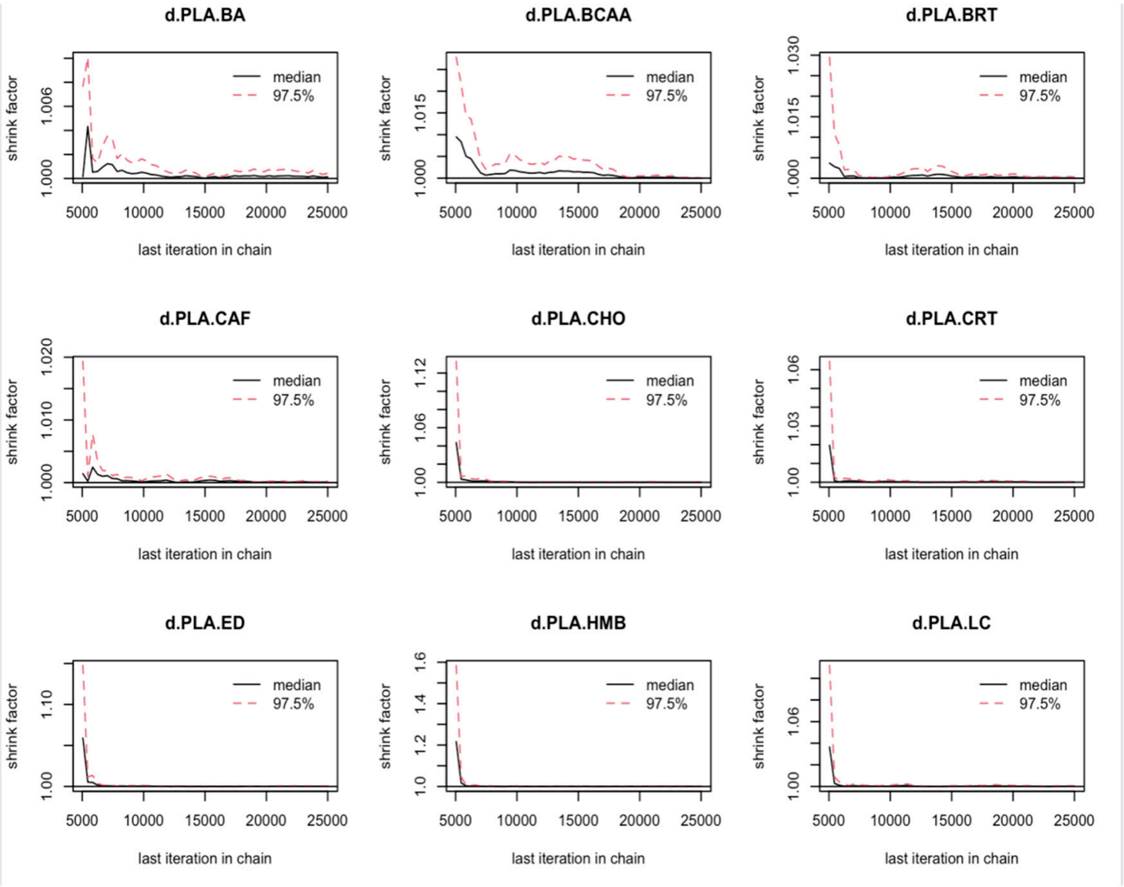

Figure S3.2: Lower limb Peak Power

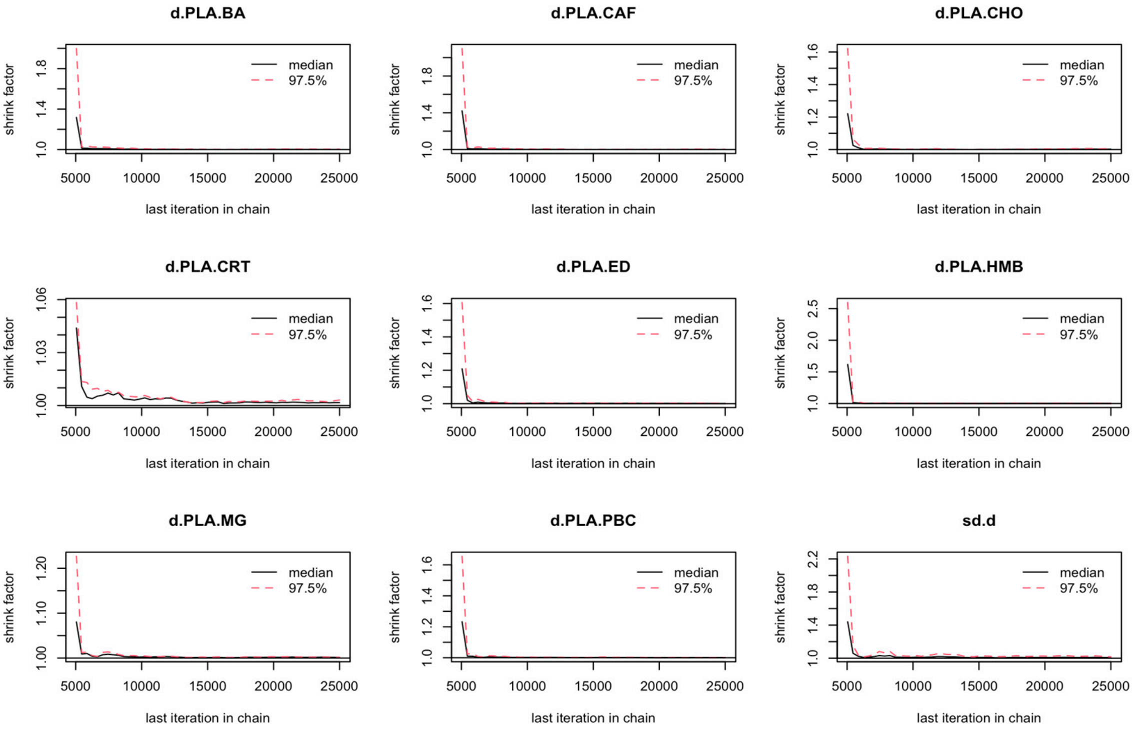

Figure S3.3: Lower limb Mean Power

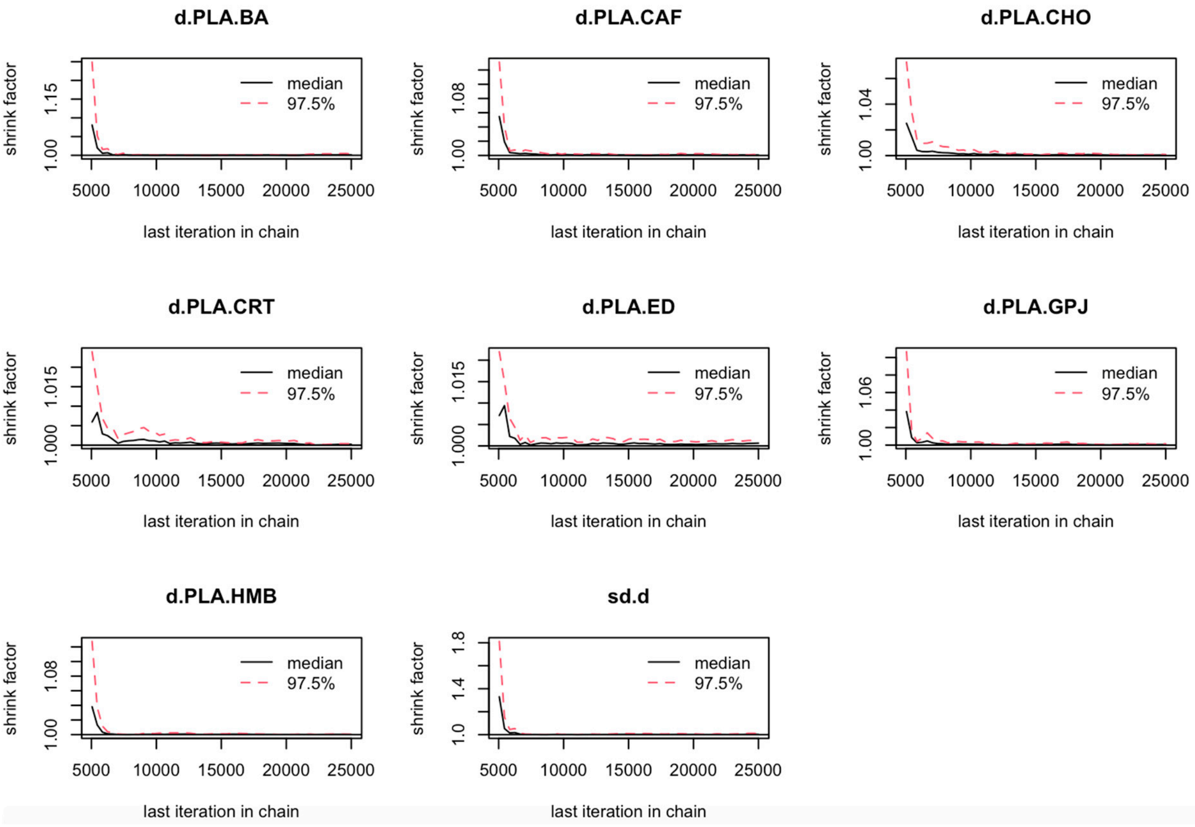

Appendix 4 –Convergence Diagnostics: Trace and Density Plots

Convergence of the Bayesian network meta-analysis models was evaluated using trace and posterior density plots.

Trace plots provide a visual representation of the sampling behavior across Markov chains. The iteration trajectory and the iteration of each contrast in the density map are basically smooth. When the adaptation length is 5000 and the number of iterations is 25000, the iteration of each contrast is basically smooth. The density map N=20000, the Bandwidth value also tends to zero and reaches stability.Posterior density plots illustrate the distributional characteristics of the sampled parameters. Unimodal, symmetric posterior distributions without irregularities or multimodal patterns further support the adequacy of model convergence. For all outcomes, the posterior densities were unimodal and well behaved, reinforcing the reliability of the estimated effects.

Figure S4.1: Vertical Jump

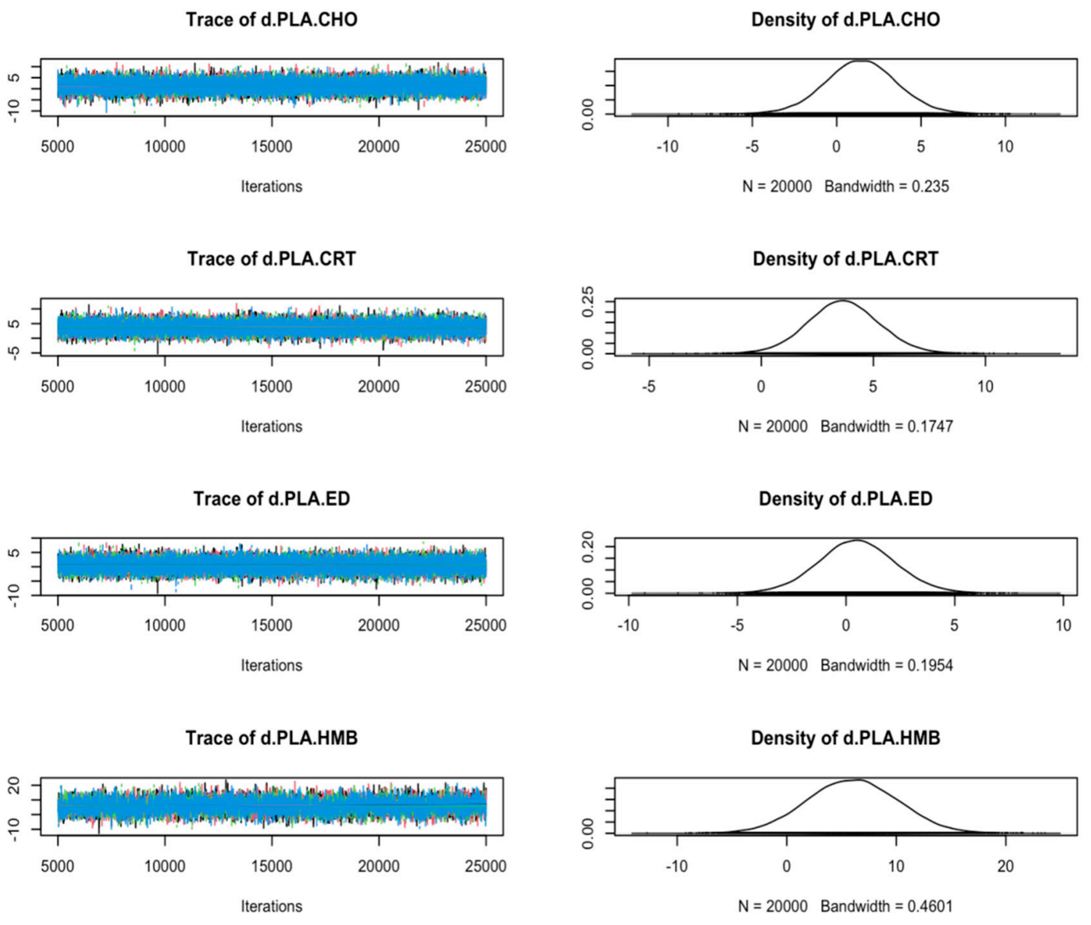

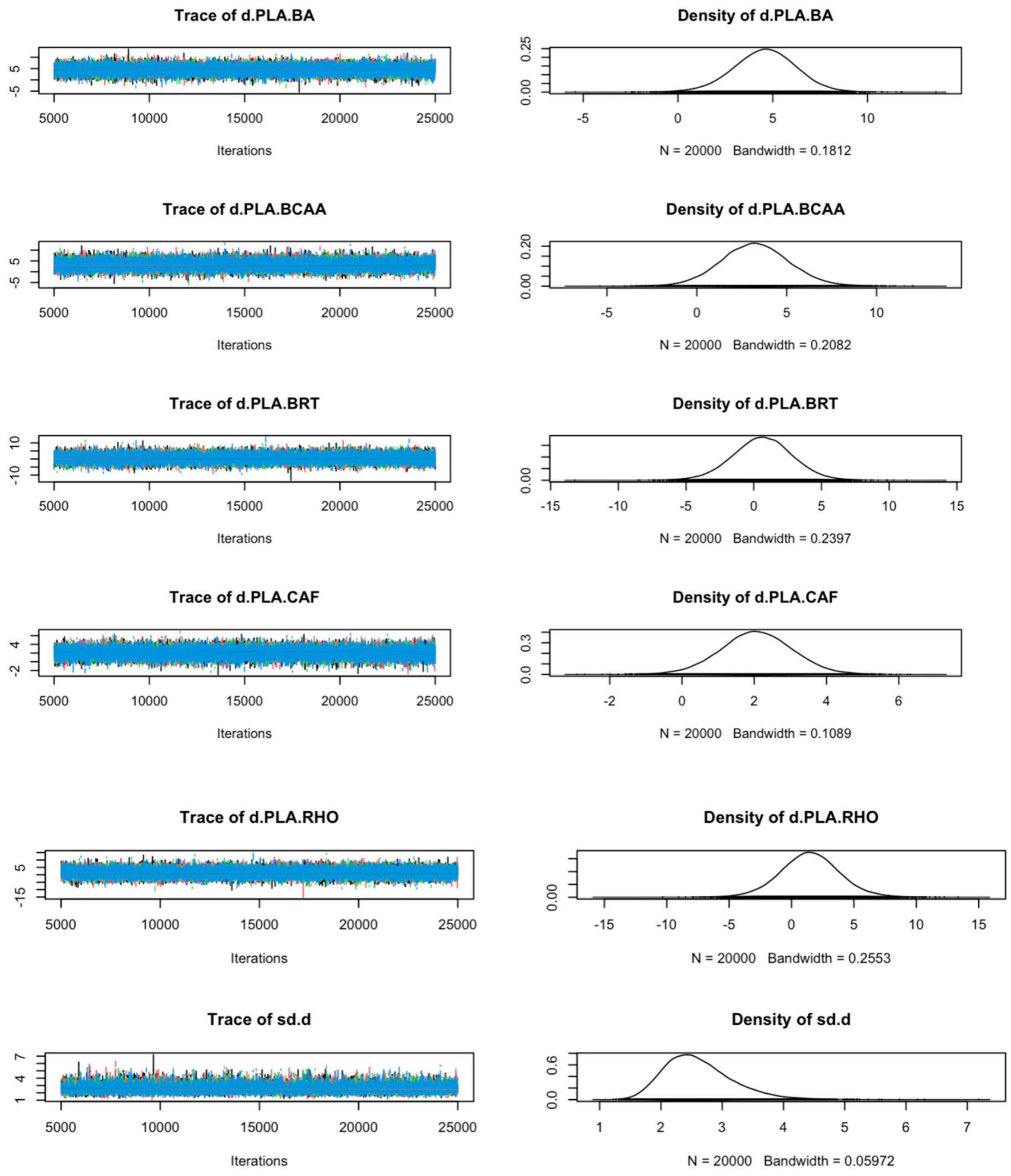
Trace of d.PLA.BA
Iterations
Density of d.PLA.BA
N = 20000 Bandwidth = 0.1812
Trace of d.PLA.BCAA
Iterations
Density of d.PLA.BCAA
N = 20000 Bandwidth = 0.2082
Trace of d.PLA.BRT
Iterations
Density of d.PLA.BRT
N = 20000 Bandwidth = 0.2397
Trace of d.PLA.CAF
Iterations
Density of d.PLA.CAF
N = 20000 Bandwidth = 0.1089
Trace of d.PLA.RHO
Iterations
Density of d.PLA.RHO
N = 20000 Bandwidth = 0.2553
Trace of sd.d
Iterations
Density of sd.d
N = 20000 Bandwidth = 0.05972

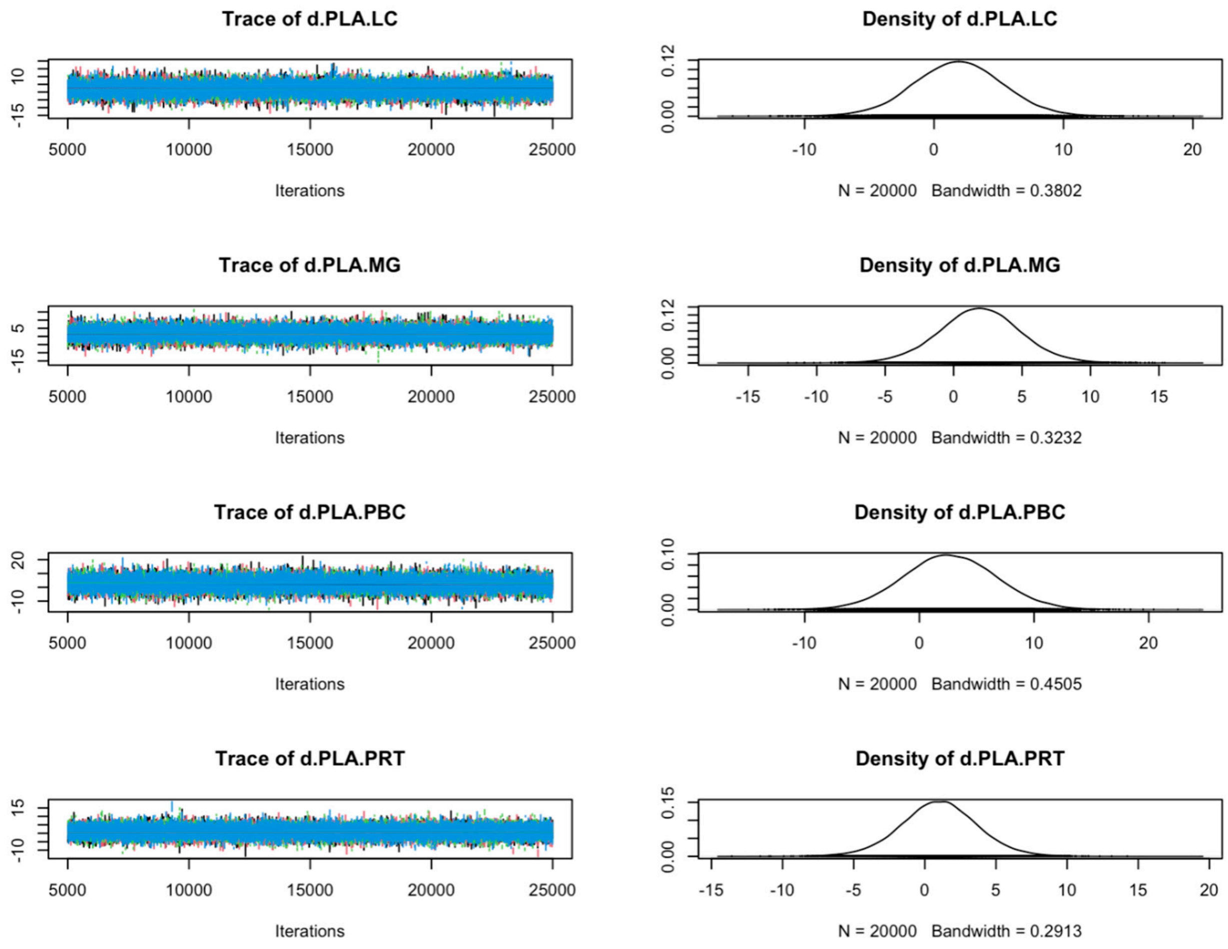

Figure S4.2: Lower limb Peak Power

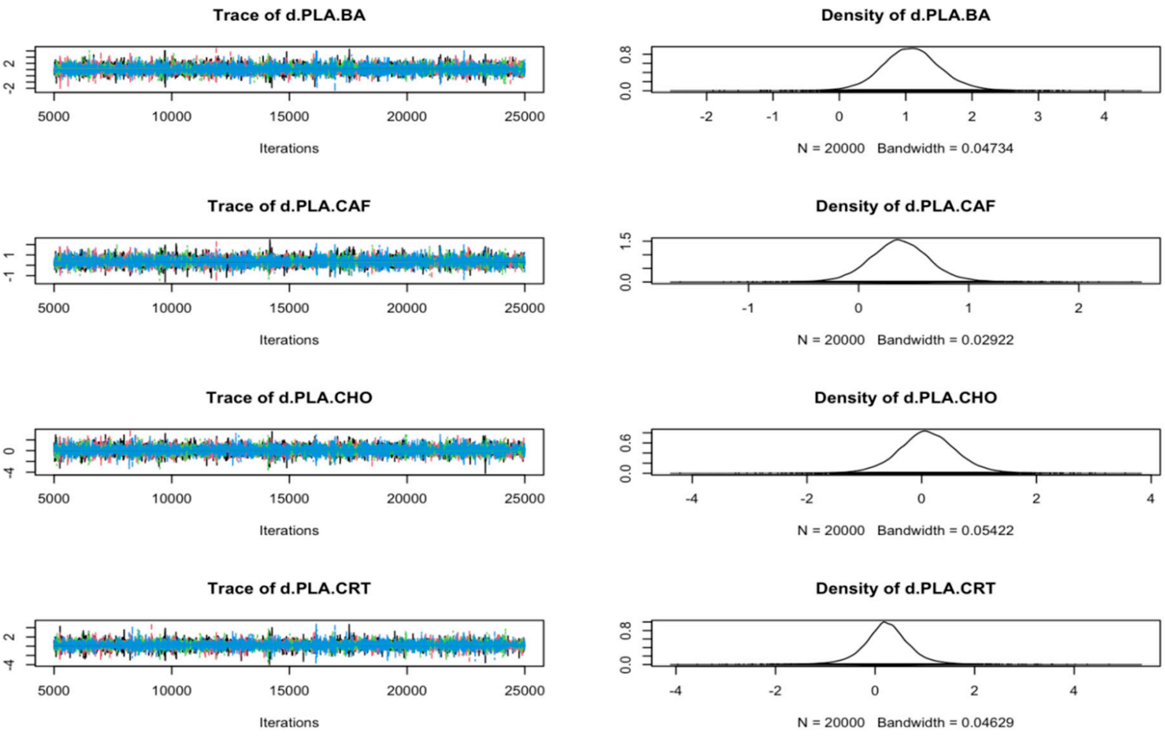

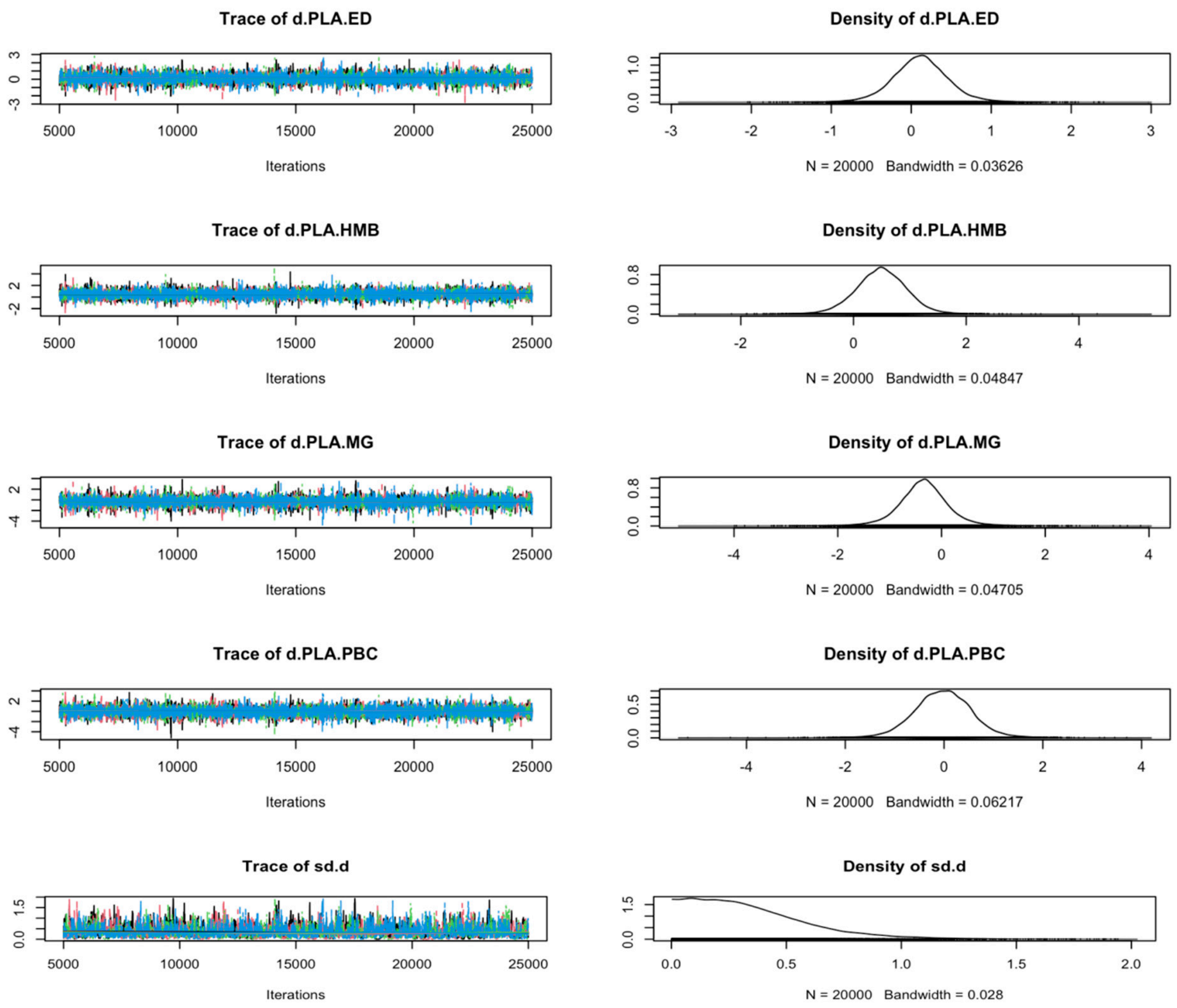

Figure S4.3: Lower limb mean power

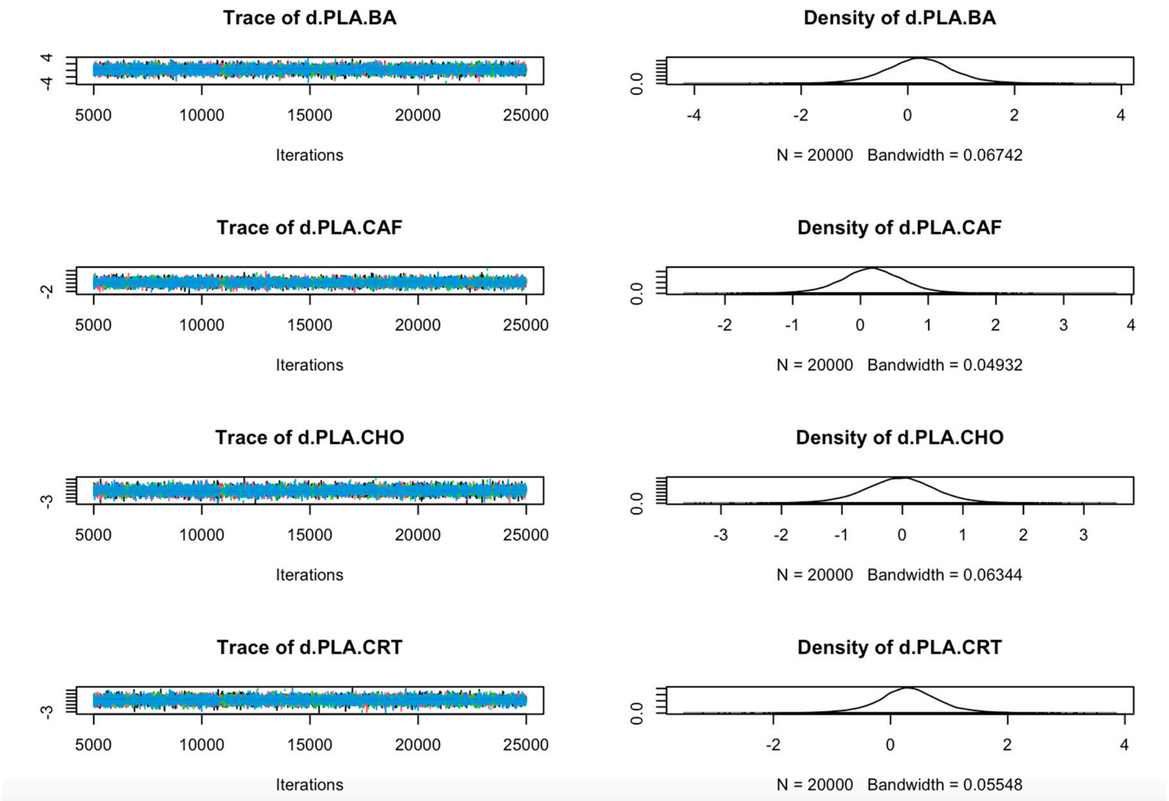
Trace of d.PLA.BA
Iterations
Density of d.PLA.BA
N = 20000 Bandwidth = 0.06742
Trace of d.PLA.CAF
Iterations
Density of d.PLA.CAF
N = 20000 Bandwidth = 0.04932
Trace of d.PLA.CHO
Iterations
Density of d.PLA.CHO
N = 20000 Bandwidth = 0.06344
Trace of d.PLA.CRT
Iterations
Density of d.PLA.CRT
N = 20000 Bandwidth = 0.05548

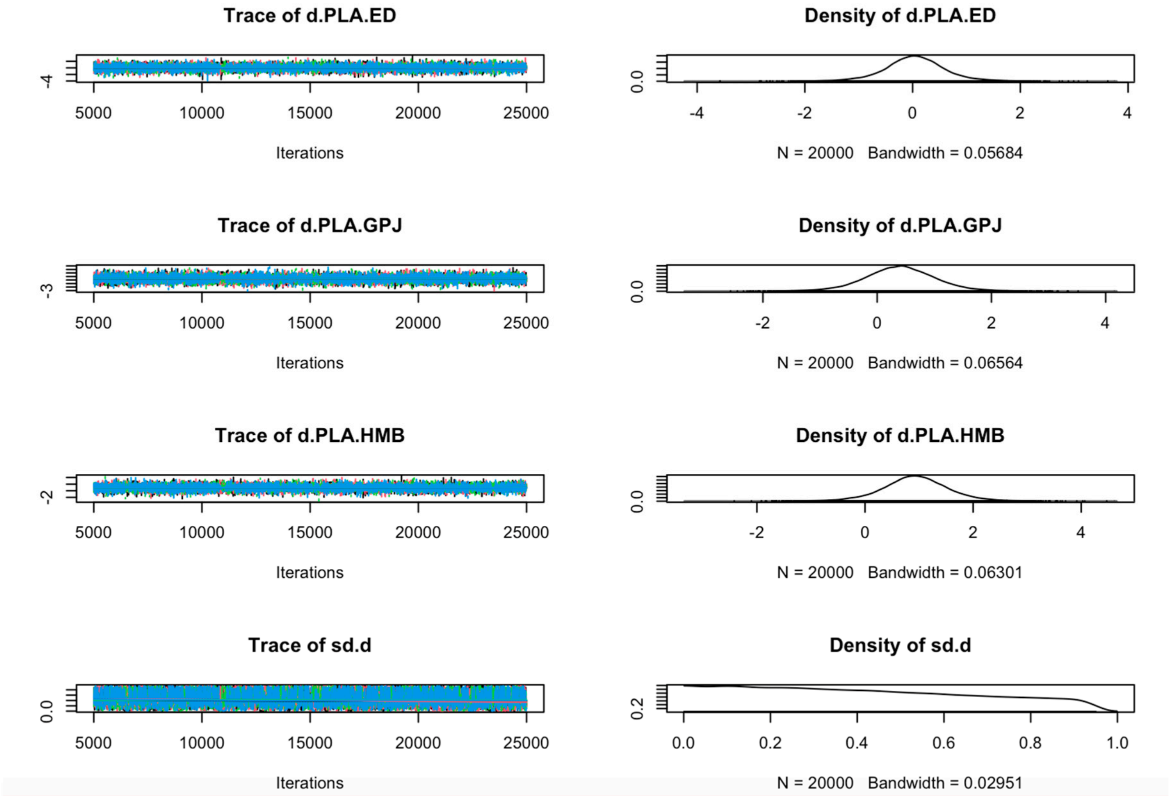
Trace of d.PLA.ED
Iterations
Density of d.PLA.ED
N = 20000 Bandwidth = 0.05684
Trace of d.PLA.GPJ
Iterations
Density of d.PLA.GPJ
N = 20000 Bandwidth = 0.06564
Trace of d.PLA.HMB
Iterations
Density of d.PLA.HMB
N = 20000 Bandwidth = 0.06301
Trace of sd.d
Iterations
Density of sd.d
N = 20000 Bandwidth = 0.02951

Appendix 5 – Visualization of Node-Splitting Analysis Results.

The plot illustrates the outcomes of a node-splitting analysis within a Bayesian network meta-analysis. Each node represents a treatment comparison, and the distribution of node-splitting statistics is depicted to assess the consistency between direct and indirect evidence for each comparison. Consistent results are indicative of agreement between the two types of evidence, while discrepancies may highlight potential inconsistencies. This graphical representation aids in evaluating the robustness and reliability of the network meta-analysis findings.

Figure S5.1: Vertical Jump consistency

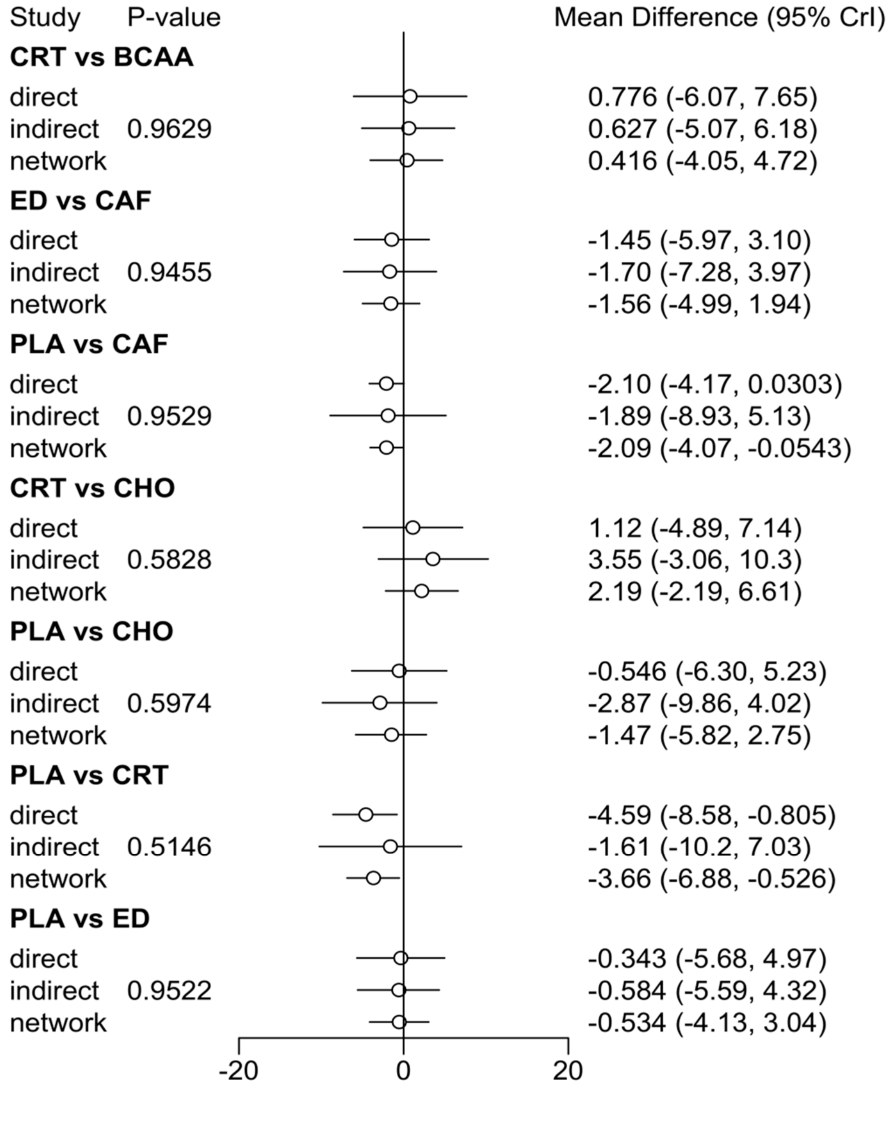

Figure S5.2: Lower limb peak power consistency

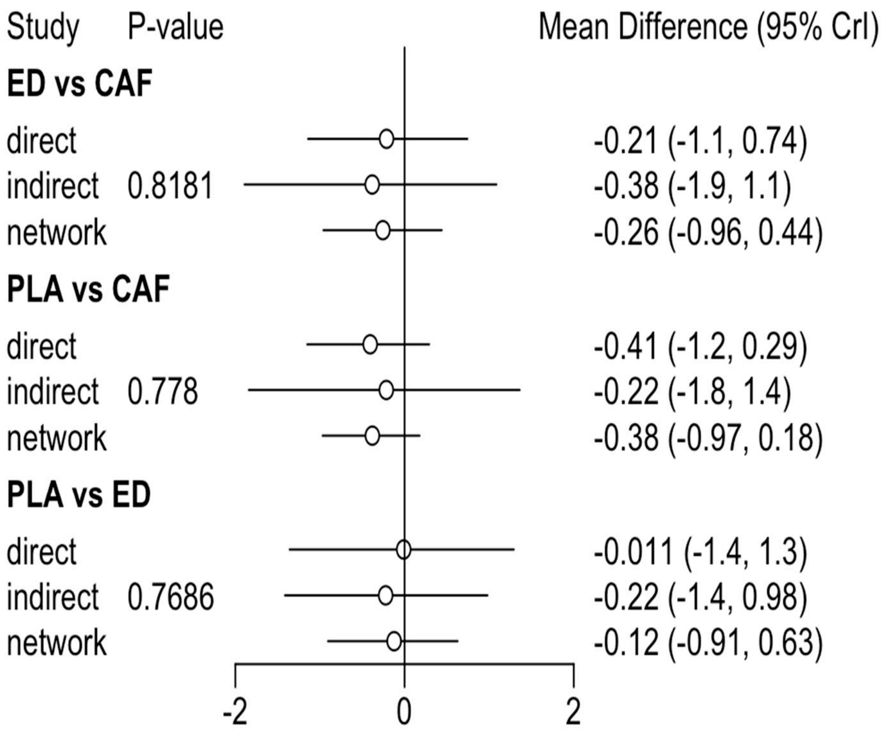

Appendix S6: Evaluation of heterogeneity

Table S6.1 Network Heterogeneity Assessment Results of Vertical Jump

I^2 .pair	I^2 .cons
**86.37%**	**83.79%**

Table S6.2 Network Heterogeneity Assessment Results of Lower limb Peak Power

I^2 .pair	I^2 .cons
**29.3%**	**9.6%**

Table S6.3 Network Heterogeneity Assessment Results of Lower limb Mean Power

I^2 .pair	I^2 .cons
**0%**	**0%**

Appendix 7: SUCRA ranking table

The rank heat plot presents a summary of P scores (range 0-100) for each intervention across outcomes, where darker shades of green represent more benefit and darker shades of red represent less benefit. BA= β -alanine; HMB= β -hydroxy- β -methylbutyrate;CRT=Creatine; BCAA=Branched-Chain Amino Acids; PBC=Probiotics; CAF=Caffeine; MG=Magnesium; LC=L-Carnitine; RHO=Rhodiola Rosea; CHO=Carbohydrate; PRT=Protein; BRT=Beetroot; ED=Energy Drink; PLA=Placebo;

Table S7.1: SUCRA of the effects of different dietary supplement on Vertical Jump.

Treatment	SUCRA
BA	79.8
HMB	79.5
CRT	71.1
BCAA	64.3
PBC	54.9
CAF	50.9
MG	47.8
LC	47.5
RHO	42.7
CHO	42.1
PRT	37.5
BRT	32.4
ED	29.5
PLA	19.7

Table S7.2: SUCRA of the effects of different dietary supplement on Lower limb Peak Power

Treatment	SUCRA
BA	93.5
HMB	67.2
CAF	65.6
CRT	52.9
ED	43.2
CHO	42.1
PBC	35.6
PLA	32.9
MG	17

Table S7.3: SUCRA of the effects of different dietary supplement on Lower limb Mean Power

Treatment	SUCRA
HMB	84.7
GPJ	59.3
CRT	55.5
BA	48.6
CAF	47.8

ED	37.7
CHO	33.8
PLA	32.5

Appendix 8: League Table of Summary Estimates for Supplements on Physical Performance of Volleyball Players from Network Meta-analysis of 36 Trials

Table S8.1: Vertcial Jump

The columns represent the comparison of the row supplement category to the column supplement category.The rows represent the comparison of the row supplement category to the column supplement category.The effect estimates are expressed as mean difference and 95% confidence interval.For example, the mean difference between BA and BCAA is 1.41 (95% confidence interval -3.8 to 6.25).A mean difference <0 favors the supplement in the column, and a mean difference >0 favors the supplement in the row.

BA					
1.41 (-3.8,6.25)	BCAA				
3.97 (-1.58, 9.4)	2.57 (-3.08, 8.46)	BRT			
2.54 (-1.38, 6.33)	1.14 (-2.99, 5.52)	-1.44 (-6.25, 3.39)	CAF		
3.14 (-2.43, 8.43)	1.74 (-3.7, 7.3)	-0.83 (-7.03, 5.28)	0.59 (-4.19, 5.32)	CHO	
0.92 (-3.8, 5.37)	-0.47 (-4.73, 3.94)	-3.04 (-8.52, 2.3)	-1.61 (-5.42, 2.07)	-2.2 (-6.62, 2.16)	CRT

4.09 (-0.86, 8.89)	2.69 (-2.4, 7.98)	0.12 (-5.53, 5.76)	1.55 (-1.89, 5)	0.97 (-4.63, 6.58)	3.16 (-1.53, 7.99)	ED							
-1.34 (-10.04, 7.34)	-2.72 (-11.5, 6.32)	-5.31 (-14.46, 3.93)	-3.87 (-12.2, 4.49)	-4.45 (-13.53, 4.65)	-2.25 (-10.81, 6.43)	-5.43 (-14.23, 3.45)	HMB						
2.73 (-4.82, 10.15)	1.32 (-6.31, 9.17)	-1.25 (-9.26, 6.82)	0.18 (-6.87, 7.29)	-0.41 (-8.37, 7.69)	1.8 (-5.57, 9.44)	-1.39 (-8.95, 6.3)	4.09 (-6.47, 14.46)	LC					
2.7 (-4.12, 9.41)	1.29 (-5.55, 8.41)	-1.27 (-8.66, 6.01)	0.16 (-6.09, 6.43)	-0.42 (-7.71, 6.85)	1.77 (-4.89, 8.51)	-1.39 (-8.28, 5.52)	4.04 (-5.96, 14)	-0.01 (-8.91, 8.92)	MG				
1.9 (-6.7, 10.51)	0.51 (-8.16, 9.47)	-2.08 (-11.17, 7.06)	-0.63 (-8.84, 7.61)	-1.24 (-10.23, 7.88)	0.98 (-7.48, 9.6)	-2.2 (-10.84, 6.63)	3.25 (-8.05, 14.46)	-0.84 (-11.2, 9.7)	-0.8 (-10.69, 9.09)	PBC			
4.61 (1.23, 7.82)	3.21 (-0.43, 7.07)	0.65 (-3.73, 5.01)	2.07 (0.06, 4.06)	1.49 (-2.8, 5.78)	3.68 (0.57, 6.89)	0.51 (-3.04, 4.05)	5.96 (-2.2, 13.96)	1.88 (-4.91, 8.6)	1.91 (-4.02, 7.78)	2.7 (-5.34, 10.66)	PLA		
3.62 (-2.79, 9.87)	2.22 (-4.21, 8.97)	-0.35 (-7.28, 6.6)	1.09 (-4.69, 6.83)	0.48 (-6.4, 7.45)	2.69 (-3.53, 9.04)	-0.47 (-6.91, 6.01)	4.96 (-4.84, 14.65)	0.9 (-7.79, 9.57)	0.93 (-7.08, 8.93)	1.71 (-7.94, 11.29)	-0.99 (-6.4, 4.44)	PRT	
3.13 (-2.75, 8.87)	1.73 (-4.21, 7.91)	-0.83 (-7.34, 5.66)	0.58 (-4.21, 5.37)	0.01 (-6.4, 6.42)	2.2 (-3.42, 8.01)	-0.98 (-6.72, 4.81)	4.46 (-4.92, 13.84)	0.4 (-7.9, 8.63)	0.42 (-7.21, 8.02)	1.21 (-8.09, 10.47)	-1.48 (-6.21,3.32)	-0.5 (-7.7, 6.71)	RHO

Table S8.2: Lower limb Peak Power

The columns represent the comparison of the row supplement category to the column supplement category.The rows represent the comparison of the row supplement category to the column supplement category.The effect estimates are expressed as mean difference and 95% confidence interval.For example, the mean difference between BA and CAF is 0.71 (95% confidence interval -0.36 to 1.79).A mean difference <0 favors the supplement in the column, and a mean difference >0 favors the supplement in the row.

BA								
0.71 (-0.36, 1.79)	CAF							
1 (-0.39, 2.41)	0.3 (-0.79, 1.37)	CHO						
0.84 (-0.51, 2.27)	0.14 (-1.03, 1.36)	-0.16 (-1.64, 1.38)	CRT					
0.97 (-0.19, 2.15)	0.26 (-0.42, 0.94)	-0.03 (-1.27, 1.19)	0.13 (-1.21, 1.39)	ED				
0.62 (-0.67, 1.94)	-0.09 (-1.17, 1)	-0.39 (-1.78, 1.05)	-0.23 (-1.65, 1.17)	-0.35 (-1.54, 0.84)	HMB			
1.42 (0.07, 2.87)	0.72 (-0.46, 1.94)	0.42 (-1.06, 1.94)	0.58 (-0.94, 2.08)	0.45 (-0.83, 1.77)	0.81 (-0.6, 2.23)	MG		
1.12 (-0.35, 2.63)	0.41 (-0.89, 1.76)	0.12 (-1.47, 1.75)	0.27 (-1.31, 1.85)	0.15 (-1.23, 1.59)	0.5 (-1, 2.04)	-0.31 (-1.88, 1.27)	PBC	
1.08 (0.19, 2.02)	0.38 (-0.18, 0.96)	0.08 (-0.98, 1.19)	0.24 (-0.83, 1.3)	0.11 (-0.62, 0.88)	0.46 (-0.45, 1.4)	-0.34 (-1.41, 0.72)	-0.03 (-1.24, 1.15)	PLA

Table S8.3: Lower limb Mean Power

The columns represent the comparison of the row supplement category to the column supplement category.The rows represent the comparison of the row supplement category to the column supplement category.The effect estimates are expressed as mean difference and 95% confidence interval.For example, the mean difference between BA and CAF is 0.01 (95% confidence interval -1.61 to 1.68).A mean difference <0 favors the supplement in the column, and a mean difference >0 favors the supplement in the row.

BA							
0.03 (-1.61, 1.68)	CAF						
0.24 (-1.55, 2.06)	0.22 (-1, 1.45)	CHO					
-0.11 (-1.89, 1.71)	-0.13 (-1.65, 1.38)	-0.36 (-2.06, 1.35)	CRT				
0.17 (-1.62, 1.97)	0.15 (-1.39, 1.68)	-0.06 (-1.79, 1.64)	0.29 (-1.42, 1.98)	ED			
-0.19 (-2.04, 1.69)	-0.22 (-1.8, 1.37)	-0.43 (-2.21, 1.32)	-0.08 (-1.84, 1.66)	-0.37 (-2.14, 1.39)	GPJ		
-0.73 (-2.58, 1.09)	-0.76 (-2.35, 0.82)	-0.99 (-2.75, 0.77)	-0.63 (-2.38, 1.11)	-0.92 (-2.67, 0.83)	-0.56 (-2.35, 1.26)	HMB	
0.2 (-1.12,	0.18	-0.04	0.31	0.02	0.39	0.94	PLA

1.54)	(-0.77, 1.12)	(-1.26, 1.18)	(-0.89, 1.5)	(-1.18, 1.24)	(-0.89, 1.7)	(-0.31, 2.22)	

Appendix 9: CINeMA Assessment

We use the CINeMA framework to evidence certainty, assessing it for each network estimate based on the following criteria:

A: Within study bias: We classified the overall risk of bias for each study as low risk of bias, the risk of bias as moderate when none of the four assessed risk
of bias items were rated as high risk, and the risk of bias as high when one or both items were rated as high risk. See Appendix2 for the bias assessment. The risk of bias for a pairwise comparison of each drug is shown in figure S9.1-9.6.

Figure S9.1: Risk of bias contribution by intervention group in Vertical Jump

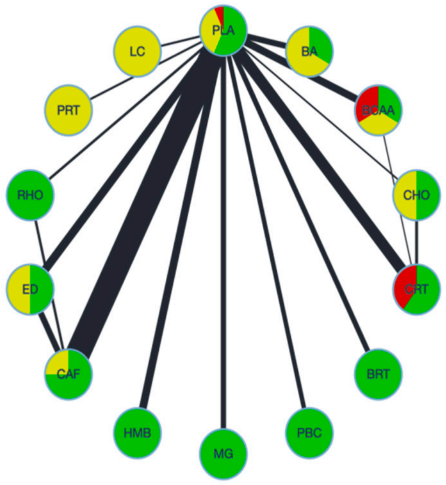

Figure S9.2: Overall risk of bias by treatment comparison in Vertical Jump

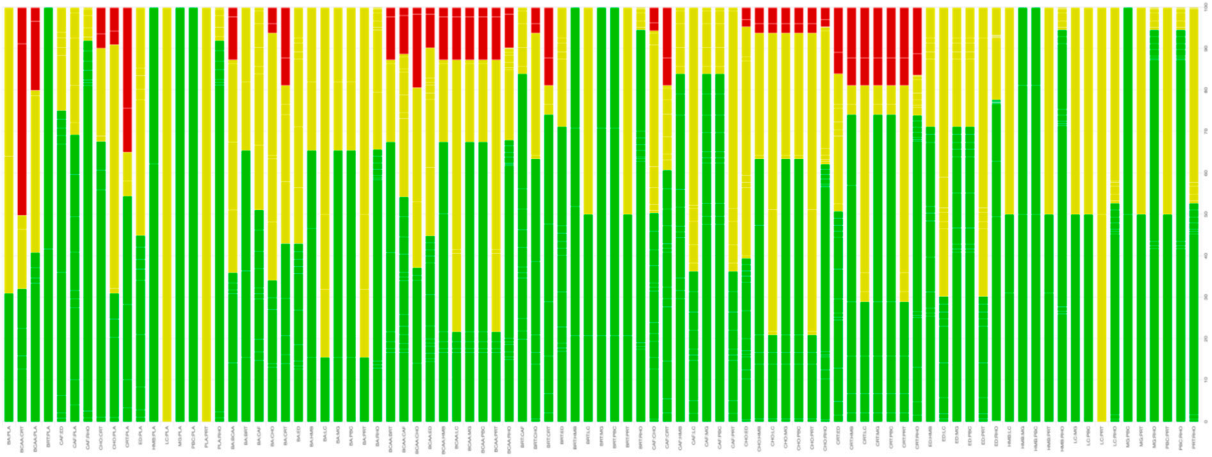

Figure S9.3: Risk of bias contribution by intervention group in Lower limb Peak Power

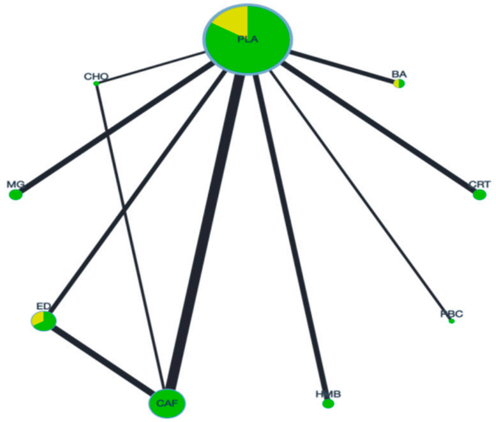

Figure S9.4: Overall risk of bias by treatment comparison in Lower limb Peak Power

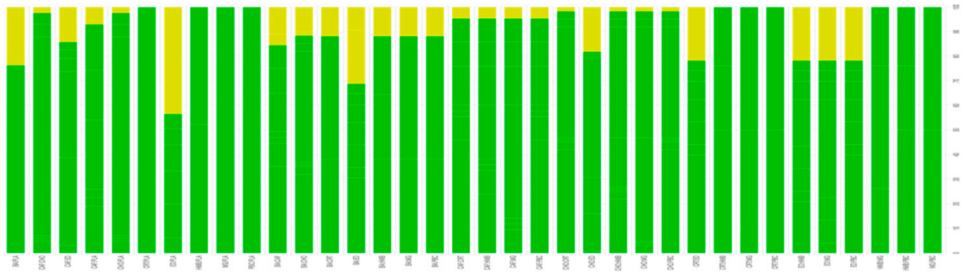

Figure S9.5: Risk of bias contribution by intervention group in Lower limb Mean Power

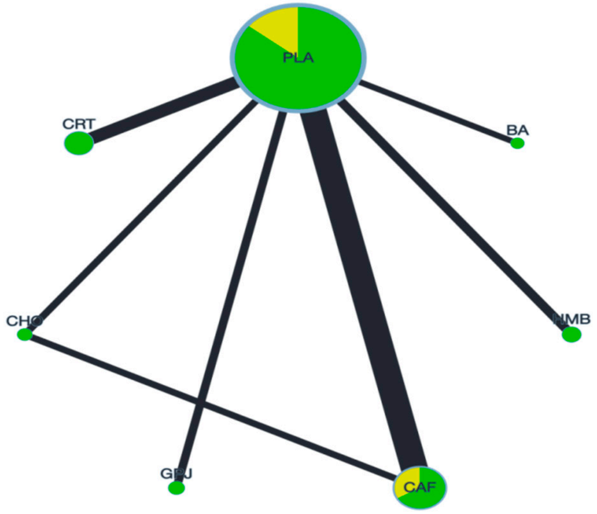

Figure S9.6: Overall risk of bias by treatment comparison in Lower limb Mean Power

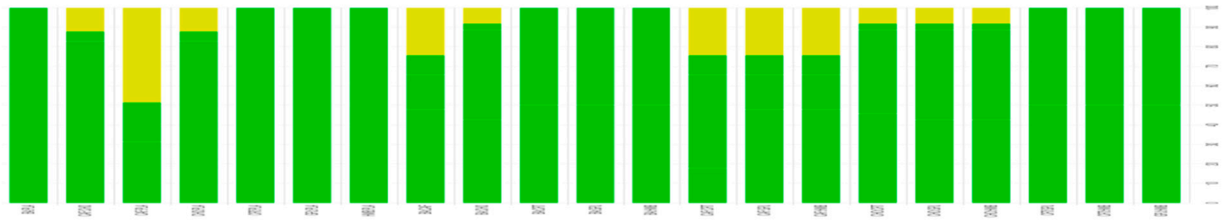

Reporting bias: All reporting bias are low risk.

Indirectness: We assessed applicability by examining baseline characteristics (age, sex, competitive level) of the included populations. Most studies enrolled elite or non-elite volleyball athletes, with supplement interventions (creatine, HMB, BCAA, caffeine) delivered in doses and durations relevant to practice. Outcomes (vertical jump, agility, Lower-Limb power, fatigue index) were directly related to volleyball performance. As all studies were conducted in volleyball players and outcomes reflected performance-relevant constructs, we judged the risk of indirectness as low.

Imprecision:

We assessed the precision of each comparison using the CINeMA platform, which grades accuracy based on confidence intervals.

Heterogeneity:

We evaluated heterogeneity by applying the same clinical reasoning framework as for imprecision, focusing on whether 95% confidence and prediction intervals crossed thresholds of clinical significance. We adopted the thresholds described above and followed CINeMA's recommendations.

Inconsistency:

We examined local inconsistency through node-splitting analyses (Appendix 5), considering $p < 0.10$ as evidence of major inconsistency.

Table S9.1: CINeMA Results of Vertical Jump

Comparison	Within-study bias	Reporting bias	Indirectness	Imprecision	Heterogeneity	Incoherence	Confidence rating
BA:PLA	Some concerns	Low risk	No concerns	No concerns	Some concerns	No concerns	Moderate
BCAA:CRT	Some concerns	Low risk	No concerns	Major concerns	No concerns	No concerns	Low
BCAA:PLA	Some concerns	Low risk	No concerns	Some concerns	Some concerns	No concerns	Moderate
BRT:PLA	No concerns	Low risk	No concerns	Major concerns	No concerns	No concerns	Low
CAF:ED	No concerns	Low risk	No concerns	Some concerns	Some concerns	No concerns	Moderate
CAF:PLA	No concerns	Low risk	No concerns	Some concerns	Some concerns	No concerns	Moderate
CAF:RHO	No concerns	Low risk	No concerns	Major concerns	No concerns	No concerns	Low

CHO:CRT	No concerns	Low risk	No concerns	Some concerns	Some concerns	No concerns	Moderate
CHO:PLA	Some concerns	Low risk	No concerns	Some concerns	Some concerns	No concerns	Moderate
CRT:PLA	Some concerns	Low risk	No concerns	No concerns	Some concerns	No concerns	Moderate
ED:PLA	Some concerns	Low risk	No concerns	Some concerns	Some concerns	No concerns	Moderate
HMB:PLA	No concerns	Low risk	No concerns	Some concerns	Some concerns	No concerns	Moderate
LC:PLA	Some concerns	Low risk	No concerns	Major concerns	No concerns	No concerns	Low
MG:PLA	No concerns	Low risk	No concerns	Major concerns	No concerns	No concerns	Low
PBC:PLA	No concerns	Low risk	No concerns	Major concerns	No concerns	No concerns	Low
PLA:PRT	Some concerns	Low risk	No concerns	Major concerns	No concerns	No concerns	Low
PLA:RHO	No concerns	Low risk	No concerns	Major concerns	No concerns	No concerns	Low
BA:BCAA	Some concerns	Low risk	No concerns	Major concerns	No concerns	No concerns	Low
BA:BRT	No concerns	Low risk	No concerns	Some concerns	Some concerns	No concerns	Moderate
BA:CAF	No concerns	Low risk	No concerns	Some concerns	Some concerns	No concerns	Moderate
BA:CHO	Some concerns	Low risk	No concerns	Some concerns	Some concerns	No concerns	Moderate
BA:CRT	Some concerns	Low risk	No concerns	Major concerns	No concerns	No concerns	Low
BA:ED	Some concerns	Low risk	No concerns	Some concerns	Some concerns	No concerns	Moderate
BA:HMB	No concerns	Low risk	No concerns	Major concerns	No concerns	No concerns	Low
BA:LC	Some concerns	Low risk	No concerns	Major concerns	No concerns	No concerns	Low
BA:MG	No concerns	Low risk	No concerns	Major concerns	No concerns	No concerns	Low
BA:PBC	No concerns	Low risk	No concerns	Major concerns	No concerns	No concerns	Low
BA:PRT	Some concerns	Low risk	No concerns	Major concerns	No concerns	No concerns	Low
BA:RHO	No concerns	Low risk	No concerns	Some concerns	Some concerns	No concerns	Moderate
BCAA:BRT	No concerns	Low risk	No concerns	Some concerns	Some concerns	No concerns	Moderate

BCAA:CAF	Some concerns	Low risk	No concerns	Some concerns	Some concerns	No concerns	Moderate
BCAA:CHO	Some concerns	Low risk	No concerns	Major concerns	No concerns	No concerns	Low
BCAA:ED	Some concerns	Low risk	No concerns	Some concerns	Some concerns	No concerns	Moderate
BCAA:HMB	No concerns	Low risk	No concerns	Major concerns	No concerns	No concerns	Low
BCAA:LC	Some concerns	Low risk	No concerns	Major concerns	No concerns	No concerns	Low
BCAA:MG	No concerns	Low risk	No concerns	Major concerns	No concerns	No concerns	Low
BCAA:PBC	No concerns	Low risk	No concerns	Major concerns	No concerns	No concerns	Low
BCAA:PRT	Some concerns	Low risk	No concerns	Major concerns	No concerns	No concerns	Low
BCAA:RHO	No concerns	Low risk	No concerns	Major concerns	No concerns	No concerns	Low
BRT:CAF	No concerns	Low risk	No concerns	Major concerns	No concerns	No concerns	Low
BRT:CHO	No concerns	Low risk	No concerns	Major concerns	No concerns	No concerns	Low
BRT:CRT	No concerns	Low risk	No concerns	Some concerns	Some concerns	No concerns	Moderate
BRT:ED	No concerns	Low risk	No concerns	Major concerns	No concerns	No concerns	Low
BRT:HMB	No concerns	Low risk	No concerns	Major concerns	No concerns	No concerns	Low
BRT:LC	Some concerns	Low risk	No concerns	Major concerns	No concerns	No concerns	Low
BRT:MG	No concerns	Low risk	No concerns	Major concerns	No concerns	No concerns	Low
BRT:PBC	No concerns	Low risk	No concerns	Major concerns	No concerns	No concerns	Low
BRT:PRT	Some concerns	Low risk	No concerns	Major concerns	No concerns	No concerns	Low
BRT:RHO	No concerns	Low risk	No concerns	Major concerns	No concerns	No concerns	Low
CAF:CHO	Some concerns	Low risk	No concerns	Major concerns	No concerns	No concerns	Low
CAF:CRT	Some concerns	Low risk	No concerns	Some concerns	Some concerns	No concerns	Moderate
CAF:HMB	No concerns	Low risk	No concerns	Major concerns	No concerns	No concerns	Low
CAF:LC	Some concerns	Low risk	No concerns	Major concerns	No concerns	No concerns	Low

CAF:MG	No concerns	Low risk	No concerns	Major concerns	No concerns	No concerns	Low
CAF:PBC	No concerns	Low risk	No concerns	Major concerns	No concerns	No concerns	Low
CAF:PRT	Some concerns	Low risk	No concerns	Major concerns	No concerns	No concerns	Low
CHO:ED	Some concerns	Low risk	No concerns	Major concerns	No concerns	No concerns	Low
CHO:HMB	No concerns	Low risk	No concerns	Major concerns	No concerns	No concerns	Low
CHO:LC	Some concerns	Low risk	No concerns	Major concerns	No concerns	No concerns	Low
CHO:MG	No concerns	Low risk	No concerns	Major concerns	No concerns	No concerns	Low
CHO:PBC	No concerns	Low risk	No concerns	Major concerns	No concerns	No concerns	Low
CHO:PRT	Some concerns	Low risk	No concerns	Major concerns	No concerns	No concerns	Low
CHO:RHO	No concerns	Low risk	No concerns	Major concerns	No concerns	No concerns	Low
CRT:ED	Some concerns	Low risk	No concerns	Some concerns	Some concerns	No concerns	Moderate
CRT:HMB	No concerns	Low risk	No concerns	Major concerns	No concerns	No concerns	Low
CRT:LC	Some concerns	Low risk	No concerns	Major concerns	No concerns	No concerns	Low
CRT:MG	No concerns	Low risk	No concerns	Major concerns	No concerns	No concerns	Low
CRT:PBC	No concerns	Low risk	No concerns	Major concerns	No concerns	No concerns	Low
CRT:PRT	Some concerns	Low risk	No concerns	Major concerns	No concerns	No concerns	Low
CRT:RHO	No concerns	Low risk	No concerns	Major concerns	No concerns	No concerns	Low
ED:HMB	No concerns	Low risk	No concerns	Some concerns	Some concerns	No concerns	Moderate
ED:LC	Some concerns	Low risk	No concerns	Major concerns	No concerns	No concerns	Low
ED:MG	No concerns	Low risk	No concerns	Major concerns	No concerns	No concerns	Low
ED:PBC	No concerns	Low risk	No concerns	Major concerns	No concerns	No concerns	Low
ED:PRT	Some concerns	Low risk	No concerns	Major concerns	No concerns	No concerns	Low
ED:RHO	No concerns	Low risk	No concerns	Major concerns	No concerns	No concerns	Low

HMB:LC	Some concerns	Low risk	No concerns	Major concerns	No concerns	No concerns	Low
HMB:MG	No concerns	Low risk	No concerns	Major concerns	No concerns	No concerns	Low
HMB:PBC	No concerns	Low risk	No concerns	Major concerns	No concerns	No concerns	Low
HMB:PRT	Some concerns	Low risk	No concerns	Major concerns	No concerns	No concerns	Low
HMB:RHO	No concerns	Low risk	No concerns	Major concerns	No concerns	No concerns	Low
LC:MG	Some concerns	Low risk	No concerns	Major concerns	No concerns	No concerns	Low
LC:PBC	Some concerns	Low risk	No concerns	Major concerns	No concerns	No concerns	Low
LC:PRT	Some concerns	Low risk	No concerns	Major concerns	No concerns	No concerns	Low
LC:RHO	No concerns	Low risk	No concerns	Major concerns	No concerns	No concerns	Low
MG:PBC	No concerns	Low risk	No concerns	Major concerns	No concerns	No concerns	Low
MG:PRT	Some concerns	Low risk	No concerns	Major concerns	No concerns	No concerns	Low
MG:RHO	No concerns	Low risk	No concerns	Major concerns	No concerns	No concerns	Low
PBC:PRT	Some concerns	Low risk	No concerns	Major concerns	No concerns	No concerns	Low
PBC:RHO	No concerns	Low risk	No concerns	Major concerns	No concerns	No concerns	Low
PRT:RHO	No concerns	Low risk	No concerns	Major concerns	No concerns	No concerns	Low

Table S9.2: CINeMA Results of Lower limb Peak Power

Comparison	Within-study bias	Reporting bias	Indirectness	Imprecision	Heterogeneity	Incoherence	Confidence rating
BA:PLA	No concerns	Low risk	No concerns	No concerns	Major concerns	No concerns	Low
CAF:CHO	No concerns	Low risk	No concerns	Major concerns	No concerns	No concerns	Low
CAF:ED	No concerns	Low risk	No concerns	Major concerns	No concerns	No concerns	Low
CAF:PLA	No concerns	Low risk	No concerns	Major concerns	No concerns	No concerns	Low
CHO:PLA	No concerns	Low risk	No concerns	Major concerns	No concerns	No concerns	Low
CRT:PLA	No concerns	Low risk	No concerns	Major concerns	No concerns	No concerns	Low

ED:PLA	No concerns	Low risk	No concerns	Major concerns	No concerns	No concerns	Low
HMB:PLA	No concerns	Low risk	No concerns	Major concerns	No concerns	No concerns	Low
MG:PLA	No concerns	Low risk	No concerns	Major concerns	No concerns	No concerns	Low
PBC:PLA	No concerns	Low risk	No concerns	Major concerns	No concerns	No concerns	Low
BA:CAF	No concerns	Low risk	No concerns	No concerns	Major concerns	No concerns	Low
BA:CHO	No concerns	Low risk	No concerns	Major concerns	No concerns	No concerns	Low
BA:CRT	No concerns	Low risk	No concerns	Some concerns	Some concerns	No concerns	Moderate
BA:ED	No concerns	Low risk	No concerns	Some concerns	Some concerns	No concerns	Moderate
BA:HMB	No concerns	Low risk	No concerns	No concerns	Major concerns	No concerns	Low
BA:MG	No concerns	Low risk	No concerns	Major concerns	No concerns	No concerns	Low
BA:PBC	No concerns	Low risk	No concerns	Major concerns	No concerns	No concerns	Low
CAF:CRT	No concerns	Low risk	No concerns	Major concerns	No concerns	No concerns	Low
CAF:HMB	No concerns	Low risk	No concerns	Major concerns	No concerns	No concerns	Low
CAF:MG	No concerns	Low risk	No concerns	Major concerns	No concerns	No concerns	Low
CAF:PBC	No concerns	Low risk	No concerns	Major concerns	No concerns	No concerns	Low
CHO:CRT	No concerns	Low risk	No concerns	Major concerns	No concerns	No concerns	Low
CHO:ED	No concerns	Low risk	No concerns	Major concerns	No concerns	No concerns	Low
CHO:HMB	No concerns	Low risk	No concerns	Major concerns	No concerns	No concerns	Low
CHO:MG	No concerns	Low risk	No concerns	Major concerns	No concerns	No concerns	Low
CHO:PBC	No concerns	Low risk	No concerns	Major concerns	No concerns	No concerns	Low
CRT:ED	No concerns	Low risk	No concerns	Major concerns	No concerns	No concerns	Low
CRT:HMB	No concerns	Low risk	No concerns	Major concerns	No concerns	No concerns	Low
CRT:MG	No concerns	Low risk	No concerns	Major concerns	No concerns	No concerns	Low

CRT:PBC	No concerns	Low risk	No concerns	Major concerns	No concerns	No concerns	Low
ED:HMB	No concerns	Low risk	No concerns	Major concerns	No concerns	No concerns	Low
ED:MG	No concerns	Low risk	No concerns	Major concerns	No concerns	No concerns	Low
ED:PBC	No concerns	Low risk	No concerns	Major concerns	No concerns	No concerns	Low
HMB:MG	No concerns	Low risk	No concerns	Major concerns	No concerns	No concerns	Low
HMB:PBC	No concerns	Low risk	No concerns	Major concerns	No concerns	No concerns	Low
MG:PBC	No concerns	Low risk	No concerns	Major concerns	No concerns	No concerns	Low

Table S9.3: CINeMA Results of Lower limb Mean Power

Comparison	Within-study bias	Reporting bias	Indirectness	Imprecision	Heterogeneity	Incoherence	Confidence rating
BA:PLA	No concerns	Low risk	No concerns	Major concerns	No concerns	No concerns	Low
CAF:CHO	No concerns	Low risk	No concerns	Major concerns	No concerns	No concerns	Low
CAF:PLA	No concerns	Low risk	No concerns	Major concerns	Some concerns	No concerns	Low
CHO:PLA	No concerns	Low risk	No concerns	Major concerns	No concerns	No concerns	Low
CRT:PLA	No concerns	Low risk	No concerns	Some concerns	Major concerns	No concerns	Moderate
GPJ:PLA	No concerns	Low risk	No concerns	Major concerns	No concerns	No concerns	Low
HMB:PLA	No concerns	Low risk	No concerns	No concerns	Major concerns	No concerns	Low
BA:CAF	No concerns	Low risk	No concerns	Major concerns	No concerns	No concerns	Low
BA:CHO	No concerns	Low risk	No concerns	Major concerns	No concerns	No concerns	Low
BA:CRT	No concerns	Low risk	No concerns	Major concerns	No concerns	No concerns	Low
BA:GPJ	No concerns	Low risk	No concerns	Major concerns	No concerns	No concerns	Low

BA:HMB	No concerns	Low risk	No concerns	Major concerns	No concerns	No concerns	Low
CAF:CRT	No concerns	Low risk	No concerns	Major concerns	No concerns	No concerns	Low
CAF:GPJ	No concerns	Low risk	No concerns	Major concerns	No concerns	No concerns	Low
CAF:HMB	No concerns	Low risk	No concerns	Some concerns	Some concerns	No concerns	Moderate
CHO:CRT	No concerns	Low risk	No concerns	Major concerns	No concerns	No concerns	Low
CHO:GPJ	No concerns	Low risk	No concerns	Major concerns	No concerns	No concerns	Low
CHO:HMB	No concerns	Low risk	No concerns	Some concerns	Some concerns	No concerns	Moderate
CRT:GPJ	No concerns	Low risk	No concerns	Major concerns	No concerns	No concerns	Low
CRT:HMB	No concerns	Low risk	No concerns	Some concerns	Some concerns	No concerns	Moderate
GPJ:HMB	No concerns	Low risk	No concerns	Major concerns	No concerns	No concerns	Low

Appendix 10: Sensitivity analyses

Sensitivity analysis was performed by adding trials comparing the effects of nutritional supplements on performance in other sports similar to volleyball.Sensitivity analysis was performed after excluding trials with high risk of bias.

Table S10.1: Baseline characteristics of included studies supplemented in sensitivity analyses

Study	Study Design	Participants Level	Simple Size(N)	Age(SD)	height(SD)	weight(SD)	Sex	Study Period	Ingestion Time	Intervention	Comparator(s)	Performance Test	side effects
Turcu et al., 2022 /	Double-blind, randomized, placebo-control	non-elite	BA: 10; PLA:	23 ± 0.6	185.3 ± 5.4	78.3 ± 4.8	Male	8 weeks	1 h prior to training	β-alanine capsules 6.4 g/day	Maltodextrin capsules	VJ	no tingling, GI

Romania [105]	led trial		10						+ morning dose				discomfort, weight gain
Zajac et al., 2003 / Poland [106]	Randomized, placebo-controlled, 4-arm parallel-group design	non-elite	PLA: 13; CRT: 12; HMB: 12	25.6 ± 5.64	193.2± 7.18	92.7 ± 8.69	Male	30 days	timing not strictly specified, but ingested with CHO solution	creatine monohydrate, HMB	Placebo	PP	NA
Salleh et al., 2021 / Malaysia [107]	Randomized, placebo-controlled, double-blind	non-elite	PBC: 15; PLA: 15	19.5 ± 1; 19.9 ± 1.3	1.66 ±0.45; 1.69 ± 0.61	63.9 ± 8.3; 67.7 ± 7.1	Male and Female	6 weeks	Daily ingestion	probiotic drink(80 mL probiotic drink)	placebo drink (orange juice, 200 mL)	VJ	NA
Abian et al., 2015 / Spain [108]	Randomized, double-blind, placebo-controlled crossover	elite	16	25.4 ± 7.3	174.1 ± 5.8	71.8 ± 7.9	Male	separated by 1 week	60 min	caffeinated energy drink (3 mg/kg caffeine,)	placebo energy drink (same without caffeine)	VJ	some reported nervousness, GI discomfort, sleep disturbanc

													e
Rosas et al., 2017 / Chile, Argentina, Spain, etc. [109]	Double-blind, randomized, placebo-controlled trial	non-elite	BA: 8; BA: 8	24.3± 2.5; 22.8± 2.1	1.62 ± 0.05; 1.64 ± 0.08	58.1 ± 6.3; 61.1 ± 8.3	female	6 weeks	6 weeks	plyometric training + β-alanine capsules (4.8 g/day)	plyometric training + placebo	VJ, PP	mild paresthesia

Abbreviations: PLA, Placebo; BA, β-Alanine; CHO, Carbohydrate; CRT, Creatine; PBC, Probiotics; HMB, β-Hydroxy-β-Methylbutyrate; CAF, Caffeine; ED, Energy Drink; VJ, vertical jump; PP, lower limb peak power; MP, lower limb mean power

References

105. Turcu, I.; Oancea, B.; Chicomban, M.; Simion, G.; Simon, S.; Negriu Tiuca, C.I.; Ordean, M.N.; Petrovici, A.G.; Nicolescu Șeușan, N.A.; Hăisan, P.L.; et al. Effect of 8-week β-alanine supplementation on CRP, IL-6, body composition, and bio-motor abilities in elite male basketball players. Int. J. Environ. Res. Public Health **2022**, *19*, 13700. https://doi.org/10.3390/ijerph192013700

106. Zajac, A.; Waskiewicz, Z.; Poprzecki, S.; Cholewa, J. Effects of creatine and HMB supplementation on anaerobic power and body composition in basketball players. J. Hum. Kinet. **2003**, *10*, 95–108.

107. Salleh, R.M.; Kuan, G.; Aziz, M.N.A.; Rahim, M.R.A.; Rahayu, T.; Sulaiman, S.; Kusuma, D.W.Y.; Adikari, A.M.G.C.P.; Razam, M.S.M.; Radhakrishnan, A.K.; et al. Effects of probiotics on anxiety, stress, mood and fitness of badminton players. Nutrients **2021**, *13*, 1783. https://doi.org/10.3390/nu13061783

108. Abian, P.; Del Coso, J.; Salinero, J.J.; Gallo-Salazar, C.; Areces, F.; Ruiz-Vicente, D.; Lara, B.; Soriano, L.; Muñoz, V.; Abian-Vicen, J. The ingestion of a caffeinated energy drink improves jump performance and activity patterns in elite badminton players. J. Sports Sci. **2015**, *33*, 1042–1050. https://doi.org/10.1080/02640414.2014.981849.

109. Rosas, F.; Ramírez-Campillo, R.; Martínez, C.; Caniuqueo, A.; Cañas-Jamet, R.; McCrudden, E.; Meylan, C.; Moran, J.; Nakamura, F.Y.; Pereira, L.A.; et al. Effects of plyometric training and beta-alanine supplementation on maximal-intensity exercise and endurance in female soccer players. J. Hum. Kinet. **2017**, *58*, 99-109. https://doi.org/10.1515/hukin-2017-0072.

Table S10.2: Sensitivity analyses of Vertical Jump outcome

Nutritional Supplement	Vertcial Jump	
	Main estimate	Sensitivity estimate
BA	4.6 (1.2, 7.8)	3.5 (1.2, 5.9)
HMB	6.1 (−2.1, 14)	6.0 (−1.8, 14)
CRT	3.7 (0.57, 6.9)	3.2 (0.79, 5.7)
BCAA	3.2 (−0.45, 7.1)	3.1 (−0.44, 6.6)
CAF	2.1 (0.06, 4.1)	2.1 (0.15, 3.9)
PBC	2.7 (−5.4, 11)	1.6 (−2.5, 5.9)
MG	1.9 (−4.0, 7.9)	1.9 (−3.6, 7.5)
RHO	−1.5 (−6.3, 3.3)	−1.5 (−6.0, 2.9)

PRT	−0.98 (−6.4, 4.5)	−1.0 (−6.1, 4.1)
BRT	0.59 (−3.8, 4.9)	0.67 (−3.4, 4.8)
CHO	1.5 (−2.7, 5.8)	1.3 (−2.6, 5.3)
ED	0.5 (−3.1, 4.1)	0.44 (−2.7, 3.5)
LC	1.9 (−4.9, 8.6)	1.9 (−4.7, 8.3)

Table S10.3: Sensitivity analyses of Lower limb peak power outcome

Nutritional Supplement	Lower limb peak power	
	Main estimate	Sensitivity estimate
BA	1.1 (0.2, 2.0)	1.4 (-0.56, 3.3)
CAF	0.36 (-0.16, 0.96)	0.39 (-0.59, 1.4)
CHO	0.062 (-0.95, 1.2)	0.12 (-1.9, 2.0)
CRT	0.25 (-0.77, 1.3)	0.63 (-0.35, 2.0)
ED	0.1 (-0.58, 0.85)	0.12 (-1.2, 1.4)
HMB	0.51 (-0.41, 1.4)	0.69 (-0.51, 2.0)
MG	-0.35 (-1.4, 0.69)	-0.32 (-2.5, 1.8)
PBC	-0.01 (-1.2, 1.2)	-0.03 (-2.2, 2.1)

Table S10.4: Sensitivity analyses of Lower limb mean power outcome

Nutritional Supplement	Lower limb mean power	
	Main estimate	Sensitivity estimate
BA	0.21 (−0.98, 1.4)	0.55 (−0.38, 1.5)
CAF	0.18 (−0.78, 1.1)	0.18 (−0.98, 1.4)

CHO	−0.05 (−1.3, 1.2)	−0.049 (−1.6, 1.5)
CRT	0.29 (−0.87, 1.5)	0.30 (−0.78, 1.4)
ED	0.04 (−1.2, 1.2)	0.04 (−1.6, 1.6)
GPJ	0.39 (−0.86, 1.6)	0.39 (−1.2, 2.0)
HMB	0.97 (−0.30, 2.2)	0.93 (−0.64, 2.6)

Appendix 11 – Meta-regression analyses results

Table S11.1 Vertical Jump

Variables	Coefficient	95%CrI
Age	1.25	-2.01, 4.19
Competitive level	-0.91	-3.65, 2.01

Table S11.2 Lower limb Peak Power

Variables	Coefficient	95%CrI
Age	-0.28	-1.26, 0.73
Competitive level	-0.55	-1.45, 0.33

Table S11.3 Lower limb Mean Power

Variables	Coefficient	95%CrI
Age	-0.13	-1.65, 1.31
Competitive level	-0.13	-1.65, 1.33

Appendix 12: Subgroup analysis of different supplement of physical performance on Age

Table S12.1 League table of youth volleyball athletes
(A)Vertical Jump

BA	0.57 (-4.04, 5.69)	-2.17 (-62.3, 59.79)	-0.73 (-4.61, 3.27)	-0.88 (-46.42, 51.29)	0.98 (-3.32, 5.67)	-2.18 (-7.29, 3.03)	3.71 (-5.52, 13.06)	-1.35 (-146.41, 38.1)	-0.11 (-38.98, 45.48)	0.88 (-50.47, 149.13)	-2.55 (-5.83, 0.89)	-1.17 (-49.17, 139.22)	-1.02 (-23.19, 29.96)
-0.57 (-5.69, 4.04)	BCAA	-2.82 (-62.9, 59.12)	-1.32 (-5.53, 2.59)	-1.57 (-47.06, 51.08)	0.41 (-3.7, 4.42)	-2.76 (-8.16, 2.31)	3.08 (-6.32, 12.41)	-1.96 (-147.17, 37.31)	-0.71 (-39.65, 44.72)	0.26 (-51.1, 149.36)	-3.13 (-6.79, 0.23)	-1.76 (-49.9, 138.85)	-1.68 (-23.82, 29.14)
2.17 (-59.79, 62.3)	2.82 (-59.12, 62.9)	BRT	1.47 (-60.31, 61.6)	2.99 (-72.3, 69.03)	3.19 (-58.69, 63.17)	0.02 (-62.06, 60.02)	5.89 (-57.52, 66.16)	-1.13 (-141.3, 73.85)	2.36 (-61.75, 76.94)	3.95 (-72.82, 149.82)	-0.37 (-62.09, 59.72)	4.02 (-74.59, 138.68)	2.07 (-66.29, 64.38)
0.73 (-3.27, 4.61)	1.32 (-2.59, 5.53)	-1.47 (-61.6, 60.31)	CAF	-0.23 (-45.68, 52.1)	1.73 (-1.78, 5.41)	-1.44 (-5.23, 2.33)	4.44 (-4.44, 13.37)	-0.59 (-145.8, 38.73)	0.62 (-38.26, 45.92)	1.61 (-49.51, 150.27)	-1.81 (-3.86, 0.25)	-0.47 (-48.57, 140.02)	-0.33 (-22.26, 30.48)
0.88 (-51.29, 46.42)	1.57 (-51.08, 47.06)	-2.99 (-69.03, 72.3)	0.23 (-52.1, 45.68)	CHO	1.95 (-50.25, 47.3)	-1.25 (-53.69, 43.88)	4.73 (-48.22, 50.8)	-0.69 (-158.54, 58.64)	0.73 (-66.72, 65.9)	1.59 (-79.43, 165.74)	-1.59 (-53.86, 43.94)	-0.82 (-67.73, 173.05)	0.86 (-50.83, 43.87)
-0.98 (-5.67, 3.32)	-0.41 (-4.42, 3.7)	-3.19 (-63.17, 58.69)	-1.73 (-5.41, 1.78)	-1.95 (-47.3, 50.25)	CRT	-3.17 (-8.12, 1.59)	2.69 (-6.45, 11.88)	-2.38 (-147.79, 36.96)	-1.1 (-40.18, 44.34)	-0.13 (-51.44, 148.7)	-3.53 (-6.57, -0.69)	-2.21 (-50.3, 138.3)	-2.06 (-24.23, 28.71)
2.18 (-3.03, 7.29)	2.76 (-2.31, 8.16)	-0.02 (-60.02, 62.06)	1.44 (-2.33, 5.23)	1.25 (-43.88, 53.69)	3.17 (-1.59, 8.12)	ED	5.89 (-3.6, 15.36)	0.81 (-144.12, 40.09)	2.08 (-36.94, 47.49)	3.04 (-48.34, 152.08)	-0.36 (-4.25, 3.53)	1.05 (-47.19, 141.48)	1.12 (-21.16, 32.12)
-3.71 (-13.06, 5.52)	-3.08 (-12.41, 6.32)	-5.89 (-66.16, 57.52)	-4.44 (-13.37, 4.44)	-4.73 (-50.8, 48.22)	-2.69 (-11.88, 6.45)	-5.89 (-15.36, 3.6)	HMB	-5.15 (-149.77, 34.89)	-3.88 (-43.41, 42.25)	-2.75 (-54.6, 145.39)	-6.25 (-14.93, 2.38)	-4.79 (-53.4, 135.75)	-4.66 (-28.06, 26.6)
1.35 (-38.1,	1.96 (-37.31,	1.13 (-73.85,	0.59 (-38.73,	0.69 (-58.64,	2.38 (-36.96,	-0.81 (-40.09,	5.15 (-34.89,	LC	2.62 (-49.09,	3.92 (-47.71,	-1.23 (-40.43,	3.3 (-56.11,	1.3 (-45.51,

146.41)	147.17)	141.3)	145.8)	158.54)	147.79)	144.12)	149.77)		139.7)	181.26)	144.06)	154.88)	148.86)
0.11 (-45.48, 38.98)	0.71 (-44.72, 39.65)	-2.36 (-76.94, 61.75)	-0.62 (-45.92, 38.26)	-0.73 (-65.9, 66.72)	1.1 (-44.34, 40.18)	-2.08 (-47.49, 36.94)	3.88 (-42.25, 43.41)	-2.62 (-139.7, 49.09)	MG	1.71 (-77.27, 144.88)	-2.43 (-47.81, 36.41)	-1.01 (-55.13, 137.35)	-0.69 (-44.54, 43.7)
-0.88 (-149.13, 50.47)	-0.26 (-149.36, 51.1)	-3.95 (-149.82, 72.82)	-1.61 (-150.27, 49.51)	-1.59 (-165.74, 79.43)	0.13 (-148.7, 51.44)	-3.04 (-152.08, 48.34)	2.75 (-145.39, 54.6)	-3.92 (-181.26, 47.71)	-1.71 (-144.88, 77.27)	PBC	-3.41 (-152.32, 47.82)	-1.55 (-90.4, 114.36)	-2 (-149.85, 58.14)
2.55 (-0.89, 5.83)	3.13 (-0.23, 6.79)	0.37 (-59.72, 62.09)	1.81 (-0.25, 3.86)	1.59 (-43.94, 53.86)	3.53 (0.69, 6.57)	0.36 (-3.53, 4.25)	6.25 (-2.38, 14.93)	1.23 (-144.06, 40.43)	2.43 (-36.41, 47.81)	3.41 (-47.82, 152.32)	PLA	1.34 (-46.69, 141.79)	1.5 (-20.53, 32.4)
1.17 (-139.22, 49.17)	1.76 (-138.85, 49.9)	-4.02 (-138.68, 74.59)	0.47 (-140.02, 48.57)	0.82 (-173.05, 67.73)	2.21 (-138.3, 50.3)	-1.05 (-141.48, 47.19)	4.79 (-135.75, 53.4)	-3.3 (-154.88, 56.11)	1.01 (-137.35, 55.13)	1.55 (-114.36, 90.4)	-1.34 (-141.79, 46.69)	PRT	0.03 (-144.75, 53.94)
1.02 (-29.96, 23.19)	1.68 (-29.14, 23.82)	-2.07 (-64.38, 66.29)	0.33 (-30.48, 22.26)	-0.86 (-43.87, 50.83)	2.06 (-28.71, 24.23)	-1.12 (-32.12, 21.16)	4.66 (-26.6, 28.06)	-1.3 (-148.86, 45.51)	0.69 (-43.7, 44.54)	2 (-58.14, 149.85)	-1.5 (-32.4, 20.53)	-0.03 (-53.94, 144.75)	RHO

(B)Lower-Limb Peak Power

BA	-0.75 (-12.81, 15.69)	-1.2 (-15.46, 15.03)	-0.8 (-16.88, 20.59)	-0.93 (-19.83, 19.69)	-0.6 (-12.66, 15.82)	-1.01 (-13.27, 34.59)	-1.08 (-15.16, 15.69)	-1.08 (-13.15, 15.31)
0.75 (-15.69, 12.81)	CAF	-0.37 (-9.5, 5.12)	-0.03 (-12.26, 13.34)	-0.21 (-8.05, 13.13)	0.15 (-1.05, 1.36)	-0.48 (-7.81, 40.18)	-0.32 (-8.59, 8.23)	-0.32 (-0.91, 0.25)
1.2 (-15.03, 15.46)	0.37 (-5.12, 9.5)	CHO	0.48 (-11.27, 13.3)	0.23 (-14.18, 16.07)	0.54 (-5.13, 9.65)	0.13 (-8.97, 41.1)	0.09 (-9.92, 12.45)	0.05 (-5.44, 9.14)
0.8 (-20.59,	0.03 (-13.34,	-0.48 (-13.3,	CRT	-0.15 (-19.68,	0.18 (-13.23,	-0.3 (-15.04,	-0.32 (-16.78,	-0.29 (-13.7,

16.88)	12.26)	11.27)		17.13)	12.29)	40.21)	14)	11.92)
0.93 (-19.69, 19.83)	0.21 (-13.13, 8.05)	-0.23 (-16.07, 14.18)	0.15 (-17.13, 19.68)	ED	0.35 (-12.95, 8.32)	-0.21 (-14.55, 40.39)	-0.17 (-17.14, 15.71)	-0.11 (-13.42, 7.72)
0.6 (-15.82, 12.66)	-0.15 (-1.36, 1.05)	-0.54 (-9.65, 5.13)	-0.18 (-12.29, 13.23)	-0.35 (-8.32, 12.95)	HMB	-0.62 (-8.02, 40.02)	-0.48 (-8.81, 8.07)	-0.48 (-1.55, 0.58)
1.01 (-34.59, 13.27)	0.48 (-40.18, 7.81)	-0.13 (-41.1, 8.97)	0.3 (-40.21, 15.04)	0.21 (-40.39, 14.55)	0.62 (-40.02, 8.02)	MG	-0.03 (-41.12, 10.67)	0.17 (-40.5, 7.49)
1.08 (-15.69, 15.16)	0.32 (-8.23, 8.59)	-0.09 (-12.45, 9.92)	0.32 (-14, 16.78)	0.17 (-15.71, 17.14)	0.48 (-8.07, 8.81)	0.03 (-10.67, 41.12)	PBC	0.01 (-8.56, 8.24)
1.08 (-15.31, 13.15)	0.32 (-0.25, 0.91)	-0.05 (-9.14, 5.44)	0.29 (-11.92, 13.7)	0.11 (-7.72, 13.42)	0.48 (-0.58, 1.55)	-0.17 (-7.49, 40.5)	0 .01 (-8.24, 8.56)	PLA

(C)Lower limb Mean Power

BA	-0.08 (-5.99, 5.18)	-0.37 (-7.83, 6.03)	0.08 (-8.1, 7.68)	-0.21 (-7.34, 7.31)	0.11 (-8.35, 9.24)	0.72 (-5.97, 7.24)	-0.24 (-6.13, 4.94)
0.08 (-5.18, 5.99)	CAF	-0.27 (-3.81, 2.32)	0.17 (-3.21, 4.47)	-0.11 (-3.96, 4.24)	0.2 (-5.24, 3.95)	0.78 (-2.37, 3.88)	-0.16 (-1.16, 0.82)
0.37 (-6.03, 7.83)	0.27 (-2.32, 3.81)	CHO	0.45 (-4.16, 6.34)	0.16 (-4.44, 5.88)	0.47 (-5.46, 6.3)	1.07 (-3.25, 5.76)	0.11 (-2.5, 3.64)
-0.08 (-7.68, 8.1)	-0.17 (-4.47, 3.21)	-0.45 (-6.34, 4.16)	CRT	-0.29 (-5.93, 5.64)	0.03 (-7.31, 6.12)	0.61 (-4.82, 5.44)	-0.33 (-4.53, 2.95)
0.21 (-7.31, 7.34)	0.11 (-4.24, 3.96)	-0.16 (-5.88, 4.44)	0.29 (-5.64, 5.93)	ED	0.3 (-6.66, 6.23)	0.89 (-4.57, 5.86)	-0.04 (-4.35, 3.7)
-0.11 (-9.24, 8.35)	-0.2 (-3.95, 5.24)	-0.47 (-6.3, 5.46)	-0.03 (-6.12, 7.31)	-0.3 (-6.23, 6.66)	GPJ	0.6 (-4.91, 6.93)	-0.37 (-3.98, 5.04)
-0.72 (-7.24, 5.97)	-0.78 (-3.88, 2.37)	-1.07 (-5.76, 3.25)	-0.61 (-5.44, 4.82)	-0.89 (-5.86, 4.57)	-0.6 (-6.93, 4.91)	HMB	-0.95 (-3.89, 2.08)
0.24 (-4.94, 6.13)	0.16 (-0.82, 1.16)	-0.11 (-3.64, 2.5)	0.33 (-2.95, 4.53)	0.04 (-3.7, 4.35)	0.37 (-5.04, 3.98)	0.95 (-2.08, 3.89)	PLA

(

Table S12.2 League table of adult volleyball athletes

(A)Vertical Jump

BA	3.31 (-3.01, 10.19)	0.74 (-98.42, 102.78)	2.9 (-2.59, 8.26)	1.92 (-4.42, 8.47)	4.04 (-2.12, 10.61)	1.07 (-6.76, 8.72)	7.28 (-6.02, 20.81)	2.49 (-5.56, 10.47)	2.52 (-4.82, 9.76)	3.27 (-5.82, 12.27)	0.6 (-4.49, 5.56)	1.6 (-5.33, 8.51)	2.2 (-4.42, 8.69)
-3.31 (-10.19, 3.01)	BCAA	-2.6 (-101.32, 99.21)	-0.43 (-5.47, 4.04)	-1.4 (-6.95, 3.83)	0.72 (-3.98, 5.33)	-2.24 (-9.73, 4.6)	3.89 (-9.18, 17.09)	-0.84 (-8.69, 6.51)	-0.8 (-7.94, 5.73)	-0.09 (-8.9, 8.42)	-2.73 (-7.31, 1.28)	-1.72 (-8.47, 4.38)	-1.12 (-7.5, 4.57)
-0.74 (-102.78, 98.42)	2.6 (-99.21, 101.32)	BRT	2.14 (-99.67, 100.92)	1.19 (-100.76, 100.08)	3.31 (-98.63, 102.23)	0.35 (-101.86, 99.25)	6.61 (-97.38, 105.76)	1.68 (-100.38, 100.96)	1.74 (-100.33, 100.6)	2.53 (-99.32, 101.56)	-0.17 (-101.92, 98.73)	0.87 (-101.04 , 99.7)	1.45 (-100.47, 100.15)
-2.9 (-8.26, 2.59)	0.43 (-4.04, 5.47)	-2.14 (-100.92, 99.67)	CAF	-0.99 (-5.31, 3.69)	1.15 (-2.98, 5.8)	-1.81 (-7.3, 3.62)	4.37 (-8.09, 17.11)	-0.4 (-6.98, 6.16)	-0.38 (-6.06, 5.32)	0.37 (-7.42, 8.15)	-2.3 (-4.32, -0.27)	-1.3 (-6.47, 3.92)	-0.7 (-4.96, 3.51)
-1.92 (-8.47, 4.42)	1.4 (-3.83, 6.95)	-1.19 (-100.08, 100.76)	0.99 (-3.69, 5.31)	CHO	2.12 (-1.92, 6.42)	-0.85 (-8.04, 5.98)	5.33 (-7.62, 18.53)	0.59 (-7.05, 7.84)	0.61 (-6.21, 7.13)	1.34 (-7.28, 9.76)	-1.31 (-5.5, 2.53)	-0.3 (-6.76, 5.73)	0.29 (-5.77, 5.93)
-4.04 (-10.61, 2.12)	-0.72 (-5.33, 3.98)	-3.31 (-102.23, 98.63)	-1.15 (-5.8, 2.98)	-2.12 (-6.42, 1.92)	CRT	-2.96 (-10.21, 3.72)	3.18 (-9.78, 16.35)	-1.56 (-9.18, 5.59)	-1.51 (-8.38, 4.81)	-0.81 (-9.4, 7.57)	-3.45 (-7.6, 0.21)	-2.44 (-8.89, 3.45)	-1.83 (-7.9, 3.58)
-1.07 (-8.72, 6.76)	2.24 (-4.6, 9.73)	-0.35 (-99.25, 101.86)	1.81 (-3.62, 7.3)	0.85 (-5.98, 8.04)	2.96 (-3.72, 10.21)	ED	6.18 (-7.39, 20.08)	1.42 (-7.07, 9.95)	1.44 (-6.39, 9.32)	2.18 (-7.25, 11.79)	-0.49 (-6.24, 5.37)	0.52 (-6.95, 8.08)	1.12 (-5.77, 8)
-7.28 (-20.81, 6.02)	-3.89 (-17.09, 9.18)	-6.61 (-105.76, 97.38)	-4.37 (-17.11, 8.09)	-5.33 (-18.53, 7.62)	-3.18 (-16.35, 9.78)	-6.18 (-20.08, 7.39)	HMB	-4.78 (-18.81, 9.05)	-4.76 (-18.38, 8.65)	-4.02 (-18.65, 10.43)	-6.67 (-19.27, 5.6)	-5.68 (-19.13, 7.47)	-5.09 (-18.29, 7.88)

-2.49 (-10.47, 5.56)	0.84 (-6.51, 8.69)	-1.68 (-100.96, 100.38)	0.4 (-6.16, 6.98)	-0.59 (-7.84, 7.05)	1.56 (-5.59, 9.18)	-1.42 (-9.95, 7.07)	4.78 (-9.05, 18.81)	LC	0.02 (-8.16, 8.26)	0.77 (-8.98, 10.59)	-1.9 (-8.13, 4.37)	-0.89 (-8.77, 6.94)	-0.3 (-7.84, 7.22)
-2.52 (-9.76, 4.82)	0.8 (-5.73, 7.94)	-1.74 (-100.6, 100.33)	0.38 (-5.32, 6.06)	-0.61 (-7.13, 6.21)	1.51 (-4.81, 8.38)	-1.44 (-9.32, 6.39)	4.76 (-8.65, 18.38)	-0.02 (-8.26, 8.16)	MG	0.74 (-8.43, 9.96)	-1.92 (-7.22, 3.41)	-0.92 (-8.03, 6.2)	-0.32 (-7.14, 6.46)
-3.27 (-12.27, 5.82)	0.09 (-8.42, 8.9)	-2.53 (-101.56, 99.32)	-0.37 (-8.15, 7.42)	-1.34 (-9.76, 7.28)	0.81 (-7.57, 9.4)	-2.18 (-11.79, 7.25)	4.02 (-10.43, 18.65)	-0.77 (-10.59, 8.98)	-0.74 (-9.96, 8.43)	PBC	-2.67 (-10.21, 4.87)	-1.66 (-10.6, 7.17)	-1.08 (-9.71, 7.53)
-0.6 (-5.56, 4.49)	2.73 (-1.28, 7.31)	0.17 (-98.73, 101.92)	2.3 (0.27, 4.32)	1.31 (-2.53, 5.5)	3.45 (-0.21, 7.6)	0.49 (-5.37, 6.24)	6.67 (-5.6, 19.27)	1.9 (-4.37, 8.13)	1.92 (-3.41, 7.22)	2.67 (-4.87, 10.21)	PLA	1 (-3.76, 5.77)	1.6 (-2.65, 5.81)
-1.6 (-8.51, 5.33)	1.72 (-4.38, 8.47)	-0.87 (-99.7, 101.04)	1.3 (-3.92, 6.47)	0.3 (-5.73, 6.76)	2.44 (-3.45, 8.89)	-0.52 (-8.08, 6.95)	5.68 (-7.47, 19.13)	0.89 (-6.94, 8.77)	0.92 (-6.2, 8.03)	1.66 (-7.17, 10.6)	-1 (-5.77, 3.76)	PRT	0.59 (-5.79, 6.95)
-2.2 (-8.69, 4.42)	1.12 (-4.57, 7.5)	-1.45 (-100.15, 100.47)	0.7 (-3.51, 4.96)	-0.29 (-5.93, 5.77)	1.83 (-3.58, 7.9)	-1.12 (-8, 5.77)	5.09 (-7.88, 18.29)	0.3 (-7.22, 7.84)	0.32 (-6.46, 7.14)	1.08 (-7.53, 9.71)	-1.6 (-5.81, 2.65)	-0.59 (-6.95, 5.79)	RHO

(B)Lower limb Peak Power

BA	-0.55 (-28.67, 37.71)	-1.28 (-34.45, 36.54)	-0.82 (-28.81, 37.54)	-0.76 (-44.65, 47.26)	-0.6 (-28.64, 37.73)	-1.41 (-29.5, 36.91)	-1.04 (-33.68, 38.01)	-1.07 (-29.15, 37.2)
0.55 (-37.71, 28.67)	CAF	-0.6 (-21.82, 12.11)	-0.28 (-1.79, 1.22)	-0.3 (-18.22, 30.94)	-0.05 (-1.63, 1.53)	-0.87 (-2.38, 0.65)	-0.49 (-19.61, 19.43)	-0.52 (-1.33, 0.28)
1.28 (-36.54, 34.45)	0.6 (-12.11, 21.82)	CHO	0.33 (-12.39, 21.54)	0.44 (-32.96, 37.29)	0.57 (-12.25, 21.77)	-0.25 (-13, 20.94)	0.22 (-23.08, 28.99)	0.08 (-12.61, 21.27)

0.82 (-37.54, 28.81)	0.28 (-1.22, 1.79)	-0.33 (-21.54, 12.39)	CRT	-0.01 (-18.01, 31.05)	0.23 (-1.63, 2.1)	-0.58 (-2.4, 1.22)	-0.2 (-19.34, 19.69)	-0.24 (-1.52, 1.05)
0.76 (-47.26, 44.65)	0.3 (-30.94, 18.22)	-0.44 (-37.29, 32.96)	0.01 (-31.05, 18.01)	ED	0.24 (-30.96, 18.32)	-0.56 (-31.66, 17.43)	-0.29 (-39.81, 36.72)	-0.21 (-31.37, 17.69)
0.6 (-37.73, 28.64)	0.05 (-1.53, 1.63)	-0.57 (-21.77, 12.25)	-0.23 (-2.1, 1.63)	-0.24 (-18.32, 30.96)	HMB	-0.81 (-2.68, 1.04)	-0.44 (-19.61, 19.41)	-0.47 (-1.83, 0.89)
1.41 (-36.91, 29.5)	0.87 (-0.65, 2.38)	0.25 (-20.94, 13)	0.58 (-1.22, 2.4)	0.56 (-17.43, 31.66)	0.81 (-1.04, 2.68)	MG	0.38 (-18.81, 20.28)	0.34 (-0.93, 1.63)
1.04 (-38.01, 33.68)	0.49 (-19.43, 19.61)	-0.22 (-28.99, 23.08)	0.2 (-19.69, 19.34)	0.29 (-36.72, 39.81)	0.44 (-19.41, 19.61)	-0.38 (-20.28, 18.81)	PBC	-0.03 (-19.9, 19.14)
1.07 (-37.2, 29.15)	0.52 (-0.28, 1.33)	-0.08 (-21.27, 12.61)	0.24 (-1.05, 1.52)	0.21 (-17.69, 31.37)	0.47 (-0.89, 1.83)	-0.34 (-1.63, 0.93)	0.03 (-19.14, 19.9)	PLA

(C)Lower limb Mean Power

BA	0.01 (-1.82, 1.85)	-0.34 (-8.49, 5.29)	0.12 (-7.37, 9.84)	-0.18 (-2.03, 1.68)	0.12 (-12.41, 8.35)	0.73 (-6.12, 7.38)	-0.22 (-1.59, 1.16)
-0.01 (-1.85, 1.82)	CAF	-0.35 (-8.47, 5.25)	0.1 (-7.29, 9.83)	-0.19 (-1.94, 1.55)	0.11 (-12.36, 8.33)	0.71 (-6.11, 7.37)	-0.23 (-1.47, 0.99)
0.34 (-5.29, 8.49)	0.35 (-5.25, 8.47)	CHO	0.49 (-9.65, 14.05)	0.17 (-5.49, 8.23)	0.46 (-13.12, 13.66)	1.09 (-8.34, 11.64)	0.12 (-5.42, 8.19)
-0.12 (-9.84, 7.37)	-0.1 (-9.83, 7.29)	-0.49 (-14.05, 9.65)	CRT	-0.29 (-10.02, 7.15)	-0.02 (-16.96, 13.8)	0.59 (-11.82, 11.43)	-0.33 (-9.99, 7.07)
0.18 (-1.68, 2.03)	0.19 (-1.55, 1.94)	-0.17 (-8.23, 5.49)	0.29 (-7.15, 10.02)	ED	0.3 (-12.19, 8.45)	0.91 (-5.9, 7.55)	-0.04 (-1.29, 1.22)
-0.12 (-8.35,	-0.11 (-8.33,	-0.46 (-13.66,	0.02 (-13.8, 16.96)	-0.3 (-8.45,	GPJ	0.65 (-11.71,	-0.35 (-8.45,

12.41)	12.36)	13.12)		12.19)		15.17)	12.19)
-0.73 (-7.38, 6.12)	-0.71 (-7.37, 6.11)	-1.09 (-11.64, 8.34)	-0.59 (-11.43, 11.82)	-0.91 (-7.55, 5.9)	-0.65 (-15.17, 11.71)	HMB	-0.94 (-7.53, 5.83)
0.22 (-1.16, 1.59)	0.23 (-0.99, 1.47)	-0.12 (-8.19, 5.42)	0.33 (-7.07, 9.99)	0.04 (-1.22, 1.29)	0.35 (-12.19, 8.45)	0.94 (-5.83, 7.53)	PLA

Appendix 13: Subgroup analysis of different supplement of physical performance on competitive level

Table S13.1 League table of elite volleyball athletes

(A)Vertical Jump

BA	-0.38 (-6.36, 6.08)	-3.39 (-9.99, 3.33)	-1.76 (-6.55, 3.09)	-2.88 (-120.91, 29.22)	-0.18 (-5.57, 5.58)	-3.35 (-9.2, 2.57)	4.82 (-58.9, 331.75)	-3.78 (-76.23, 48.52)	-4.78 (-97.36, 38.69)	-1.89 (-61.65, 80.2)	-3.86 (-8.04, 0.41)	-6.88 (-105.35, 30.7)	-0.58 (-34.01, 69.89)
0.38 (-6.08, 6.36)	BCAA	-3.01 (-10.05, 3.69)	-1.39 (-6.71, 3.51)	-2.52 (-120.6, 29.66)	0.2 (-5.18, 5.48)	-2.98 (-9.27, 2.99)	5.15 (-58.49, 332.6)	-3.47 (-75.8, 48.97)	-4.49 (-97.13, 39.01)	-1.57 (-61.3, 80.4)	-3.49 (-8.24, 0.86)	-6.58 (-105.2, 30.9)	-0.27 (-33.72, 70.25)
3.39 (-3.33, 9.99)	3.01 (-3.69, 10.05)	BRT	1.61 (-4.05, 7.24)	0.52 (-117.43, 32.75)	3.21 (-2.98, 9.64)	0.01 (-6.56, 6.65)	8.22 (-55.96, 334.1)	-0.4 (-72.85, 51.96)	-1.42 (-94.15, 42.19)	1.47 (-58.4, 83.5)	-0.49 (-5.63, 4.66)	-3.59 (-101.57, 34.12)	2.8 (-30.65, 73.28)
1.76 (-3.09, 6.55)	1.39 (-3.51, 6.71)	-1.61 (-7.24, 4.05)	CAF	-1.09 (-119.08, 31.04)	1.58 (-2.62, 6.04)	-1.59 (-5.61, 2.43)	6.56 (-56.92, 333.39)	-2.01 (-74.42, 50.32)	-3.01 (-95.9, 40.35)	-0.11 (-59.68, 81.86)	-2.11 (-4.41, 0.21)	-5.07 (-103.69, 32.23)	1.18 (-31.98, 71.9)
2.88 (-29.22, 120.91)	2.52 (-29.66, 120.6)	-0.52 (-32.75, 117.43)	1.09 (-31.04, 119.08)	CHO	2.7 (-29.36, 120.72)	-0.47 (-32.7, 117.43)	10.32 (-72.79, 330.85)	0.54 (-86.19, 116.03)	-2.39 (-95.46, 122.52)	1.69 (-63.52, 137.2)	-1.03 (-33.13, 117.01)	-5.76 (-88.52, 123.41)	3.41 (-43.74, 117.1)

0.18 (-5.58, 5.57)	-0.2 (-5.48, 5.18)	-3.21 (-9.64, 2.98)	-1.58 (-6.04, 2.62)	-2.7 (-120.72, 29.36)	CRT	-3.19 (-8.79, 2.22)	4.97 (-58.79, 331.38)	-3.62 (-76.11, 48.63)	-4.64 (-97.4, 38.72)	-1.75 (-61.4, 80.33)	-3.7 (-7.45, -0.14)	-6.73 (-105.08, 30.59)	-0.45 (-33.67, 70.29)
3.35 (-2.57, 9.2)	2.98 (-2.99, 9.27)	-0.01 (-6.65, 6.56)	1.59 (-2.43, 5.61)	0.47 (-117.43, 32.7)	3.19 (-2.22, 8.79)	ED	8.2 (-55.43, 335.36)	-0.43 (-72.93, 51.98)	-1.43 (-94.25, 41.98)	1.44 (-58.24, 83.58)	-0.51 (-4.63, 3.6)	-3.55 (-101.72, 34.02)	2.74 (-30.57, 73.31)
-4.82 (-331.75 , 58.9)	-5.15 (-332.6, 58.49)	-8.22 (-334.1, 55.96)	-6.56 (-333.39, 56.92)	-10.32 (-330.85, 72.79)	-4.97 (-331.38, 58.79)	-8.2 (-335.36 , 55.43)	HMB	-10.47 (-373.19, 80.31)	-12.8 (-338.16, 77.96)	-8.72 (-285.71, 103.66)	-8.67 (-335.33, 54.82)	-16.44 (-342.53, 69)	-6.52 (-334.15, 97.63)
3.78 (-48.52, 76.23)	3.47 (-48.97, 75.8)	0.4 (-51.96, 72.85)	2.01 (-50.32, 74.42)	-0.54 (-116.03, 86.19)	3.62 (-48.63, 76.11)	0.43 (-51.98, 72.93)	10.47 (-80.31, 373.19)	LC	-0.89 (-112.19, 87.53)	2.6 (-74.75, 113.87)	-0.1 (-52.31, 72.26)	-3.03 (-106.7, 72.45)	3.61 (-57.19, 102.02)
4.78 (-38.69, 97.36)	4.49 (-39.01, 97.13)	1.42 (-42.19, 94.15)	3.01 (-40.35, 95.9)	2.39 (-122.52, 95.46)	4.64 (-38.72, 97.4)	1.43 (-41.98, 94.25)	12.8 (-77.96, 338.16)	0.89 (-87.53, 112.19)	MG	4.19 (-64.65, 120.03)	0.88 (-42.43, 93.75)	-2.51 (-104.73, 91.09)	4.99 (-47.68, 114.72)
1.89 (-80.2, 61.65)	1.57 (-80.4, 61.3)	-1.47 (-83.5, 58.4)	0.11 (-81.86, 59.68)	-1.69 (-137.2, 63.52)	1.75 (-80.33, 61.4)	-1.44 (-83.58, 58.24)	8.72 (-103.66, 285.71)	-2.6 (-113.87, 74.75)	-4.19 (-120.03, 64.65)	PBC	-1.97 (-84.06, 57.55)	-6.9 (-122.91, 61.7)	2.19 (-83.5, 92.09)
3.86 (-0.41, 8.04)	3.49 (-0.86, 8.24)	0.49 (-4.66, 5.63)	2.11 (-0.21, 4.41)	1.03 (-117.01, 33.13)	3.7 (0.14, 7.45)	0.51 (-3.6, 4.63)	8.67 (-54.82, 335.33)	0.1 (-72.26, 52.31)	-0.88 (-93.75, 42.43)	1.97 (-57.55, 84.06)	PLA	-2.95 (-101.51, 34.28)	3.28 (-29.78, 73.92)
6.88 (-30.7, 105.35)	6.58 (-30.9, 105.2)	3.59 (-34.12, 101.57)	5.07 (-32.23, 103.69)	5.76 (-123.41, 88.52)	6.73 (-30.59, 105.08)	3.55 (-34.02, 101.72)	16.44 (-69, 342.53)	3.03 (-72.45, 106.7)	2.51 (-91.09, 104.73)	6.9 (-61.7, 122.91)	2.95 (-34.28, 101.51)	PRT	9.08 (-47.56, 107.72)
0.58 (-69.89,	0.27 (-70.25,	-2.8 (-73.28,	-1.18 (-71.9,	-3.41 (-117.1,	0.45 (-70.29,	-2.74 (-73.31,	6.52 (-97.63,	-3.61 (-102.02,	-4.99 (-114.72,	-2.19 (-92.09,	-3.28 (-73.92,	-9.08 (-107.72,	RHO

34.01)	33.72)	30.65)	31.98)	43.74)	33.67)	30.57)	334.15)	57.19)	47.68)	83.5)	29.78)	47.56)	

(B)Lower limb Peak Power

BA	-0.98 (-1.99, 0.07)	-1.39 (-7.83, 4.02)	-0.55 (-7.59, 44.2)	-1.22 (-5.15, 7.01)	-0.99 (-12.27, 3.58)	-1.67 (-5.26, 5.81)	-1.39 (-7.21, 4.59)	-1.33 (-2.24, -0.42)
0.98 (-0.07, 1.99)	CAF	-0.42 (-6.83, 4.91)	0.39 (-6.62, 45.24)	-0.24 (-4.13, 8.02)	-0.01 (-11.26, 4.61)	-0.7 (-4.22, 7)	-0.4 (-6.18, 5.5)	-0.36 (-0.85, 0.11)
1.39 (-4.02, 7.83)	0.42 (-4.91, 6.83)	CHO	1.26 (-7.43, 45.88)	0.28 (-6.51, 10.42)	0.36 (-12.03, 8.31)	-0.23 (-6.75, 9.27)	0.07 (-8.8, 8.92)	0.05 (-5.33, 6.45)
0.55 (-44.2, 7.59)	-0.39 (-45.24, 6.62)	-1.26 (-45.88, 7.43)	CRT	-0.77 (-45.52, 10.34)	-0.84 (-45.32, 8.32)	-1.26 (-46.12, 8.52)	-1.07 (-45.53, 7.74)	-0.74 (-45.62, 6.25)
1.22 (-7.01, 5.15)	0.24 (-8.02, 4.13)	-0.28 (-10.42, 6.51)	0.77 (-10.34, 45.52)	ED	0.09 (-12.96, 6.34)	-0.48 (-9.26, 8.18)	-0.19 (-11.01, 7.85)	-0.11 (-8.33, 3.76)
0.99 (-3.58, 12.27)	0.01 (-4.61, 11.26)	-0.36 (-8.31, 12.03)	0.84 (-8.32, 45.32)	-0.09 (-6.34, 12.96)	HMB	-0.6 (-6.62, 12.73)	-0.32 (-8.22, 13.2)	-0.35 (-4.93, 10.85)
1.67 (-5.81, 5.26)	0.7 (-7, 4.22)	0.23 (-9.27, 6.75)	1.26 (-8.52, 46.12)	0.48 (-8.18, 9.26)	0.6 (-12.73, 6.62)	MG	0.28 (-9.54, 7.11)	0.34 (-7.32, 3.83)
1.39 (-4.59, 7.21)	0.4 (-5.5, 6.18)	-0.07 (-8.92, 8.8)	1.07 (-7.74, 45.53)	0.19 (-7.85, 11.01)	0.32 (-13.2, 8.22)	-0.28 (-7.11, 9.54)	PBC	0.05 (-5.87, 5.81)
1.33 (0.42, 2.24)	0.36 (-0.11, 0.85)	-0.05 (-6.45, 5.33)	0.74 (-6.25, 45.62)	0.11 (-3.76, 8.33)	0.35 (-10.85, 4.93)	-0.34 (-3.83, 7.32)	-0.05 (-5.81, 5.87)	PLA

(C)Lower limb Mean Power

BA	-0.02 (-3.45, 3.65)	-0.25 (-7.05, 5.76)	0.02 (-8.7, 5.99)	-0.17 (-5.83, 5.29)	0.18 (-6.07, 6.98)	0.71 (-4.76, 6.37)	-0.21 (-3.56, 3.36)

0.02 (-3.65, 3.45)	CAF	-0.24 (-5.53, 3.92)	0.06 (-7.75, 3.73)	-0.16 (-4.67, 3.64)	0.19 (-4.42, 5.14)	0.74 (-3.35, 4.29)	-0.19 (-1.19, 0.82)
0.25 (-5.76, 7.05)	0.24 (-3.92, 5.53)	CHO	0.27 (-9.14, 7.31)	0.09 (-6.47, 6.55)	0.43 (-6.19, 8.26)	0.97 (-5.53, 8)	0.05 (-4.11, 5.25)
-0.02 (-5.99, 8.7)	-0.06 (-3.73, 7.75)	-0.27 (-7.31, 9.14)	CRT	-0.22 (-6.68, 9.04)	0.14 (-6.47, 10.56)	0.68 (-5.22, 10.63)	-0.26 (-3.85, 7.5)
0.17 (-5.29, 5.83)	0.16 (-3.64, 4.67)	-0.09 (-6.55, 6.47)	0.22 (-9.04, 6.68)	ED	0.35 (-5.92, 7.75)	0.89 (-4.7, 7.01)	-0.03 (-3.7, 4.44)
-0.18 (-6.98, 6.07)	-0.19 (-5.14, 4.42)	-0.43 (-8.26, 6.19)	-0.14 (-10.56, 6.47)	-0.35 (-7.75, 5.92)	GPJ	0.53 (-6.13, 6.77)	-0.38 (-5.35, 4.2)
-0.71 (-6.37, 4.76)	-0.74 (-4.29, 3.35)	-0.97 (-8, 5.53)	-0.68 (-10.63, 5.22)	-0.89 (-7.01, 4.7)	-0.53 (-6.77, 6.13)	HMB	-0.93 (-4.38, 3.15)
0.21 (-3.36, 3.56)	0.19 (-0.82, 1.19)	-0.05 (-5.25, 4.11)	0.26 (-7.5, 3.85)	0.03 (-4.44, 3.7)	0.38 (-4.2, 5.35)	0.93 (-3.15, 4.38)	PLA

Table S13.2 League table of non-elite volleyball athletes

(A)Vertical Jump

BA	-1.65 (-10.07, 7.04)	-4.48 (-12.43, 3.54)	-3.05 (-8.77, 2.76)	-5.01 (-248.38, 61.09)	-1.98 (-9.04, 5.06)	-4.78 (-11.69, 2.26)	0.63 (-9.02, 10.32)	-7.08 (-155.65, 100.75)	-3.54 (-11.76, 4.85)	-2.77 (-12.6, 7.24)	-5.46 (-10.02, -0.75)	-12.15 (-216.28, 63.41)	-0.13 (-67.59, 145.22)
1.65 (-7.04, 10.07)	BCAA	-2.83 (-12.59, 6.76)	-1.4 (-9.38, 6.47)	-3.42 (-247.23, 62.51)	-0.32 (-9.33, 8.41)	-3.14 (-12.06, 5.69)	2.25 (-8.87, 13.28)	-5.49 (-153.59, 102.38)	-1.89 (-11.86, 7.99)	-1.12 (-12.4, 10.2)	-3.82 (-11, 3.28)	-10.59 (-214.76, 65.21)	1.57 (-67.14, 146.69)
4.48 (-3.54, 12.43)	2.83 (-6.76, 12.59)	BRT	1.43 (-5.93, 8.9)	-0.58 (-243.82, 65.55)	2.5 (-5.91, 10.83)	-0.29 (-8.59, 8.12)	5.09 (-5.56, 15.77)	-2.71 (-150.97, 105.1)	0.94 (-8.51, 10.48)	1.71 (-9.13, 12.69)	-0.99 (-7.43, 5.61)	-7.69 (-211.81, 68.38)	4.38 (-63.75, 149.85)

3.05 (-2.76, 8.77)	1.4 (-6.47, 9.38)	-1.43 (-8.9, 5.93)	CAF	-1.92 (-245.74, 63.98)	1.07 (-5.36, 7.41)	-1.73 (-7.03, 3.61)	3.66 (-5.51, 12.79)	-4.04 (-152.41, 103.73)	-0.49 (-8.23, 7.25)	0.28 (-9.15, 9.76)	-2.4 (-5.94, 1.1)	-8.99 (-213.42, 66.42)	2.93 (-64.64, 148.45)
5.01 (-61.09, 248.38)	3.42 (-62.51, 247.23)	0.58 (-65.55, 243.82)	1.92 (-63.98, 245.74)	CHO	3.08 (-63.01, 246.49)	0.21 (-65.91, 243.8)	5.6 (-60.47, 249.24)	0.73 (-177.25, 238.99)	1.51 (-64.65, 244.9)	2.3 (-63.96, 245.74)	-0.41 (-66.4, 243.07)	-10.87 (-181.5, 255.63)	7.01 (-88.81, 242.33)
1.98 (-5.06, 9.04)	0.32 (-8.41, 9.33)	-2.5 (-10.83, 5.91)	-1.07 (-7.41, 5.36)	-3.08 (-246.49, 63.01)	CRT	-2.81 (-10.24, 4.71)	2.59 (-7.33, 12.59)	-5.09 (-153.83, 102.55)	-1.57 (-10.18, 7.23)	-0.78 (-10.97, 9.57)	-3.48 (-8.68, 1.9)	-10.12 (-213.67, 65.52)	1.91 (-65.91, 147.37)
4.78 (-2.26, 11.69)	3.14 (-5.69, 12.06)	0.29 (-8.12, 8.59)	1.73 (-3.61, 7.03)	-0.21 (-243.8, 65.91)	2.81 (-4.71, 10.24)	ED	5.39 (-4.53, 15.29)	-2.31 (-150.74, 105.33)	1.24 (-7.39, 9.96)	2.02 (-8.23, 12.23)	-0.68 (-5.93, 4.6)	-7.38 (-211.67, 68.18)	4.67 (-63.14, 150.23)
-0.63 (-10.32, 9.02)	-2.25 (-13.28, 8.87)	-5.09 (-15.77, 5.56)	-3.66 (-12.79, 5.51)	-5.6 (-249.24, 60.47)	-2.59 (-12.59, 7.33)	-5.39 (-15.29, 4.53)	HMB	-7.73 (-157.16, 99.93)	-4.15 (-15.07, 6.83)	-3.37 (-15.54, 8.86)	-6.07 (-14.54, 2.38)	-12.89 (-216.31, 62.79)	-0.75 (-69.02, 145.29)
7.08 (-100.75, 155.65)	5.49 (-102.38, 153.59)	2.71 (-105.1, 150.97)	4.04 (-103.73, 152.41)	-0.73 (-238.99, 177.25)	5.09 (-102.55, 153.83)	2.31 (-105.33, 150.74)	7.73 (-99.93, 157.16)	LC	3.64 (-103.89, 151.8)	4.46 (-103.14, 152.66)	1.64 (-106.13, 149.94)	-5.05 (-219.5, 150.34)	7.92 (-116.05, 210.83)
3.54 (-4.85, 11.76)	1.89 (-7.99, 11.86)	-0.94 (-10.48, 8.51)	0.49 (-7.25, 8.23)	-1.51 (-244.9, 64.65)	1.57 (-7.23, 10.18)	-1.24 (-9.96, 7.39)	4.15 (-6.83, 15.07)	-3.64 (-151.8, 103.89)	MG	0.78 (-10.36, 11.88)	-1.92 (-8.82, 4.98)	-8.72 (-212.5, 67.06)	3.44 (-64.68, 148.81)
2.77 (-7.24, 12.6)	1.12 (-10.2, 12.4)	-1.71 (-12.69, 9.13)	-0.28 (-9.76, 9.15)	-2.3 (-245.74, 63.96)	0.78 (-9.57, 10.97)	-2.02 (-12.23, 8.23)	3.37 (-8.86, 15.54)	-4.46 (-152.66, 103.14)	-0.78 (-11.88, 10.36)	PBC	-2.7 (-11.48, 6.07)	-9.52 (-213.49, 66.49)	2.63 (-65.32, 148.46)
5.46 (0.75,	3.82 (-3.28,	0.99 (-5.61,	2.4 (-1.1, 5.94)	0.41 (-243.07,	3.48 (-1.9,	0.68 (-4.6,	6.07 (-2.38,	-1.64 (-149.94,	1.92 (-4.98,	2.7 (-6.07,	PLA	-6.58 (-210.76,	5.35 (-62.26,

10.02)	11)	7.43)		66.4)	8.68)	5.93)	14.54)	106.13)	8.82)	11.48)		68.81)	150.48)
12.15 (-63.41, 216.28)	10.59 (-65.21, 214.76)	7.69 (-68.38, 211.81)	8.99 (-66.42, 213.42)	10.87 (-255.63, 181.5)	10.12 (-65.52, 213.67)	7.38 (-68.18, 211.67)	12.89 (-62.79, 216.31)	5.05 (-150.34, 219.5)	8.72 (-67.06, 212.5)	9.52 (-66.49, 213.49)	6.58 (-68.81, 210.76)	PRT	17.91 (-96.87, 222.42)
0.13 (-145.22, 67.59)	-1.57 (-146.69, 67.14)	-4.38 (-149.85, 63.75)	-2.93 (-148.45, 64.64)	-7.01 (-242.33, 88.81)	-1.91 (-147.37, 65.91)	-4.67 (-150.23, 63.14)	0.75 (-145.29, 69.02)	-7.92 (-210.83, 116.05)	-3.44 (-148.81, 64.68)	-2.63 (-148.46, 65.32)	-5.35 (-150.48, 62.26)	-17.91 (-222.42, 96.87)	RHO

(B)Lower limb Peak Power

BA	-1.35 (-2.7, 0.06)	-1.88 (-11.83, 6.41)	-0.75 (-11.59, 68.94)	-1.78 (-3.33, -0.16)	-1.33 (-2.83, 0.21)	-2.14 (-3.67, -0.55)	-1.84 (-3.47, -0.15)	-1.79 (-3.01, -0.56)
1.35 (-0.06, 2.7)	CAF	-0.54 (-10.47, 7.68)	0.55 (-10.26, 70.37)	-0.42 (-1.64, 0.74)	0.02 (-1.08, 1.11)	-0.78 (-1.97, 0.35)	-0.48 (-1.79, 0.8)	-0.44 (-1.09, 0.18)
1.88 (-6.41, 11.83)	0.54 (-7.68, 10.47)	CHO	1.77 (-11.64, 71.2)	0.1 (-8.2, 10.02)	0.56 (-7.77, 10.47)	-0.26 (-8.55, 9.68)	0.04 (-8.25, 10.02)	0.08 (-8.18, 9.98)
0.75 (-68.94, 11.59)	-0.55 (-70.37, 10.26)	-1.77 (-71.2, 11.64)	CRT	-0.99 (-70.83, 9.87)	-0.56 (-70.37, 10.32)	-1.36 (-71.2, 9.5)	-1.06 (-70.85, 9.79)	-0.99 (-70.84, 9.8)
1.78 (0.16, 3.33)	0.42 (-0.74, 1.64)	-0.1 (-10.02, 8.2)	0.99 (-9.87, 70.83)	ED	0.45 (-0.9, 1.79)	-0.36 (-1.76, 1.04)	-0.06 (-1.57, 1.45)	-0.02 (-1.03, 0.99)
1.33 (-0.21, 2.83)	-0.02 (-1.11, 1.08)	-0.56 (-10.47, 7.77)	0.56 (-10.32, 70.37)	-0.45 (-1.79, 0.9)	HMB	-0.81 (-2.13, 0.5)	-0.51 (-1.93, 0.93)	-0.47 (-1.36, 0.42)
2.14 (0.55, 3.67)	0.78 (-0.35, 1.97)	0.26 (-9.68, 8.55)	1.36 (-9.5, 71.2)	0.36 (-1.04, 1.76)	0.81 (-0.5, 2.13)	MG	0.3 (-1.18, 1.78)	0.34 (-0.62, 1.31)
1.84 (0.15, 3.47)	0.48 (-0.8, 1.79)	-0.04 (-10.02, 8.25)	1.06 (-9.79, 70.85)	0.06 (-1.45, 1.57)	0.51 (-0.93, 1.93)	-0.3 (-1.78, 1.18)	PBC	0.04 (-1.09, 1.16)
1.79 (0.56,	0.44 (-0.18,	-0.08 (-9.98,	0.99 (-9.8,	0.02 (-0.99,	0.47 (-0.42,	-0.34 (-1.31,	-0.04 (-1.16,	PLA

3.01)	1.09)	8.18)	70.84)	1.03)	1.36)	0.62)	1.09)	

(C)Lower limb Mean Power

BA	0.03 (-1.83, 1.87)	-0.25 (-9.4, 6.9)	0.01 (-13.6, 6.26)	-0.17 (-2.05, 1.68)	0.16 (-7.85, 8.75)	0.73 (-1.18, 2.64)	-0.21 (-1.6, 1.16)
-0.03 (-1.87, 1.83)	CAF	-0.28 (-9.43, 6.87)	-0.01 (-13.65, 6.12)	-0.2 (-1.96, 1.56)	0.13 (-7.8, 8.65)	0.71 (-1.1, 2.52)	-0.24 (-1.47, 0.99)
0.25 (-6.9, 9.4)	0.28 (-6.87, 9.43)	CHO	0.22 (-16.3, 12.36)	0.08 (-7.12, 9.2)	0.41 (-11.11, 13.98)	0.98 (-6.24, 10.18)	0.04 (-7.1, 9.06)
-0.01 (-6.26, 13.6)	0.01 (-6.12, 13.65)	-0.22 (-12.36, 16.3)	CRT	-0.2 (-6.4, 13.45)	0.16 (-11.27, 18.31)	0.71 (-5.5, 14.3)	-0.24 (-6.34, 13.36)
0.17 (-1.68, 2.05)	0.2 (-1.56, 1.96)	-0.08 (-9.2, 7.12)	0.2 (-13.45, 6.4)	ED	0.34 (-7.6, 8.94)	0.91 (-0.93, 2.75)	-0.04 (-1.3, 1.23)
-0.16 (-8.75, 7.85)	-0.13 (-8.65, 7.8)	-0.41 (-13.98, 11.11)	-0.16 (-18.31, 11.27)	-0.34 (-8.94, 7.6)	GPJ	0.57 (-8.02, 8.55)	-0.38 (-8.94, 7.54)
-0.73 (-2.64, 1.18)	-0.71 (-2.52, 1.1)	-0.98 (-10.18, 6.24)	-0.71 (-14.3, 5.5)	-0.91 (-2.75, 0.93)	-0.57 (-8.55, 8.02)	HMB	-0.95 (-2.28, 0.38)
0.21 (-1.16, 1.6)	0.24 (-0.99, 1.47)	-0.04 (-9.06, 7.1)	0.24 (-13.36, 6.34)	0.04 (-1.23, 1.3)	0.38 (-7.54, 8.94)	0.95 (-0.38, 2.28)	PLA

Abbreviations: PLA, Placebo; BA, β-Alanine; BCAA, Branched-Chain Amino Acids; CHO, Carbohydrate; CRT, Creatine; BRT, Beetroot Juice; PBC, Probiotics; MG, Magnesium; HMB, β-Hydroxy-β-Methylbutyrate; CAF, Caffeine; ED, Energy Drink; PRT, Protein; GPJ, Grape Juice; LC, L-Citrulline; VJ, Vertical jump; PP, Lower limb peak power; MP, Lower limb mean power;

Appendix 14: Funnel Plots and Egger Tests

Figure S14.1 Funnel Plot and Egger Tests of Vertical Jump

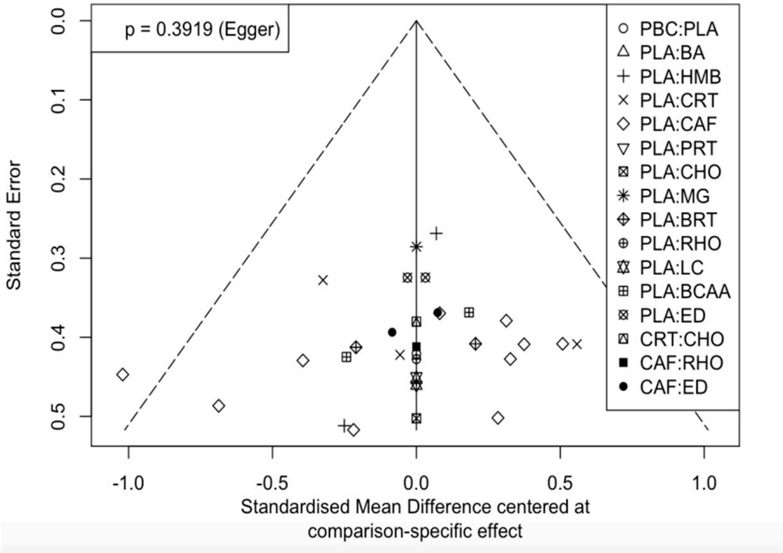

Figure S14.2 Funnel Plot and Egger Tests of Lower limb Peak Power

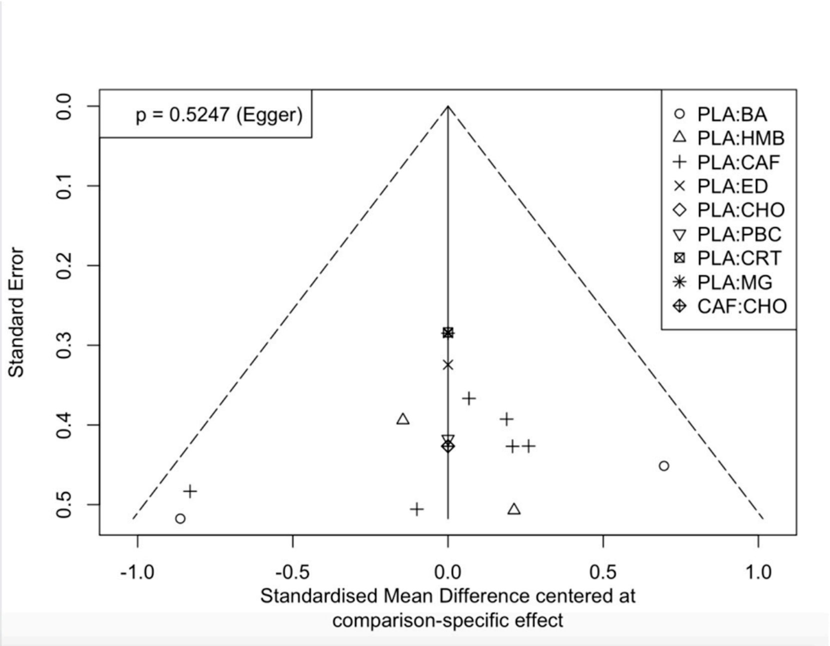

Figure S14.3 Funnel Plot of and Egger Tests Lower limb Mean Power

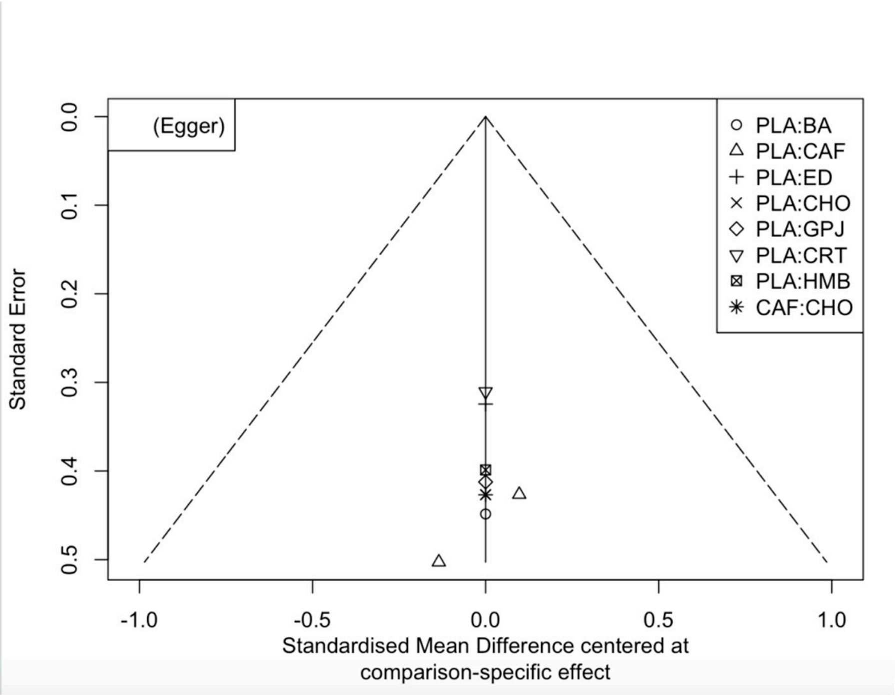
(Egger)
PLA:BA
PLA:CAF
PLA:ED
PLA:CHO
PLA:GPJ
PLA:CRT
PLA:HMB
CAF:CHO
Standard Error
0.0
0.1
0.2
0.3
0.4
0.5
-1.0
-0.5
0.0
0.5
1.0
Standardised Mean Difference centered at
comparison-specific effect
